# Unraveling Particle Formation: From Single Droplet Drying to Spray Drying and Electrospraying

**DOI:** 10.3390/pharmaceutics12070625

**Published:** 2020-07-04

**Authors:** Eline Boel, Robin Koekoekx, Sien Dedroog, Iurii Babkin, Maria Rosaria Vetrano, Christian Clasen, Guy Van den Mooter

**Affiliations:** 1 Department of Pharmaceutical and Pharmacological Sciences, Drug Delivery and Disposition, KU Leuven, 3000 Leuven, Belgium; eline.boel@kuleuven.be (E.B.); sien.dedroog@kuleuven.be (S.D.); 2 Department of Chemical Engineering, Soft Matter, Rheology and Technology, KU Leuven, 3001 Leuven, Belgium; robin.koekoekx@kuleuven.be (R.K.); iurii.babkin@kuleuven.be (I.B.); christian.clasen@kuleuven.be (C.C.); 3 Department of Mechanical Engineering, Applied Mechanics and Energy Conversion, KU Leuven, 3001 Leuven, Belgium; rosaria.vetrano@kuleuven.be

**Keywords:** single droplet drying, spray drying, electrospraying

## Abstract

Spray drying and electrospraying are well-established drying processes that already have proven their value in the pharmaceutical field. However, there is currently still a lack of knowledge on the fundamentals of the particle formation process, thereby hampering fast and cost-effective particle engineering. To get a better understanding of how functional particles are formed with respect to process and formulation parameters, it is indispensable to offer a comprehensive overview of critical aspects of the droplet drying and particle formation process. This review therefore closely relates single droplet drying to pharmaceutical applications. Although excellent reviews exist of the different aspects, there is, to the best of our knowledge, no single review that describes all steps that one should consider when trying to engineer a certain type of particle morphology. The findings presented in this article have strengthened the predictive value of single droplet drying for pharmaceutical drying applications like spray drying and electrospraying. Continuous follow-up of the particle formation process in single droplet drying experiments hence allows optimization of manufacturing processes and particle engineering approaches and acceleration of process development.

## 1. Introduction

The need for more sophisticated particles for pharmaceuticals, food products, ceramics, and catalysis has increased in recent years [1]. The variety of particles that can be produced inquires particle engineering approaches to control the particle morphology, size distribution, porosity, density, composition, and purity of the particles [2]. Changes in particle morphology can have implications for their physicochemical properties such as their reactivity, solubility, taste masking, flowability, and wettability. Particle morphologies can diversify from, for example, hollow, low density particles to particles that exist out of smaller subunits such as nanoparticles [3]. A first step towards controlling particle characteristics is gaining a deeper understanding of the particle formation process itself.

One way to create particles is by applying drying processes to solution droplets of the particle material. These processes are one of the most important and most energy-consuming industrial operations [4]. Although drying techniques have a wide applicability, this review will only focus on the drying process occurring in the pharmaceutical field. More specifically, spray drying and electrospraying will be highlighted. The basic principle of both methods is that a solution is prepared by dissolving all components in a common solvent, followed by atomization in small droplets. Solvent evaporation will take place at the droplet surface and, after drying is completed, the final particles can be collected [5]. The difference between both techniques is that, in the case of spray drying, drying occurs at an increased temperature, while in the case of electrospraying the application of a high voltage leading to small droplets makes it possible to dry the droplets at room temperature. Although these are established drying techniques, there is still a lack of knowledge on the particle formation process [1].

To gain more insight in the drying mechanism, single droplet drying (SDD) techniques can be employed as a more simplified approach to controlled droplet formation and observation. It should be kept in mind that the droplet in SDD investigations will undergo a slightly different drying process due to a generally increased droplet diameter (i.e., mm instead of µm) and drying time (i.e., minutes instead of seconds). Next to this, the droplet will not experience droplet–droplet interactions, droplet-wet particle interactions or droplet-wall collisions [1,6]. Despite these differences, SDD methods have an added value to better understand and optimize pharmaceutical drying processes, as discussed further in this article in more detail. Drying of a single droplet can be studied using various techniques: using a filament, an acoustic levitator, levitation by air flow, or a free-fall technique. In this review, an overview of these techniques and their comparability towards spray drying and electrospraying can be found.

Irrespective of the applied droplet drying technique, the drying mechanisms for free solution droplets are similar. Before the actual drying process starts, the droplet will be heated (or cooled) until the wet-bulb temperature (i.e., the (lower) equilibrium temperature due to a constant evaporation heat loss of the solvent into the surrounding (warmer) air at a given pressure, temperature and relative humidity (RH)) is reached [7,8]. In a first stage of unhindered drying, the droplet drying is mainly characterized by the solvent, similar to evaporation of a pure solvent droplet. The driving force for solvent evaporation is the difference between the vapor pressure of the solvent and its partial pressure in the surrounding environment. However, the drying rate (generally expressed in kg m^−2^ s^−1^) is limited and controlled by the energy required for solvent evaporation and thus by the heat transport towards the surface of the droplet [9]. After heating of the droplet to the wet-bulb temperature, drying will start from the surface of the droplet [7]. As drying continues, solvent molecules will keep migrating from the center towards the surface. The migration of the solvent molecules can be mediated through molecular diffusion relative to the solute, convection of fluid within the droplet or capillary solvent flow through a solid porous matrix [5]. The unhindered drying that is analogous to free solvent evaporation results in droplet shrinkage. If the ambient parameters remain unchanged, the droplet temperature remains equal to the wet-bulb temperature and the drying rate is constant and only controlled by the heat transfer towards the surface of the droplet [10]. For this reason, this stage is named the constant-rate drying stage.

**Figure 1:**
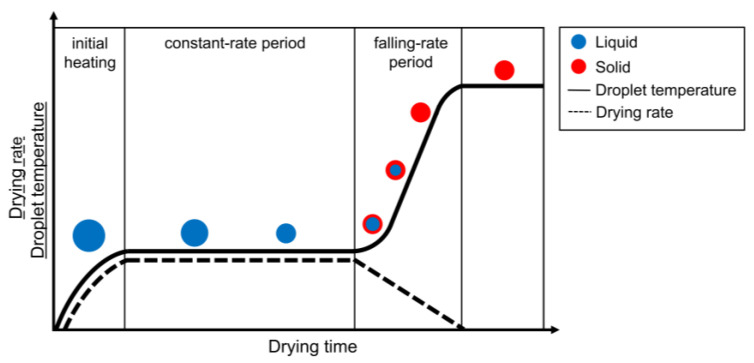
Schematic illustration of the different stages a droplet experiences during the drying process. Temperature evolution is represented by a solid line and drying rate by a dotted line. The liquid fraction is shown in blue and the solid fraction in red (modified from Mezhericher et al. [11,13]).

The second stage of drying is defined by the solutes present in the droplet. As solvent evaporation takes place from the surface of the droplet, the solute concentration at the surface increases, and the growing concentration gradient causes a diffusional solute flux away from the droplet surface towards the center of the droplet. The diffusional motion of the dissolved components is a complex phenomenon that needs to be understood correctly to explain the final distribution of the different substances [9]. In this review, the different factors that control this movement will be elaborated for single droplet drying, spray drying and electrospraying. Eventually, the diffusional motion of the solutes towards the center of the droplet becomes lower than the reduction rate of the droplet diameter due to the constant rate solvent loss. At this point, crust formation will occur due to solute enrichment at the droplet surface, leading to decreased drying rate and introducing the second drying stage [1]. This point is referred to as the critical point or locking point. At the start of the second drying stage, a porous solid crust and an internal wet core can be distinguished in the drying droplet. The drying rate is now controlled by the diffusion or capillary flow rate of the solvent from the wet core through (the pores of) the crust. The slowed solvent evaporation still results in shrinkage of the wet core and substantial growth of the crust towards the center of the droplet [1,11]. The thickening of the crust will lead to an increasing resistance for mass transfer and thereby a gradual reduction of the drying rate. Hence, this second stage is named the falling-rate stage of drying. During this stage, the droplet’s surface temperature will increase until the dry-bulb temperature (i.e., temperature of the surrounding gas) is reached and a non-evaporating solid sphere is developed [7]. This implies that a particle with the lowest possible amount of residual solvent is present, which can either be an equilibrium amount or residual solvent that cannot be removed by the drying process. In some cases, a clear subdivision of the falling-rate period based on the degree of drying rate decrease can be observed: a first stage, where unsaturated surface drying occurs, and a second stage, where the diffusion of fluid from the center of the droplet towards the surface is the rate limiting factor [12]. Hence, the drying rate over time in the falling-rate period can take on different shapes depending on the system and the drying conditions. The general two-stage droplet drying process is depicted in Figure 1 [11,13].

Pressure differences in- and outside the droplet during drying have a decisive impact on the final particle morphology. More specifically, the capillary pressure plays an important role in the drying process. During drying, solvent evaporation results in droplet shrinkage and the reduced radius leads to an increase in capillary and thus internal pressure of the droplet. At the critical point, further droplet shrinkage will be blocked by crust formation, but as solvent evaporation can continue through the porous crust, the pressure difference over the crust will keep increasing. The mechanical properties of the crust determine if it can withstand the pressure build-up and thereby the particle morphology [1,9,14]. Generally, drying of solution droplets that are of spherical shape due to the surface tension, results in spherically shaped particles. However, there is some diversity in particle morphology possible. The different morphologies and the factors that influence them will be described further in this review.

The focus of this work lies on a deeper understanding of this drying process to facilitate particle engineering. Hence, all aspects of the droplet drying process are described. Although excellent reviews exist for different features, there is, to the best of our knowledge, no single review that describes all steps that one should consider when trying to engineer a certain type of particle morphology. Techniques to create single droplets, as well as parameters that can influence the drying process and visualization techniques to characterize the changes during drying are discussed. With a view to pharmaceutical applications, the predictive power of single droplet drying for spray drying and electrospraying processes is described. Next to that, techniques to create sprays and their influence on droplet size distributions can be found. This review also comprises models for the simulation of both single droplet drying and spray drying. It should be pointed out that this work focuses on free solution droplets, meaning that there is no interference of solvent evaporation by a substrate and that a common solvent for all compounds is required. There is a lot of literature available on drying of droplets on diverse surfaces and on the drying behavior of dispersions and suspensions. Although these drying mechanisms are even more complex than that of free solution droplets, this review can also be employed as a starting point for gaining insight in their drying behavior. In addition, the combination of low molecular weight drugs and polymer systems are highlighted because of their widespread use in pharmaceutical industry. Articles concerning other types of dissolved substances were only included in case the conclusions or outcomes are believed to be generalizable towards pharmaceutical drying processes.

## 2. Physics of Particle Formation from a Drying Droplet

### 2.1. Methods to Create Single Droplets

**Figure 2:**
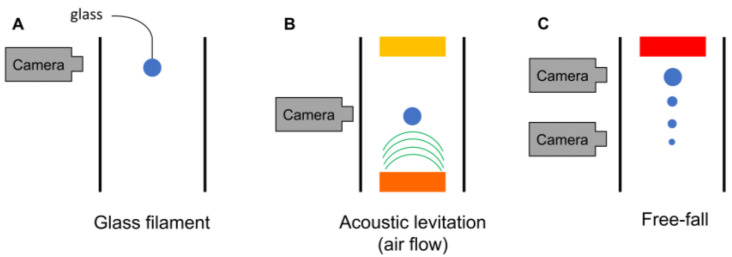
Schematic illustration of single droplet drying methods: (**A**) Glass filament, (**B**) Acoustic levitation or levitation by air flow, where both a transducer (orange) and a reflector (yellow) are needed to obtain acoustic waves (green), (**C**) Free-fall method, where a monodisperse droplet generator (red) produces the droplet (modified from de Souza Lima et al. [1]).

Drying of a single droplet can be impacted by the way it is generated. The main methods to create and dry single droplets are schematized in Figure 2. Based on their principles of operation, they can be divided in two categories, namely levitation and free-fall methods. In case of contact levitation, the droplet is suspended on a filament, while in the case of non-contact levitation methods, the droplet floats in the air due to an acoustic field or an air stream [1]. Several excellent reviews discuss the physical mechanisms involved in these techniques and the different types of information that can be gathered using them [1,14,15,16,17]. In Table 1, an overview of their principles, (dis)advantages, and similarity to pharmaceutical applications can be found. In the upcoming section, their principles will be discussed and their relevance for pharmaceutical industry will become clear from several examples. As this review focuses on the drying process of free droplets, drying of sessile droplets on a hydrophobic surface will not be discussed.

#### 2.1.1. Levitation Methods

Contact levitation is an intrusive method where the droplet is suspended at the tip of a glass filament or glass capillary tube and placed in a controlled air stream (see Figure 2a) [15]. By using this method, three important drying kinetics parameters are measured, namely droplet mass, temperature, and diameter. Mass changes can be measured by attaching the filament to a highly precision balance, thereby measuring the variation in droplet mass during drying. Alternatively, as a droplet deflects the top of the filament depending on its mass, the final deflection can be correlated to the loss in mass during drying. Finally, when the droplet is fixed, its shape evolution can be video recorded, and its diameter can be obtained via image processing. Despite the simplicity of this levitation method, its intrusiveness can impact the droplet drying process [1]. It was shown that the filament contributes to 1% of the heat transferred to a 1 mm droplet and it also had an effect on the final particle diameter and the distribution of the components in the particle [1,15,18].

Despite its intrusive nature, the glass filament technique was successfully applied to explore the drying kinetics of diverse compounds. For instance, it was used to gain insight in the shrinkage behavior of droplets, containing dairy products, by simultaneously monitoring the mass, diameter, morphology, and temperature changes during drying [19]. Lin et al. employed this technique to study the effect of the drying temperature, initial solute concentration, solubility, and latent heat of crystallization on the particle morphology by adding diverse model compounds [20].

An advantage of the glass filament technique is that it can be extended to test in situ dissolution of particles. Hereby, the drying process is discontinued and a solvent droplet is attached to the (semi)-dried particle, which can provide information about the surface wettability, dissolution rate and material disassociation [15]. Fu et al. tested the effect of two model compounds (i.e., sucrose and Rhodamine B) on the shell formation process and surface morphology of the particle [21]. Performing an in situ dissolution test enabled to investigate the influence of the model compounds on the solubility of the shell [21]. In another study, Fu et al. applied this method to investigate the crystallization behavior of lactose during droplet drying and the dissolution rate after drying, allowing to determine the impact of the drying temperature on the in situ dissolution behavior [22].

To reduce intrusiveness, either an acoustic field or aerodynamic levitation can be employed. The acoustic field is established using a transducer, which is attached to a piezoelectric crystal vibrating at an ultrasonic frequency, and a reflector (see Figure 2b). In the acoustic field, the droplet weight is in equilibrium with the upward acoustic radiation force [23]. This results in an upward movement of the droplet during drying due to solvent evaporation. Therefrom, a droplet drying curve can be developed [24]. Alternatively, continuous measurement of the RH of the moisture content by a dew point hygrometer also enables evaluation of the drying rate [17]. Next to the drying rate itself, an infrared thermometer and a video camera can be used to measure the droplet temperature and its morphological development, respectively [14]. When placing a droplet in an acoustic field by levitation, a secondary flow around the levitated droplet is generated by the acoustic waves. To avoid acoustic streaming effecting the heat and mass transfer during droplet drying, some authors applied an additional air flow in the acoustic levitator. Although this can overcome the streaming problem, it should be kept in mind that if the air flow rate is set too high, the droplet position can become unstable [23,25]. For an overview of other recent developments in the field of acoustic levitation, the reader is referred to a more specific review [26]. Acoustic levitation of water–glass suspension droplets was performed by Mondragon et al. [27]. In their work, the influence of air temperature, initial droplet volume, initial solid load and RH of the air was studied on the mean porosity of the particle using a central composite design [27]. Another example of levitation by an acoustic field is the evaluation of velocity and rotation of nanosuspension droplets using particle image velocimetry (PIV). Saha et al. performed detailed velocity measurements inside droplets with different diameters and viscosities [28].

Additionally, in the pharmaceutical field, this single droplet drying technique has already shown its effectiveness. Wulsten et al. applied it to study the drying kinetics of itraconazole and poly(vinylpyrrolidone-co-vinyl acetate) (PVP-VA) 64/hydroxypropylmethylcellulose (HPMC) in binary solvent mixtures of dichloromethane (DCM) and ethanol [29]. They showed that the polymeric compound determined the drying rate, while the microscopic analyses demonstrated that the drug influenced the morphology of the particle [29].

Droplet levitation can also be achieved using an air flow instead of an acoustic field. Such an aerodynamic levitator is not commonly used due to the difficulties in maintaining the droplet motionless [16].

#### 2.1.2. Free-Fall Methods

To exclude the interference of either a filament or an acoustic field on the drying kinetics, the free-fall method can be used. As its name already indicates, the droplet is not supported during the drying process, but freely falls through a drying chamber as a consequence of gravitational force. This implies that the drying kinetics monitored using this droplet drying technique are closest to pharmaceutical applications such as spray drying and electrospraying. A schematic illustration of the free-fall method is depicted in Figure 2c [15]. With this method it is not only possible to investigate one droplet at a time, since a chain of droplets with identical size and properties is also widely applied [1].

**Figure 3:**
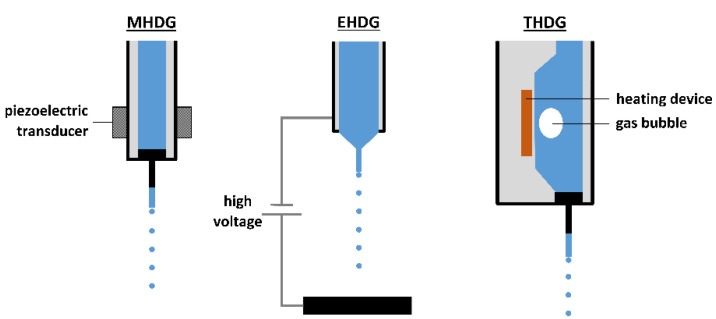
Schematic illustration of a mechano-hydrodynamic droplet generator (MHDG) equipped with a piezoelectric transducer, an electro-hydrodynamic droplet generator (EHDG) in (micro)dripping mode and a thermo-hydrodynamic droplet generator (THDG) (modified from Liu et al. [31]).

In free-fall methods, droplets are generally produced by a monodisperse droplet generator (MDG). Different types of MDGs exist and are subdivided in mechano-hydrodynamic droplet generators (MHDG), electro-hydrodynamic droplet generators (EHDG), thermo-hydrodynamic droplet generators (THDG), and microfluidic aerosol nozzles (MFAN), according to Wu et al. [30]. In MHDGs, a piezoelectric transducer is applied to break up the liquid stream into individual droplets, as illustrated in Figure 3. This type of MDG can also be implemented in a spray drying setup, in order to attain good control over the droplet size distribution. The use of MHDGs within spray drying is elaborated further in this review. Next to the piezoelectric MHDG, another type of MHDG can be distinguished, relying on the shear force of an externally flowing fluid [30,31]. Nevertheless, this one is not commonly used and therefore not further elaborated.

EHDG is generally referred to as electrohydrodynamic atomization or electrospraying because of the applied voltage between a nozzle tip and a collector adding surface charge to the pumped feed solution and facilitating the production of small droplets [32]. The (micro)dripping mode, which is prevalent at lower voltages than the cone-jet mode, is most often denoted in the context of MDGs. A schematic illustration of an EHDG in (micro)dripping mode can be seen in Figure 3. Electrospraying and the parameters that influence the operational modes and the final particle properties will be discussed in more detail further in this review.

THDGs, commonly used in the ink-jet printing industry, operate via a heating device that heats the liquid feed, as presented in Figure 3. In this way, vaporization occurs, leading to the formation of gas bubbles inside the nozzle and subsequent collapse upon condensation. Continuous formation and collapse of gas bubbles produces pressure waves that generate monodisperse droplets. However, compared to the other MDG types, this system takes longer to create a droplet [33].

Another type of MDG is the MFAN or the piezoelectric glass nozzle. In this system, dehumidified air is used to force a pre-filtered liquid feed through a capillary glass tube. This feed is then broken up into distinct droplets by the regular squeezing of the capillary tube using an annular piezoelectric element [30,34,35]. MFAN has proven its efficacy to produce uniform microparticles in a lab-scale microfluidic-jet spray dryer, as well as in a pilot-scale microfluidic-jet spray dryer [30,34,36].

A disadvantage of the free-fall method is that there is no possibility of direct measurement of mass or temperature. However, indirect measurements using image analysis remain a possibility to evaluate the evaporation rate from the droplet shrinkage [37]. Alternatively, the evaporation rate can be determined from the terminal velocity of the droplet [15]. Nevertheless, there is some literature available on complex analysis procedures to make direct measurements of the falling droplets using sampling ports at different positions in the drying chamber [15]. More general techniques for droplet characterization will be discussed further.

The free-fall method equipped with an MDG is successfully applied to gain insight in the drying kinetics of a broad range of materials. Rogers et al. studied the formation of insoluble material in milk powder during drying [38]. They applied the free-fall method to evaluate the drying behavior of monodisperse droplet streams, generated by a piezoelectric MHDG, containing skimmed milk and milk protein powders in multiple heat conditions [38]. Similarly, Fang et al. explored the effect of spray drying conditions in monodisperse droplet streams made of milk powder concentrate on their functionality, allowing them to find a direct correlation between the drying air temperature and the functionality of the particle [39]. Additionally, Vehring et al. applied this droplet drying technique and evaluated the evaporation rate by monitoring the droplet diameter [3]. As only indirect measurements were possible, they measured the changes in droplet diameter at different distances from the injection point using light scattering techniques [3].

It should be mentioned that this droplet drying method is not only beneficial to gain a deeper understanding of pharmaceutical drying processes, but this continuous, multiple droplet production technique is also successfully applied for formulation of particles as such. For instance, Amelia et al. used a MFAN to formulate uniform magnetic microcomposites of silica nanoparticles, iron oxide and lactose [40]. It can also be applied to assembly uniform microparticles of drugs and polymeric compounds, as reported by Liu et al. [41]. The uniform size of the microparticles was beneficial to find a correlation between the microstructure of the particles and the release mechanism of the drug, as there was no influence of different particle sizes on the release.

### 2.2. Parameters That Influence the Single Droplet Drying Process

The SDD techniques allow for investigation of the influence of a wide variety of different process and formulation parameters on the dried particle characteristics in a simplified manner. Applying general conclusions made for a certain system by these methods to pharmaceutical production processes like spray drying and electrospraying is not always straightforward, as will be discussed at length in Section 3. Still, SDD techniques are of great importance in delivering information and a theoretical framework for transition from an empirical approach to an actual particle engineering approach for a desired product. With regard to pharmaceuticals, the final average particle size, size distribution, and their morphology are some of the most important final particle characteristics. The average particle size and size distribution are largely dependent on the initial droplet size and size distribution, which is mainly process related, and on the initial solute concentration, as can be derived from a simple mass balance [9,42]:

(1) $$d_{p}=\sqrt[{3}]{\frac{c_{i}}{\rho_{p}}}\;d_{d}$$

where $d_{p}$ and $d_{d}$ [m] are the final particle and droplet diameter, respectively, $c_{i}$ [kg L^−1^] the initial feed solute concentration, and $\rho_{p}$ [kg m^−3^] is the particle density. With a view to pharmaceutical particle production processes, it is important to mention that this equation is only valid if there is no further droplet fission after initial droplet production and if no coalescence takes place during droplet flight. In that case, a wider particle size distribution can generally be observed.

The particle morphology is related to many different interdependent process and formulation parameters. In this regard, the dimensionless Peclet number (Pe), which indicates the ratio of the evaporation rate and the diffusion rate of the solute(s), is a convenient means to generalize the influence of many parameters and is often utilized to predict final particle morphology [9,14,32]:

(2) $$\mathrm{Pe}=\frac{d_{d}\;J}{D_{i}}$$

where J [m s^−1^] is volume loss per unit area or the evaporative flux and $D_{i}$ [m² s^−1^] is the diffusion coefficient of solute i in the solvent system. The Stokes-Einstein equation can be used to determine the diffusion coefficient of a solute ($D_{i}$) and shows the different factors that contribute to the molecular mobility of a solute in a solvent system [43]:

(3) $$D_{i}=\frac{k_{b}T}{6\pi \eta R_{h,i}}$$

where $k_{b}$ [J K^−1^] is the Boltzmann constant and $R_{h,i}$ [m] is the hydrodynamic radius of a molecule in the solvent system experiencing a dynamic viscosity η [Pa s] at a temperature T [K]. The value of Pe will indicate whether the evaporation rate is relatively faster compared to the diffusional motion of the solute(s) or vice versa. This knowledge allows for the prediction of the radial distribution of the solute(s) during droplet drying, which has major implications on the final dried particle morphology.

As indicated in the discussion of drying stages in Figure 1, the evaporation of the solvent at the droplet surface causes an increase in the local concentration of the solute(s) at the surface. The resulting concentration gradient creates a driving force for the molecules in the higher concentration region to diffuse towards the center of the droplet to homogenize the concentration in the entire droplet. At high Pe values (Pe>1), the solvent removal is faster than the diffusional motion of the solute(s), and the solute(s) concentration close to the surface will strongly and non-linearly increase, thereby generating a concentration gradient. Eventually, a critical concentration of solute(s) at the surface can be reached at which the solute mobility is locked in, resulting in a skin/crust formation localized at the surface as the location of the largest local concentration gradient. This locking point and crust formation generally occur earlier in the droplet lifetime for lower solute saturation limits in a specific solvent, and for higher Pe numbers. After the onset of skin/crust formation, the drying process continues for the residual liquid solvent inside the shell. During the second drying stage after skin/crust formation, as indicated in Figure 1, the evaporation rate decreases rapidly due to the increased heat and mass transport resistances of the crust. The mechanical properties and porosity of the solid crust depend on the solute(s) characteristics and on which solidification or locking mechanism is triggered at that critical concentration, resulting in a wide variety of different morphologies. Hollow particles can be formed if the crust is strong but porous enough for the residual solvent in the core to still evaporate without the collapse of the outer solid layer. Buckled/collapsed particles are prominent when the crust cannot withstand the pressure difference caused by the continued internal solvent evaporation. Wrinkled and more irregularly shaped particles are also generated at higher Pe values if the zones of increased concentration lead to an auto-acceleration of particle mobility blockage, such that slight inhomogeneities of concentration distributions over the surface amplify and thus lead to non-uniform droplet drying conditions and wrinkling across the surface.

On the other hand, at low Pe values (Pe<1), the diffusion rate of the solute(s) is sufficient to avoid a strong enrichment at the receding surface. The concentration gradients are low, and the dissolved solutes remain relatively homogeneously dispersed inside the droplet. In this case, more smooth and spherical particles will be generated due to the lack of early crust formation and uniform drying rates over the whole droplet surface. The generated particles are typically smaller and denser compared to particles dried at high Pe values [9,14,32].

**Figure 4:**
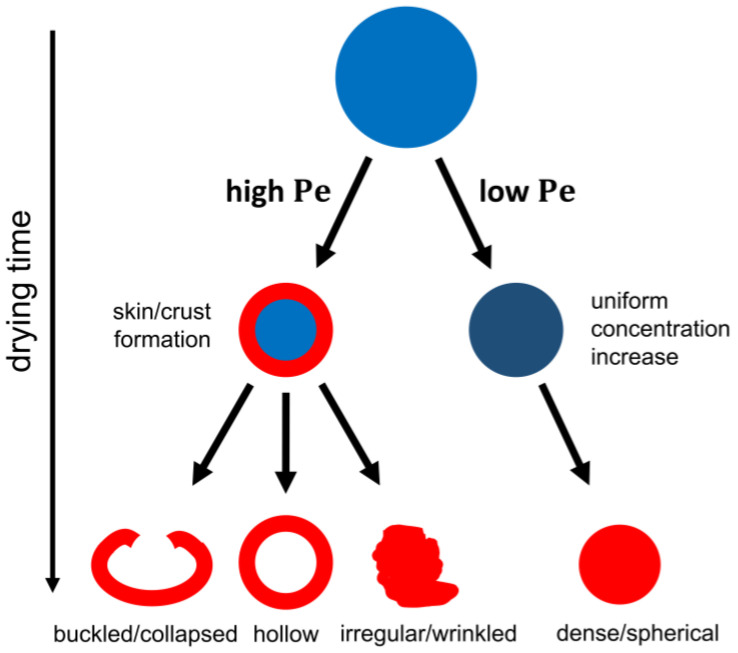
Schematic illustration of the influence of the drying conditions and formulation properties/composition (represented in Pe) on the final morphology.

A simplified schematic representation of these two cases is given in Figure 4. In reality, droplets will experience varying values for the Pe number during their lifetime due to changing evaporation rates and diffusion coefficients, complicating the above discussion. Nevertheless, this concept allows for physical interpretation of the effect that different process and formulation factors will have on final particle morphologies in droplet drying processes. In the following section, the influence of various parameters on the droplet drying and particle formation process is discussed in greater detail.

#### 2.2.1. Drying Conditions

##### Temperature

One of the most influential parameters on evaporation processes is the drying temperature. While it is the evaporation rate that enters the Pe number in Equation (2), the drying rate itself is usually determined by the heat transfer rate from the gas to the droplet surface. The heat transfer rate, the limiting factor for drying rate, is proportional to the wet-bulb depression, the temperature difference ΔT between the temperature of the gas $T_{gas}$ and the wet-bulb temperature $T_{wb}$ of the droplet, which is lower since the evaporation of the solvent draws heat from the droplet. For a given RH of the gas, ΔT increases with $T_{gas}$ (as can be derived from psychrometric charts) so that the evaporation rate and thus Pe increases. However, the temperature will not only directly influence the evaporation rate of the solvent. It also has an effect on the molecular mobility of the solute, as is evident from Equation (3). Still, the evaporation rate will generally increase more drastically with temperature than the diffusion coefficient of the solute(s), leading to higher Pe values with increasing temperature. This increase of Pe results in the above discussed fast increase of solute concentration close to the surface, crust formation and the resulting more irregularly shaped, hollow or collapsed particles.

An additional effect of the temperature that is not captured by the Pe number is the relative position of the droplet’s drying temperature to the boiling point of the involved solvent [44,45,46]. When the temperature exceeds the boiling point, vapor generated in the core of the droplet can open the solidified skin to be released, with a subsequent healing of these openings. This can occur several times until the crust is strong enough to withstand this cycle. Depending on the mechanical properties of the formed crust, it can not only crack, releasing the internal buildup of vapor, but also inflate, thin, and eventually explode, leaving open shells with large cavities or disintegrating the particle completely into fragments [8]. To capture drying environments with a temperature higher than the boiling point of the solvent, Dolinski and Ivanicki and Nešić and Vodnik expanded the general picture of the different drying stages as depicted in Figure 1 into a five stage model, where an extra (boiling) plateau in the droplet temperature appears when the droplet reaches its boiling point [8].

A study of temperature effects was performed by Lin and Gentry using an aluminum filament as SDD technique [20]. The general trend for the formation of particles was in good correlation with the above described situations for both high and low temperature regimes. For NaCl solutions, suspended droplets at low temperatures resulted in small and dense particles. For temperatures closer to the boiling point of the solvent, a more rapid initial droplet diameter reduction is observed, while in the later stages, volume expansion occurs due to significant crust formation and residual solvent evaporation from within the crust, which becomes more significant at higher concentrations. Volume expansion was even more pronounced for temperatures above the boiling point due to an earlier onset of crust formation and a resulting larger content of non-evaporated solvent inside the core, giving rise to large, inflated particles. For NH_4_Cl solutions, trends were similar to the NaCl solutions, however, a more hollow structure was obtained due to an elastic but less permeable shell formed during drying. Lin and Gentry specify that the degrees of thermal expansion and solvent vaporization were proportional to the drying temperature for both regions [20].

Another example of the impact of working either below or above the boiling point could be seen after crust formation in SDD experiments performed by Tran et al. with skim milk, lactose, whey protein, and a lactose–whey mixture in water, conducted with a thin polyamide wire at different concentrations [47,48]. At low temperatures (i.e., below 100 °C in the case of aqueous solutions), the evaporation rate decreased fast during the second drying stage due to the increased crust heat and mass transport resistances. The droplet diameter after crust formation remained nearly constant. Particles with a larger diameter (up to 6%) were obtained below the boiling point by increasing the temperature and thus Pe, due to earlier onset of crust formation at larger droplet sizes. The particle surface was in this case partially wrinkled but not collapsed, the internal structure contained small cavities and the crust was relatively thick. When applying temperatures above the boiling point of water, the evaporation was accelerated, the formation of a (thinner) crust took place even earlier and at larger droplet diameters, and a larger volume expansion during the inflation/deflation period led to a single big cavity [47]. The above described general temperature trends and dependencies are in line with studies on a broad selection of solutions: skimmed milk droplets on a glass filament, hydroxypropylated pea starch (HPS) on a polyamide wire, sucrose droplets on a glass filament, poly(lactide-co-glycolide) in ethyl acetate in an acoustic levitator, dextrin droplets in an acoustic levitator, and mannitol droplets on a glass filament [49,50,51,52,53,54].

##### Relative Humidity

The RH present during the droplet drying process affects both the solid layer formation and the temperature profiles in a droplet. RH is the ratio of the partial pressure of the solvent in the gas phase $p_{v,g}$ to the equilibrium vapor pressure of the solvent $p_{v,\;s}$. While the overall temperature of the drying gas $T_{gas}$ is generally affecting the drying rate, as discussed above, it is the RH that is determining how effective drying at a given $T_{gas}$ takes place. For 100% RH, no drying occurs, irrespective of $T_{gas}$ (and the droplet’s wet-bulb temperature $T_{wb}$ is the same as $T_{gas}$). However, with decreasing RH, $T_{wb}$ is lowered and the ΔT between a given $T_{gas}$ and the droplet’s $T_{wb}$ linearly increases (as can be obtained from psychrometric charts for a given solvent/gas combination). Since it is the ΔT dependent heat transfer that determines the evaporation rate, a lowering of RH can be directly linked to an increasing Pe number. In general, slower evaporation equals a decrease in mass transfer rate within a droplet, allowing more diffusion of solute(s) and delaying the skin formation. In this way, a more homogeneous and less porous final particle is obtained. Based upon these studies, it could be stated that RH and porosity change proportionally to each other, meaning that the higher the RH, the lower the porosity of the particle will be [27].

For example, Griesing et al. investigated the effect of variations in RH and solution composition with mannitol-water droplets in an acoustic levitator [25]. They confirmed that increasing the RH from 1% to 15% increased the droplet’s $T_{wb}$ and thus caused a decrease in Pe number. This delayed the solid layer formation of mannitol and decreased both the particle diameter and porosity [25]. Similarly, Grosshans et al. explored the influence of varying RH on the evaporation and particle formation by producing water-mannitol droplets in an acoustic levitator [45]. When applying a higher RH (i.e., 5% and 7.5%) and thus lower Pe, particles with a smooth surface were produced, while for a RH of only 1%, the particles tended to have slightly irregular shapes and the particle formation started earlier [45].

Another good example is provided by the studies of Schiffter and Lee, where experiments were conducted with mannitol, trehalose dihydrate, and bovine liver catalase in water in an acoustic levitator [55]. It was shown that a higher RH led to slower evaporation rates in the first, constant-rate drying stage, hence postponing the locking point of the crust, and to an extension of the second, falling-rate drying stage. This went along with the observation of a decreased particle radius for higher RH. The same trend was observed when investigating catalase droplets, although the produced particles differed in morphology due to the differences in the material properties. For a low RH, mannitol resulted in hollow particles with a highly spiked surface and catalase particles were characterized by a blowhole, but with a smooth surface [55]. These materials can be classified as crystalline and skin-forming, respectively, according to the classification of Walton and Mumford [56].

Hygroscopy, i.e., the ability of the solute to attract and hold water, should be briefly mentioned due to its connection to RH and influence on the drying kinetics [14]. While heat transfer was discussed so far as the limiting factor, lowering of the vapor pressure of the solvent attracted to the solute (relative to the RH) limits the evaporation process for hygroscopic materials. Since the driving force for evaporation is the difference between the reduced vapor pressure and the RH of the gas, an increasing hygroscopy acts similar to an increasing RH. As a result, hygroscopic compounds will delay skin formation and generate smaller particles. Lowering the vapor pressure below the RH of the gas due to hygroscopy causes a reversal of the drying process, and an uptake of solvent into the drop, presenting a limit for RH above which no particle formation is possible [57]. Gregson et al. investigated the effect of varying RH on the drying kinetics of NaCl droplets [57]. They showed that installing a RH of 45% enabled them to cause NaCl to effloresce. When increasing the RH above this value, deliquescence took place after the particle was already formed. Thus, only if the RH of the environment was below this limit could particles be obtained [57].

##### Droplet Size

The variety of droplet sizes that can be produced is large and depends on the utilized droplet generator, as introduced in Section 2.1 [15]. If a constant evaporation rate is assumed, the drying time is proportional to the square of the initial droplet diameter since the surface area decreases linearly over time [3]. In general, for the same total fluid volume to be evaporated, larger droplets will take longer to solidify at similar process conditions due to their lower surface-to-volume ratio compared to smaller droplets. To exemplify, Sedelmayer et al. observed a 2.5 times faster drying rate for mannitol droplets of 350 µm in comparison to 600 µm [58,59]. In line with the Pe number discussion, the faster evaporation led to earlier crust formation for smaller droplets, forcing the residual solvent to evaporate through pores. This phenomenon gave rise to an increased porosity or even the creation of hollow and collapsed particles [59]. The initial droplet size determines to a large extent the final particle size (see Equation (1)).

Furthermore, temperature gradients and inner mass flows can be significant for larger droplets. The Biot number (Bi) is often used in heat transfer calculations to determine whether temperature gradients are of importance:

(4) $$\mathrm{Bi}=\frac{hd_{d}}{k}$$

where h [W m^−2^ K^−1^] is the droplet’s heat transfer coefficient and k [W m^−1^ K^−1^] is the thermal conductivity. For Bi≪1, the droplet temperature can be assumed to be uniform for the complete particle. This is generally the case for the small droplets generated by the pharmaceutical processes discussed in Section 3.

Additionally, droplet shape destabilization can occur depending on the inertial forces on the droplet during the process, which can deform the droplets to be more flat, and the interfacial tension of the droplet, which generates spherical shapes due to surface energy minimization. The Bond number (Bo) is often utilized to give a quantitative measure of this balance:

(5) $$\mathrm{Bo}={\left(\frac{r_{d}}{\lambda_{c}}\right)}^{2}$$

where $r_{d}$ is the droplet radius [m] and the $\lambda_{c}$ capillary length, which is defined as:

(6) $$\lambda_{c}=\sqrt{\frac{\gamma }{\Delta \rho g}}$$

where g is the gravitational acceleration [m s^−2^], Δρ is the difference in density of the droplet and the surrounding medium [kg m^−1^], and γ is the surface tension of the fluid interface [N m^−1^] [60,61]. For larger Bo numbers, the droplet shape becomes more easily affected by inertial forces. Hence, droplet shape changes are not only more evident for larger droplets, but also for larger density differences Δρ and lower interfacial tensions γ [62].

#### 2.2.2. Formulation Parameters

##### Chemical Composition of Solute

A strict classification of parameters influencing the drying of a solution droplet based solely on the chemical nature of its compounds is hard to make. Instead, a more general classification can be made based on the final particle morphologies [56,63]. According to Walton and Mumford, three categories can be distinguished, namely skin-like (e.g., skim milk, gelatin), crystalline (e.g., sodium chloride, sodium carbonate), and agglomerate (e.g., silica) materials [56,63]. A particle can be considered skin-like when the solid phase is continuous and is comprised of polymers or sub-microcrystalline materials (acts as a continuous phase due to a sub-micron structure). In crystalline materials, the final particle consists of macro crystals bounded by a continuous microcrystalline phase, while the agglomerate material is composed of grains connected by sub-micron particles of the same material. Organic compounds mostly fit in the skin-like category, while inorganic compounds can be associated with both crystalline and agglomerate categories. It should be mentioned that there are exceptions to this categorizing system. An example of this unpredictable behavior is sodium silicates, which are inorganic salts and should be exhibiting crystalline behavior, but instead have skin-forming properties [56].

##### Solute Concentration

A high solute concentration is often desired in pharmaceutical processes like spray drying to ensure high solid loadings and less wastage of (expensive) solvents [15]. The solute concentration has an important influence on the final particle size and particle morphology. Larger particles are formed when more solute content is present in the initial solution (see Equation (1)). However, the solute concentration only affects particle size in a substantial way when dilute solutions are used, as will be discussed in the spray drying part. Higher solute concentrations also result in higher effective viscosities η and thus in lower diffusion coefficients, as indicated in the Stokes-Einstein equation (i.e., Equation (3)). Hence, larger values for the Pe number are generally observed for increasing concentrations, promoting more rapid skin formation and thus even larger particles than predicted by Equation (1).

Okuzono et al. quantified the skin/crust formation dependency on the solute concentration based on transport and mass balance, however, assuming the diffusion coefficient to be constant during the period of drying until the onset of skin formation. In that case, skin formation is only observed above the critical Pe number:

(7) $$\mathrm{Pe}>\frac{\phi_{c}-\phi_{0}}{\left(1-\phi_{0}\right)\phi_{0}}$$

where $\phi_{c}$ is a critical solute volume fraction at which a skin forms, and $\phi_{0}$ is the initial solute volume fraction [64]. The critical volume fraction $\phi_{c}$ and thus the actual onset of skin formation strongly depends on the type of solute. Okuzuno et al. focused on polymer solutions that form gel-like skins, for which they assumed $\phi_{c}$ to be equal to 0.2.

Grosshans et al. performed SDD experiments with an acoustic levitator in order to observe the effects of varying concentrations of mannitol in water on particle size and crust formation [45]. When a low solid content loading was applied (i.e., 5 wt%), a delayed formation of a thinner crust and a smaller particle diameter was observed, in comparison to the higher concentrations [45]. Fu et al. studied shrinkage kinetics of a droplet on a glass filament and its relation to the concentration of dissolved compounds (e.g., sucrose, lactose, mannitol) [54]. Less shrinkage of the droplets was observed with increasing concentration (from 10 wt% to 50 wt%), irrespective of the dissolved compound, and larger particles were formed [54].

It is also reported that concentration affects the surface morphology of the final particle, although this effect is not unambiguously defined and strongly depends on the experimental drying conditions. Sedelmayer et al. carried out acoustic levitation droplet drying experiments with (NH_4_)_2_SO_4_ and mannitol and stated that the surface roughness has a linear dependence on the solute fraction, namely the higher the concentration of the dissolved solid, the rougher the surface [58,59]. In the research of Schuytser et al., in contrast, smoother surfaces were obtained for higher concentrations [17].

##### Surface-Active Components

Surface-active components are used to change and control the surface tension of droplets. Surface tension forces stabilize droplets and varying the droplet’s interface with a surfactant too much can imply skin instabilities during drying [65]. Components that alter the surface tension can either decrease (e.g., a polymer) or increase (e.g., a salt) the surface tension [61]. Surface tension gradients in the droplet’s interface cause Marangoni flows, which are interfacial flows that try to re-equilibrate the concentration of solutes at the air–liquid interface (and thus the interfacial tension). The interfacial flow drags the bulk solution and therefore results in solute circulation within the droplet. Surfactants reduce this internal flow by adhering to and occupying the droplet interface and by this reducing surface tension fluctuations. Their adherence to the interface changes the composition of the particle crust, thereby affecting the stability to withstand internal stresses and thus the final morphology [14]. Different morphologies can also be obtained when several surfactants with differing solubilities are applied, however solubility effects are discussed in more detail in Section 2.2.3. In the review of Sadek et al. it was mentioned that more spherical and more hollow particles are formed for surfactants with higher surface activities [14]. This was attributed to enhanced crust formation on the surface, which promoted the formation of larger particles [14]. Adler et al. have shown that small surfactant molecules, like Tween 80, can diffuse more easily than larger molecules enabling the smaller surfactants to accumulate at the interface of a droplet much faster [66]. The importance of the molecule size of surfactants is emphasized by acoustic levitation SDD experiments of Nuzzo et al., where the influence of different surface-active polymers on the particle morphology was studied [67]. Bovine serum albumin (BSA), HPMC or poloxamer were dissolved in water together with α-lactose monohydrate. The surface of the resulting BSA/lactose particles was wrinkled; however, the morphology still resembled a spherical form. In contrast, the HPMC/lactose particles were almost in a collapsed state, characterized by multiple indentations. This difference was due to the slow adsorption rate and lower dilatational surface elasticity for BSA in comparison to HPMC. Spherical particles with a smooth surface were obtained for the poloxamer/lactose combination because the poloxamer molecules are smaller than BSA and HPMC and entail the formation of a flexible and soft thin layer. The change in the surface morphology is attributed to the molecular weight related low dilatational surface elasticity of poloxamer which allowed the droplet surface to contract during drying without deformations [67].

#### 2.2.3. Component Characteristics

##### Molecular Weight and Size

The compounds generally applied in the pharmaceutical industry for particle manufacturing cover a wide range of molecular weights, from low molecular weight compounds such as salts, additives, and active pharmaceutical ingredients to higher molecular weight polymers and proteins, often combined in formulations. According to Walton and Mumford, lower molecular weight substances are generally forming crystals at their saturation points while high molecular weight substances are skin-forming materials upon drying [56]. The molecular weight of a solute is related to its hydrodynamic radius $R_{h}$, that enters the diffusion coefficient $D_{i}$ according to Equation (3). Reducing the size of the components will allow better diffusion due to increased movement ability [9].

The $R_{h\;}$of a solute is related to the amount of building blocks out of which it consists, as well as their spatial distribution in a solvent. For polymer molecules, the polymer chain conformation and thus $R_{h}$ can greatly differ in different solvents. In favorable or ‘good’ solvent systems, the polymer chain will be in a more extended form while in ‘bad’ solvents, the polymer chain is in a more coiled state. The intrinsic viscosity [η] gives an indication of this solvent quality, and moreover of the potential of the polymer to increase the effective viscosity η of the solution at a given concentration. Similar to the previously discussed effect of higher solute concentrations, also the expansion of the polymer and thus the increasing $R_{h}$ increases the effective viscosity η, and this already at relatively low mass concentrations. Both the increased $R_{h}$ and η, that lead to a lower diffusion coefficient and hence a higher Pe number (see Equations (2) and (3)), result in easier skin formation for higher molecular weight compounds, while lower molecular weight compounds are more likely to diffuse and be homogeneously distributed in the final particle.

An increased diffusivity can be obtained by reducing the size of the components. During evaporation, substances with a higher diffusion coefficient move from the air–liquid interface to the center, whilst substances with a lower diffusion coefficient mostly stay at the interface [67]. It becomes less trivial when similar diffusivities are present or when multicomponent systems are used, for example with the addition of surfactants or extra solutes [65,66,68]. A solution that consists of two components of similar size can result in a particle with well-distributed components because of similar diffusion velocity [2]. If components with different sizes are dissolved, the situation is different. In this case, the component with lower diffusivity (i.e., the larger component, as can be derived from Equation (3)) occupies the surface, while the center mainly consists of the smaller, high diffusivity substance [69]. Regarding the final particle morphology, an early skin formation can be observed when the dissolved solids are characterized by a low diffusivity. As mentioned before, this early skin formation can result in hollow, collapsed, porous, or wrinkled particles, depending on the mechanical strength of the crust and thus on the properties of the solutes. For compounds with a high diffusion coefficient, more rigid, dense, stable, and compact particles are obtained since the solute particles are able to diffuse inwards within the droplet, thereby keeping the component distribution homogeneous [9,14,61].

##### Solubility

While it was generally discussed above that for larger Pe values eventually a skin/crust is formed, the exact onset point depends on the type of solute. This does not only reflect in the solute dependent critical onset concentrations of Equation (7), but is also influenced by the solubility of solutes, the solubility evolution during solvent evaporation (i.e., how and when solubility limits are reached) and the specific locking or crust forming mechanism. For solutes with a high solubility, it takes longer to reach the saturation limit at the surface and to precipitate at the droplet’s interface. The formation of a solid layer thus happens only at later stages and allows for the crust to solidify homogeneously due to the fact that most of the solvent is already evaporated before the locking point. A thicker and more stable crust with minimal deformations is obtained in this case. On the other hand, a thin and brittle shell is expected for solutes with a low solubility in the solvent used, since the saturation limit is reached earlier [3,70]. In principle, when the solutes have a vast difference in solubilities, the first to occupy and dominate the droplet surface are ones with a lower solubility, which are usually also the larger and slower diffusing molecules that initiate crust formation [71].

This has been illustrated in studies by Lin and Gentry, where small and dense particles were produced for substances with a high solubility, and larger particles with wrinkling and deformations were obtained for low solubility substances [20]. Indeed, the latter was attributed to the lower strength of the thinner crusts [20]. Osman et al. observed more irregularities and deformations in the final particle morphologies for surfactants in water solutions containing a higher molecular weight alkyl chain and they attributed this phenomenon to their lower solubility compared to surfactants with lower molecular weight alkyl chains [65]. During the drying process, the surfactant monolayer at the surface will compress due to surface area reduction, leading to a pressure on the monolayer. This pressure can be relieved by the migration of some surfactant molecules at the interface to the aqueous bulk phase. When the solubility of the surfactant is low, this phenomenon is less pronounced due to the inability of the surfactant to dissolve into the bulk phase and the monolayer persists for a longer time until buckling and deformation occurs [65]. Interesting morphology evolutions due to varying solubilities were also presented by Wulsten et al., who performed acoustic levitation SDD experiments with drug–polymer solutions [29]. Itraconazole was dissolved in DCM/ethanol together with PVP-VA 64 or HPMC. PVP-VA 64 has a higher solubility in DCM/ethanol than HPMC, thus postponing the locking point. In contrast, when HPMC was used, an earlier precipitation and solid layer formation took place [29].

##### Solvent Volatility

One of the most important properties of solvents affecting the evaporation rate and final particle morphology is the solvent volatility [14]. Evaporation is controlled by the heat transfer to the droplet surface, and therefore by the temperature difference between the gas $T_{gas}$ (set as a process parameter) and the wet-bulb temperature $T_{wb}$ of the droplet. The wet-bulb temperature $T_{wb}$ in turn is set by the RH of the specific solvent. Highly volatile solvents (i.e., low boiling point $T_{b}$ or high vapor pressure $p_{v,s}$) will therefore exhibit higher evaporation rates and Pe numbers at a given temperature and partial pressure facilitating the formation of hollow or collapsed particles. Low evaporation rates and volatilities on the other hand assist in the production of dense particles [20,72]. For a system with multiple solvents, the solvents with higher volatility will evaporate faster, causing the Pe numbers to decrease during the droplet drying process.

This has been illustrated by particle formation from the evaporation of a mixture of higher volatile ethanol and water, as performed by Gregson et al., and from the evaporation of a mixture of ethanol and higher volatile DCM, as reported by Wulsten et al. [29,73]. The preferential evaporation at the early stages was for ethanol and DCM, respectively.

### 2.3. Experimental Characterization of the Single Droplet Drying Process

To understand and verify the influence of parameters that play a key role in the drying process of single droplets, precise experimental observation techniques and measurements are required. This is of utmost importance to gain insight in the link between the droplet properties, the parameters that influence them and the final particle morphology, but also to provide a sound parameter base for subsequent modeling of the involved physical phenomena. As became clear from the previous section, there are lots of parameters that can influence droplet drying, although not all of them can be monitored during the drying process. Observable parameters that impact droplet drying are droplet mass, diameter or volume, temperature, component distribution and concentrations, velocity, possible physical deformations, and the vapor concentration surrounding the drying droplet [14]. Several experimental techniques to monitor these parameters are based on optical measurements and exploit the partial dependence of light interaction on the temperature and chemical composition of the matter [74]. Hence, these techniques employ electromagnetic waves emitted from the (dissolved) components to investigate their distribution in the droplet and temperature gradients inside the droplet during drying. Next to these optical measurement techniques, methods relying on other principles can be applied as well. In Table 2, several techniques are grouped by the different parameters they can measure during the drying process. In the upcoming section, some of them are shortly described and examples of their potential application to the drying process are reported.

#### 2.3.1. Droplet Characterization

Fluorescence- and phosphorescence-based methods can be employed to determine the temperature and, to a lesser extent, composition of drying droplets. These photoluminescence processes are the result of spontaneous relaxation of molecules after excitation, which can be achieved by applying laser light. These techniques are known as laser-induced fluorescence (LIF) or phosphorescence. In case of LIF, the fluorescent component can be an organic dye, a thermographic phosphor or fluorescent molecules naturally present in the droplet [74,75,76]. To exemplify the latter, Maqua et al. evaluated the composition of binary droplets made of ethanol and acetone by exploiting that acetone could absorb ultraviolet (UV) light and thus monitoring the intensity of its emitted light, which was proportional to the acetone concentration in the droplet [77]. Similarly, Shringi et al. visualized the acetone concentration fields inside single droplets of both pure acetone and mixtures of acetone and either methanol or propanol [78]. Additionally, Mercier et al. investigated acetone droplets using planar laser-induced fluorescence (PLIF) on hydroxyl radicals and acetone itself and thereby visualized the spatial distribution of the vapor and the structure of the flame around the droplet [79]. If there is no fluorescent molecule naturally present in the formulation, a fluorescent dye can be introduced. It is worth to mention that, since only a small amount of these dyes (µg/L) is necessary to generate a measurable fluorescence signal, their presence generally does not affect the fluid properties such as surface tension, viscosity or thermal conductivity. Some organic dyes, like Rhodamine B, have the benefit that their high fluorescent yield is strongly temperature dependent and are therefore widely applied to measure the temperature using the two-color laser-induced fluorescence (2cLIF) thermometry technique [74]. This technique uses the ratio of the signals detected on two well-separated spectral bands, having a different temperature sensitivity, to measure the liquid temperature excluding the effects of unknown properties, such as laser intensity, dye concentration and dimensions of the probe. For instance, Lavieille et al. added Rhodamine B as a fluorescent dye to a stream of monodisperse ethanol droplets to exploit the temperature dependence of its fluorescence yield [80,81]. They further combined temperature measurements with velocity measurements obtained via phase doppler anemometry (PDA), which enabled them to study the droplet dynamics and thermal behavior simultaneously [81]. Next to spatially averaged temperature measurements, laser-induced fluorescence can be used to measure the local temperatures inside droplets providing that the measurement probe volume is smaller than the size of the droplet [74]. In this way, Castanet et al. characterized the temperature fields of diverse fuel droplets (acetone, ethanol, pentanone, heptane, decane, dodecane) undergoing evaporation and used them to estimate the sliding velocity at the liquid–gas interface [82]. 2cLIF thermometry can also be applied to characterize the temperature of multicomponent droplets. However, distinguishing between temperature and composition effects is not straightforward. Maqua et al. were able to measure the temperature of binary droplets made of ethanol and acetone by adding Rhodamine B and measuring its emission in three spectral bands [83]. The use of a third band was essential as it had a different sensitivity to both temperature and ethanol volume fraction, which made it possible to separate the effect of composition and temperature on the emitted light [83]. A more advanced fluorescence-based method is laser-induced exciplex fluorescence (LIEF). Here, two organic tracers are added to a non-fluorescent liquid, from which one of the tracers (the monomer) is brought to an excited state by UV light illumination and interacts with the other tracer molecule, thereby creating an exciplex. The production of an exciplex can only take place when the monomer is in its excited state, which is much more likely to happen in the liquid phase rather than in the gas phase, because of the higher frequency of molecular collisions in the liquid. LIEF is mostly applied to distinguish between the liquid and gaseous phase and to quantify the amount of liquid still present [74,84]. Two-color LIEF was for example employed by Zhang et al. to study the liquid/vapor mass distribution simultaneously with the droplet temperature in a monodisperse stream of n-hexane droplets [85].

All the aforementioned fluorescence-based techniques rely on the intensity of the fluorescent signal. Next to this, determination of the fluorescence lifetime enables temperature and concentration determination as well, as it is an intrinsic property of a fluorescent species that is solely dependent on its chemical environment [74]. Lifetime measurements were implemented by Zeng et al. to quantify the mixing dynamics in microfluidic water droplets by the use of two fluorescent dyes (i.e., Lucifer Yellow and Alexa 430) [86].

Phosphorescence can be achieved by adding thermographic phosphors to the formulation, enabling temperature investigations as well. These inorganic phosphors are made up of a host material, such as metallic oxide, and an activator, responsible for light emission when exposed to radiation. When added to the droplets of interest, the thermal dependence of their light emission can be exploited to evaluate the thermal behavior of the droplet [74]. Omrane et al. seeded droplets with thermographic phosphors and tracked their time decay to determine the temperature of droplets made up of water and acetone mixtures [87]. Further, they extended these measurements to 2D temperature fields using a high-speed camera [88].

**Figure 5:**
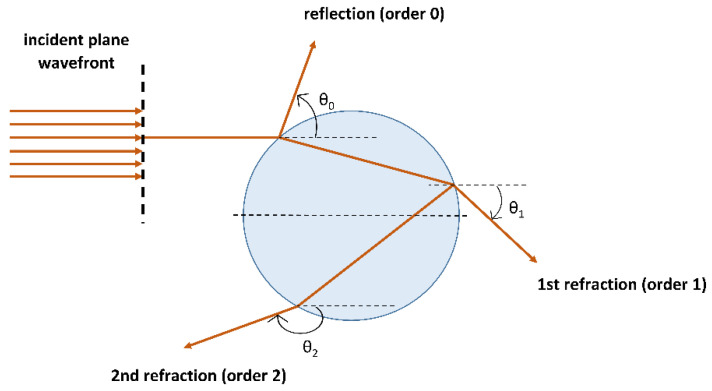
Light paths inside a droplet, illuminated by a plane wave (modified from Lemoine et al. [74]).

Different droplet characteristics, such as their size and their composition, can be measured using optical techniques based on elastic scattering (i.e., rainbow refractometry and PDA) or inelastic scattering (i.e., Raman spectroscopy). The rainbow refractometry technique analyzes the interference patterns created by the interaction of light with a spherical droplet and determines both the droplet diameter and the refractive index (RI). From the RI, information about the temperature and chemical composition can, in some cases, be deducted [74]. This interference pattern is generated by the light rays which experience three interactions with the droplet (order 2 rays in Figure 5) and are parallel to each other. 

The produced interference pattern is called primary rainbow and its angular position and fringe spacing depend on the droplet size and composition. To exemplify, Promvongsa et al. applied rainbow refractometry to determine the diameter and refractive index of binary droplets made of water and ethanol [89]. This technique enabled to measure the composition changes of the drying droplet (i.e., the volume fraction of ethanol) and the evaporation rate based on the evolution of the droplet diameter. The capability of rainbow refractometry to determine the droplet temperature becomes clear from a study performed by Li et al., where they explored the effect of varying the droplet diameter on the rainbow positions and temperature distributions of burning n-alkane droplets [90]. Both droplet shape and RI uniformity affect the primary rainbow. In particular, the impact of radial RI variation inside evaporating droplets on the rainbow refractometry technique has also been investigated by several authors. For instance, Vetrano et al. formulated an analytical model for burning droplets that exhibit internal RI gradients [91]. In addition, the phase of the primary rainbow is also sensitive to changes in droplet size and RI. When information is deducted from the phase of the primary rainbow, the technique is named phase rainbow refractometry and has been used by Wu et al. to measure the temperature, size, and evaporation rate of fuel droplets simultaneously [92,93]. They were able to detect nanoscale size changes in a direct way, rather than by tracking diameter changes in time. Rainbow refractometry has also been combined with other light scattering techniques, such as a phase doppler analyzer, sometimes also referred to as a phase doppler anemometer [94]. A PDA measures the size and the velocity of droplets while they pass through an interference fringe pattern generated by a laser source. As the droplet passes through the fringes, it generates a light scattering wave, of which the fluctuations are proportional to its velocity. Moreover, by measuring the light scattered in three specific spatial locations, the size of the droplet is obtained as well. Zhao et al. employed both a rainbow refractometer and a PDA to simultaneously study the temperature, velocity, and size of a stream of monodisperse droplets during evaporation [95]. They explored the evolutions of these parameters in multicomponent droplets existing out of ethanol and water, which allowed them to conclude that the mass fraction of the mixture remained constant during the drying process [95]. In the aforementioned examples, single spherical droplets or monodisperse droplet streams were studied; hence the standard rainbow refractometry technique was applied. Rainbow refractometry extended to multiple droplets is called global rainbow refractometry (or thermometry) and is widely used in spray diagnostics (see Section 3.4) [96,97].

Alternatively, Raman scattering can also disclose evolutions in composition, size and to some extent temperature of droplets undergoing evaporation [98]. Both spontaneous and stimulated Raman spectroscopy can serve this purpose. Vehring et al. established the limits of spontaneous Raman spectroscopy with respect to detection limits and size dependency, where the detection limit was compound dependent, and they were able to characterize droplets as small as 3 µm [99]. Additionally, Moritz et al. tested the capability of this light scattering technique to determine the composition of a droplet [100]. They pointed out that the intensity of Raman scattering in spherical microdroplets was also dependent on the spatial distribution of the molecules inside the droplet due to the formation of morphology dependent resonances (MDRs) [100]. Although Raman spectroscopy is mostly used to probe the composition of the droplet, it has also been applied to evaluate the temperature. Suzuki et al. successfully used the combination of an optical trap with Raman spectroscopy to capture the thermodynamic behavior of single droplets of water in a supercooled state [101]. Additionally, Heinisch et al. investigated the temperature evolutions in single water droplets that evaporated while injecting nitrogen gas [102]. Spontaneous Raman spectroscopy is limited by a very weak signal, hence powerful and highly focused laser beams are indispensable to obtain a signal that can be detected. As an alternative, the stimulated Raman effect, that relies on the amplification of the incident wave and Raman signal in whispering gallery modes (WGMs), can be employed to obtain a higher intensity of the signal [98]. On top of that, the sensitivity to the Raman signal for variations in composition increases exponentially. A possible way to exploit the stimulated Raman effect is by using cavity-enhanced Raman scattering (CERS), also referred to as surface-enhanced Raman scattering (SERS) when applied to droplets. This technique can be implemented to evaluate composition changes at the surface of the droplet [103]. For instance, in the first study in which the drying process of volatile components was evaluated in the millisecond time scale, Hopkins et al. applied both CERS and LIF to investigate the size, composition and temperature of binary droplets existing out of water and ethanol [104]. Here, CERS allowed to measure the concentration of ethanol by capturing the changes in composition near the surface of the droplet [104]. Kihara et al. also explored the mass transfer phenomena at the surface of the droplet using CERS [105]. More specifically, they aimed to gain a deeper understanding of the surface kinetics by investigating the surface concentration of alcohol molecules at the surface of water droplets. It was found that a decrease in alcohol concentration was followed by an increase in concentration, which was explained by evaporation and a subsequent adsorption of the molecules at the surface [105].

An ulterior technique, the thermochromic liquid crystal infrared thermometry, uses thermochromic liquid crystals to measure temperature of the droplet. The technique is based on the fact that cholesteric and chiral nematic liquid crystals can reflect light in a specific wavelength range as a function of temperature [74]. Richards and Richards suspended microencapsulated beads of these thermochromic liquid crystals in water droplets to visualize the transient temperature fields in the droplets during the cooling process [106].

A completely different principle that can also help to gain insight in the drying process is nuclear magnetic resonance (NMR). Griffith et al. used NMR to determine the water content in a single drying droplet of a surfactant-based mixture, used for the production of detergent powders [107]. This allowed the development of a theoretical model using a diffusion coefficient that was dependent on the local water content in the droplet [107].

#### 2.3.2. Particle Characterization

Next to tracking the droplet to particle formation, the particle morphology after drying, its size, and physical structure can also be evaluated. As the focus of this work is on the drying process of droplets, only a short overview of particle characterization techniques is reported. Nevertheless, these techniques are indispensable to gain a deeper understanding of how and to which extent the drying process results in a certain morphology and has an influence on the particle properties and performance.

The most commonly used microscopy techniques to explore the morphology of the dried particles are scanning electron microscopy (SEM) and (cryo-) transmission electron microscopy ((cryo-)TEM) [15]. Fu et al. combined SEM and X-ray diffraction (XRD) to characterize the morphology and crystallinity of single lactose particles [22]. Similarly, Wulsten et al. applied SEM to characterize the morphology of particles obtained after drying of itraconazole and PVP-VA 64/HPMC dissolved in ethanol and DCM mixtures [29]. In this way, the influence of the polymer on the morphology was demonstrated [29]. On top of SEM, confocal laser scanning microscopy (CLSM) can be employed to gain insight in the distribution of the components. For instance, Al Zaitone and Lamprecht applied this combination of techniques to explore the effect of varying drying process parameters (temperature, concentration and droplet size) on the morphology of poly(lactide-co-glycolide)/ethyl acetate particles [51]. The abovementioned microscopy techniques also provide information about the size of the individual particles by performing digital image analysis [15].

The physical structure of a particle can be explored using XRD, from which the polymorphic form and amount of crystalline material can be deducted. The added value of XRD for understanding the drying process already became clear from the abovementioned study performed by Fu et al. [22]. It was also applied by Foerster et al., who used flash-freezing of milk droplets to stop the drying process at any given time and subsequently investigated the surface composition with XRD and the component distribution using CLSM [1,108].

### 2.4. Models for Single Droplet Drying

Since the drying of droplets, containing dissolved solids, to create solid particles is applied in many different industries and is subject of extensive research, theoretical studies of the drying behavior of single droplets have gained a lot of interest in the last decades. A theoretical framework or constitutive model for the drying behavior or process of single droplets allows for correct predictions of the final product characteristics and is often necessary to be incorporated in computational fluid dynamics (CFD) modeling for an accurate representation of the formation processes. There are multiple approaches to model the drying behavior of droplets of which three will be described in more detail in the following [109,110]. The first approach is deterministic analytical modeling, which describes droplet drying by expressing the transport phenomena occurring during the lifetime of the droplet. The process is described by a set of computationally expensive coupled differential equations for the heat and mass transfer. The second approach is the characteristic drying rate curve (CDRC) concept, which is quite often applied as a semi-empirical alternative to decrease the computational difficulty of solving the differential equations, while in many cases still allowing for good description of the process. The third approach, called the reaction engineering approach (REA), empirically describes the droplet drying behavior as an activation process to allow for fast computational processing.

#### 2.4.1. Deterministic Analytical Models

For this approach, the transport phenomena occurring during the drying of the droplet are described by a set of coupled differential equations with corresponding initial and boundary conditions. The early fundamental work that combined theoretical modeling and experimental observations of both pure liquid droplets and droplets that contained dissolved and suspended solids was published by Ranz and Marshall [111]. Charlesworth et al. continued on that work, focusing on droplets containing dissolved solids, and identified different drying periods with different evaporation rates depending on crust formation [44]. In most deterministic models, the drying process is split up into the different stages discussed in Figure 1 in the introduction, where crust formation happens when a critical surface concentration is reached during the lifetime of the droplet [7,8,112,113]. These models generally apply a distributed-parameter approach, where different sections of the drying droplet can exhibit distinct physical properties, and where parameters are spatially distributed. In contrast to this, the CDRC approach and the REA, which will be discussed later in this chapter, use a simplified lumped-parameter approach that assumes a single homogeneous droplet with uniform properties. In most of the deterministic analytical models, the droplets are assumed to be spherical and result in single particle of the dissolved solute, hence coalescence or droplet division are not considered.

A detailed description of the specific transport equations for the deterministic approach is, however, beyond the scope of this review. This paragraph gives therefore only an overview about the relevant studies employing this concept. Mezhericher et al. have composed a comprehensive review on the literature of theoretical models and have developed their own advanced droplet drying model, described in several publications by the same authors [110,114,115]. Here, they make a distinction between the first constant drying rate region and the second falling-rate region, where a crust is developing at the surface of the droplet surrounding a wet core. Gac et al. have modified the previous model to allow for investigation of multi-component solutions where segregation of the different components can occur [116]. They concluded that segregation is prominent if the components have significantly different solubility or diffusion coefficients or if the components both have relatively low solubility but enough time passes between onset of crust formation and complete spherical porous crust formation [116]. Similarly, Wang et al. developed a distributed-parameter model by dividing the particle into nine distinct layers with the goal to understand and predict the segregation in multi-component systems [117]. Other studies where component segregation and surface composition is investigated and modeled include the work of Chen et al., who derived more simple algebraic equations from the diffusion-convection partial differential equations in a continuum approach, and Xiao et al., who extended the previous model by adding a packing model on molecular level and in a later publication adjusted the methodology to be applicable for droplets with a certain size-distribution [118,119,120]. Parienta et al. examined the evaporation of exhaled respiratory droplets and proposed a mathematical model based on the multiple shell concept [121]. Seydel et al. locally modeled the mass and energy transfer inside droplets during solidification to predict final product quality [122]. The goal of the expanded model by Grasmeijer et al. was to create a model that can predict the drying kinetics of the droplets during all stages in one continuous model, allowing for an insight in the distribution of different components in the final particle [123]. The focus of their investigation was on a sugar/protein system, where it became clear that the relatively large protein molecules accumulate more at the surface of the droplet due to its lower diffusion coefficient compared to the smaller sugar molecules [123]. Werner et al. incorporated the morphological development of the crust to more accurately predict the later stages in the drying process, and extended their previously published effective diffusion model [124]. They propose that the mechanical stress present in the crust of the droplet is related to its temperature difference with the surface glass transition temperature, $\left(T-T_{g}\right)$ [124]. A model based on the work of Gopireddy et al. is modified to be applicable for drying conditions at elevated temperatures above the boiling point of the liquid in the droplet by Grosshans et al. [46,125]. Based on the analytical solutions to the heat and mass transfer equations during droplet evaporation, Sazhin et al. developed their new model and applied it to spray dried chitosan particles [126]. Sadafi et al. investigated the drying of saline water droplets and modified previous models by adding an additional stage in the drying process to take into account the often inhomogeneous crust formation over the surface of the droplet [127,128]. They added a time-dependent weighting factor to represent the gradual crust formation compared to the often-assumed instantaneous crust formation at a certain critical surface concentration in previous models.

Since the numerical solutions of these models are relatively complex and require a lot of computational effort, two alternative (semi-)empirical approaches will be discussed in more detail in the following paragraphs. Furthermore, the numerical solutions generally require a priori knowledge of a multitude of different coefficients and parameters for the correct evaluation of the equations. These are often not readily available and/or require a difficult experimental determination. Therefore, both the CDRC approach and the REA simplify the drying process while still providing good prediction of the droplet drying kinetics. The obtained drying kinetics for single droplets by these methods can be applied in more complex CFD simulations of drying processes like spray drying.

#### 2.4.2. The Characteristic Drying Rate Curve (CDRC) Approach

The semi-empirical CDRC approach assumes that, at any period during the drying of the droplet, a characteristic drying rate N is present which can be related to the free unhindered evaporation of a pure liquid droplet in the first stage of Figure 1, $N_{c}$ [129]. This first stage drying rate $N_{c}$ is assumed to be constant and the liquid content loss a linear function of time, limited by the heat transfer to the droplet surface. A non-linear liquid loss of the droplets eventually occurs when the dissolved solid content generates concentration gradients in the droplet, hence causing changes in surface composition and thus changes in heat and mass transfer coefficients. Therefore, the relative drying rate ξ is defined as:

(8) $$\xi =\frac{N}{N_{c}}$$

The characteristic liquid content Φ is defined as:

(9) $$\Phi =\frac{X-X_{eq}}{X_{cr}-X_{eq}}$$

where X is the liquid content at any moment on a dry basis (kg liquid kg^−1^ dry solid), $X_{cr}$ the critical liquid content where the transition from the first, constant rate regime to the second, falling drying rate regime takes place, and $X_{eq}$ is the liquid content at equilibrium at the end of the drying process, which depends on the environmental conditions. The critical liquid content $X_{cr}$ is experimentally determined and assumed to be constant during drying for a certain system and drying condition. Still, $X_{cr}$ is dependent on the applied drying conditions, so this variable needs to be determined whenever conditions change. The actual drying rate at any given moment can then be expressed as a function of the liquid content present in the droplet:

(10) $$N=\xi N_{c}\;\xi =f\left(\Phi \right)\;$$

where for unhindered evaporation (Φ≥1) ξ=1 and for hindered evaporation (0<Φ<1) 0<ξ<1.

**Figure 6:**
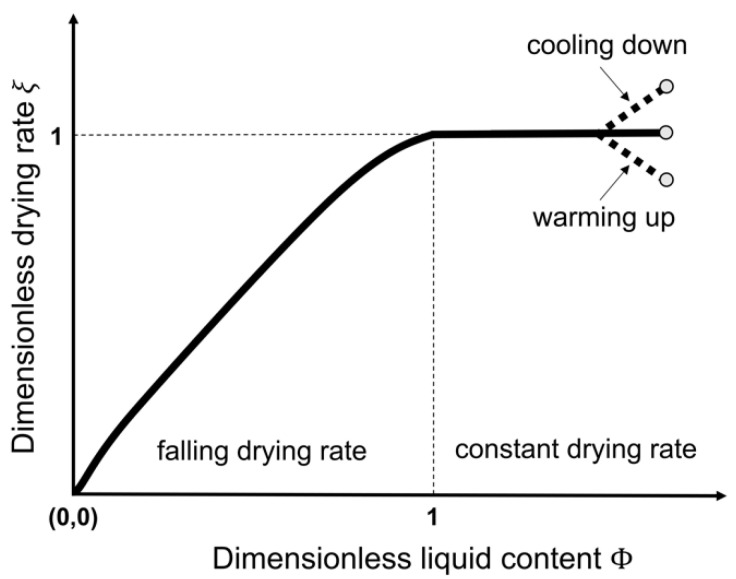
The evolution of the dimensionless drying rate depending on the dimensionless liquid content to give a characteristic drying curve (modified from Chen and van Meel [109,130]).

A visual interpretation of the relation between ξ and Φ is given in Figure 6 (which is a non-dimensional representation for the drying regimes of Figure 1), where also the possibility of an initial period of warming up or cooling down of the droplet to the environmental conditions is indicated [109,130]. The functional form of the drying curve is assumed to have a unique shape, solely depending on the material and characteristic liquid content Φ, so unrelated to any external process conditions. The simplest expression of the drying rate at any moment is:

(11) $$N=\xi \left[\beta_{1}\left(p_{v,s}-p_{v,g}\right)\right]$$

where $p_{v,s}\;$and $p_{v,g}$ are the vapor pressure at the fully wetted surface (fully saturated: $p_{v,s}=p_{v,sat}$) and in the bulk gas of the environment, respectively, and the constant $\beta_{1}$ is the external mass transfer coefficient. Although this representation of the drying rate (i.e., in terms of difference in vapor pressure $p_{v,s}-p_{v,g}$) is mostly found in literature, it should be noted that it is the heat transfer and thus the temperature difference (or the wet-bulb depression $\Delta T=\left(T_{gas}-T_{wb}\right)$ as mentioned in Section 2.2.1) that limits and controls the transport phenomena. Hence, Equation (11) can be rewritten in terms of the wet-bulb depression $\left(T_{gas}-T_{wb}\right)$:

(12) $$N=\xi \left[\beta_{2}\left(T_{gas}-T_{wb}\right)\right]$$

The functional form of the drying curve and in particular the coefficients are determined from the fitting of actual drying rate curves from experimental observations of single droplet drying under certain processing conditions.

One of the main advantages of this approach is its theoretical simplicity and very limited computational requirements. Keey et al. presented this approach for the drying of moisture from a slab of infinite extent and Langrish et al. applied it to the drying of milk powder from droplets, analyzing data from previously published work on this system [129,131]. Experimental studies have shown that this technique performs better for systems with small particle sizes [110,132]. In industrial spray drying for example, such small particles are generated, indicating that the CDRC could result in a good prediction for the droplet drying kinetics in this process. Tran et al. apply the CDRC model in their CFD modeling of skim milk spray drying and found satisfactory accuracy for the entire range of modeled drying conditions [133]. Wawrzyniak et al. similarly utilized this approach to model the mass transfer in the droplets in their CFD simulations of a drying tower [134]. This approach has also been applied in CFD simulations of the drying of sucrose-maltodextrin solutions in a spray dryer by Woo et al. and for the drying of droplets of skim milk by Patel et al. [135,136,137]. Both these authors compared the CDRC approach with the REA, presented in the following section, and concluded that the REA was generally more reliable in delivering accurate results for high feed concentrations and high RH. Woo et al. also investigated a modified CDRC model proposed by Huang et al. where an extra complexity is added by allowing for a concave or convex shape to be expressed, instead of assuming a linear decay for the falling drying rate, more accurately describing reality [135,138].

#### 2.4.3. The Reaction Engineering Approach

The REA adopts the idea that the evaporation of liquid from a droplet is an activation process [139]. Hence, a certain energy barrier needs be overcome for evaporation to occur. It can alternatively be seen as a competitive process between an evaporation and a condensation reaction. The activation energy $\Delta E_{v}$ represents the difficulty for moisture to be evaporated from the surface. Depending on the amount of free moisture and the amount of already precipitated solids at the surface of the droplet, this parameter $\Delta E_{v}$ starts at a relatively low value but gradually increases during the lifetime of the droplet, indicating the eventually decreasing evaporation rate with decreasing moisture content.

Similar to the drying rate N of Equation (11), the change of mass over time $\frac{dm}{dt}$ for a droplet in the constant drying rate regime is described as:

(13) $$\frac{dm}{dt}=-h_{m}A\left(p_{v,sat}-p_{v,g}\right)$$

where $h_{m}$ is the mass transfer coefficient, A is the droplet surface area, and $p_{v,sat}\;$is the saturated vapor pressure at the droplet surface ($p_{v,s}=p_{v,sat}$) for a given surface temperature. The second, falling rate period is then captured by assuming that the vapor pressure at the interface $p_{v,s}$ decreases below $p_{v,sat}\;$over time due to the increased solute concentration at the surface of the droplet:

(14) $$\frac{dm}{dt}=-h_{m}A\left(p_{v,s}-p_{v,g}\right)$$

It is now assumed that the unknown, time-dependent $p_{v,s}$ is related to the saturation vapor pressure $\rho_{v,sat}$ as follows:

(15) $$p_{v,s}=\psi p_{v,sat}\left(T_{s}\right)$$

where ψ is a fractionality coefficient related to the liquid content at the interface and $T_{s}$ is the surface temperature. The coefficient ψ is unity when the complete surface is saturated by liquid, while ψ<1 when sufficient surface enrichment with solute results in the falling drying rate. Now, the concept of activation energy for evaporation $\Delta E_{v}\;$with an Arrhenius temperature dependency is applied to express a simple relationship for ψ:

(16) $$\psi =\exp\left(-\frac{\Delta E_{v}}{RT_{s}}\right)$$

where R is the universal gas constant. For droplets with a small Bi number (Equation (4)), the temperature at the surface *T_s_* can be approximated as equal to the temperature inside the droplet $T_{d}$, neglecting any presence of temperature gradients. Combining Equations (15) and (16), the following relation for the activation energy in the drying process $\Delta E_{v}$ is obtained:

(17) $$\Delta E_{v}=-RT\ln\left(\frac{p_{v,s}}{p_{v,sat}}\right)$$

By substituting Equations (14) in (17), the drying rate is:

(18) $$\frac{dm}{dt}=-h_{m}A\left(p_{v,sat}\exp\left(-\frac{\Delta E_{v}}{RT_{d}}-p_{v,g}\right)\right)$$

The mass loss rate$\;\frac{dm}{dt}$ can be rewritten in terms of liquid content X as:

(19) $$\frac{dm}{dt}=\frac{dX}{dt}m_{s}$$

where $m_{s}$ is the total solid mass dissolved in the droplet. If the liquid loss rate $\frac{dX}{dt}$ and the droplet temperature $T_{d}$ can be experimentally determined, one can also determine the activation energy $\Delta E_{v}\;$experimentally as:

(20) $$\Delta E_{v}=-RT_{d}\ln\left(\frac{-\frac{dX}{dt}\frac{m_{s}}{h_{m}A}+p_{v,g}}{p_{v,sat}}\right)$$

The activation energy $\Delta E_{v}$ can be normalized over the free moisture content $\left(X-X_{eq}\right)$ by dividing $\Delta E_{v}$ by the equilibrium activation energy $\Delta E_{v,eq}$ at conditions of equilibrium vapor pressure $p_{v,eq}$ and equilibrium droplet temperature $T_{d,eq}$ (see Equation (17)) to obtain values of $0\leq \Delta E_{v}/\Delta E_{v,eq}\;\leq 1$. This results in an explicit relation between the normalized activation energy $\Delta E_{v}/\Delta E_{v,eq}$ and the droplets liquid content $\left(X-X_{eq}\right)\;$and thus a characteristic curve for a certain system:

(21) $$\frac{\Delta E_{v}}{\Delta E_{v,eq}}=g\left(X-X_{eq}\right)$$

By performing a relevant drying experiment of the system under investigation, the REA parameters can be obtained. The $\frac{\Delta E_{v}}{\Delta E_{v,eq}}$ mastercurve can then be applied for the same system with similar initial moisture content to predict the solvent loss at different drying conditions [139].

Since the first time this approach was presented by Chen et al., it has proven to accurately model the drying kinetics of a wide variety of different systems [139]. They applied the REA to the drying of various food related products and propose to view the relationship between activation energy and liquid content as a fingerprint of a material [139]. Chen et al. later reviewed the most important aspects of the modeling technique and compared it to the CDRC approach [109]. Har et al. applied REA to glass filament single droplet drying of mannitol and found that the REA was sensitive to its temperature dependent crystallization behavior allowing for direct interpretation of drying profiles [140]. Putranto et al. utilized REA to model the intermittent drying of mango tissues and kaolin for varying temperature and humidity by firstly deriving the relative activation energy from continuous convective drying [141,142]. They showed that this approach delivers accurate results while keeping the mathematical modeling simple and robust. Putranto et al. also utilized the REA on the drying of polymer solutions and infrared heating [143]. Lin et al. investigated the drying of cream and whey protein concentrate droplets with an improved glass filament method and compared the experimental results with REA model predictions [144]. Rogers et al. investigated monodisperse droplet drying under similar conditions to industrial spray drying and applied the REA to numerically predict final moisture content of skim milk particles [145]. Fu et al. experimentally investigated the REA model on lactose droplets with a glass filament single droplet dryer [146]. They concluded that the REA is accurate in modeling a wide range of different initial droplet sizes and drying conditions [146]. A composite REA was published by Patel et al. to model drying droplets containing multiple components with different weight fractions [147]. The REA has been successfully implemented in CFD modeling of spray dryers and compared to the CDRC method [135,137]. Jin et al. implemented the REA model for the first time in CFD modeling of a spray dryer for milk particles [148].

## 3. Pharmaceutical Applications

### 3.1. Single Droplet Drying vs. Spray Drying

As a result of the rapid drying kinetics in spray drying and the complexity of the processing conditions, difficulties are encountered regarding in situ monitoring of the droplets during drying (e.g., recording changes in the composition). Generally, in spray drying, formulation and/or process parameters are iteratively adjusted, only based on the analysis of the resulting dry particles, so no information of the drying process itself is obtained [1]. Although, there are a few cases of more detailed investigations. For example, the properties of the drying gas have been locally evaluated by placing humidity sensors and thermocouples at different places in the drying chamber [149]. Characteristics of the drying droplets have been mapped out during spray drying by flash-freezing with a liquid nitrogen sampling device developed by Pearce et al. [150]. These techniques, however, only provide some general information, thereby stressing the relevance of a more simplified version of the process (i.e., the previously discussed single droplet drying), allowing continuous follow-up of the particle formation process [1].

Single droplet drying experiments are increasingly attracting the attention of the pharmaceutical sector, mostly because they have proven to be of value for the optimization of manufacturing processes such as spray drying and to accelerate process development [1,50,67,151,152]. For example, the morphology of polymer–lactose particles prepared by spray drying was nicely reproduced with the single droplet Drying Kinetics Analyzer™ (DKA) by Nuzzo et al. [67]. They found that particle morphology was mainly influenced by the surface rheological properties of the feed solution (i.e., dilatational modulus), rather than the extent of surface coverage. Surface compositions of the resulting particles were similar, although higher levels of the surface-active polymers were found at the surface of the levitation-dried single particles as compared to spray dried particles, which they attributed to a different time-scale of the drying process (i.e., being a longer drying time for the single droplet drying experiments) [67]. Both et al. obtained similar particle morphologies for single droplet drying and pilot-scale spray drying of whey protein and maltodextrin solutions [153]. Gouaou et al. demonstrated that optimal conditions for spray drying of HPS, used for pharmaceutical coating applications, can be determined via SDD experiments [50]. Additionally, in the food industry, single droplet drying is used to get a better understanding of the spray drying process by identifying critical phenomena during drying. CFD simulations of the spray drying process can be based on the results obtained from single levitated droplet drying experiments. For example, Ullum et al. found that the particle morphology of the single droplet dried particle agreed well with the morphology of the spray dried particle, if processed under conditions similar to the outlet conditions of the spray drying process [152]. Even in atmospheric and climate sciences, a more profound understanding of aerosol properties is generated through measurements of single particles, as nicely reviewed by Krieger et al. [154]. Nevertheless, similar results for single droplet drying and spray drying of the same formulations are not always obtained. Pajander et al. studied mannitol polymorphism by means of XRD and Raman mapping [155]. When mannitol/lysozyme formulations were spray dried, both β- (i.e., thermodynamically most stable form) and α-mannitol were present, and the α-mannitol fraction increased with increasing lysozyme content. In contrast, α-mannitol and δ-mannitol were observed after single droplet drying experiments with a DKA [155].

Despite the relatively high predictive value of single droplet drying, there are some aspects that should not be overlooked, as the drying conditions are evidently not the same as in the drying chamber of a spray dryer. Firstly, all cases of single droplet drying start from larger droplets. According to Vehring et al., no droplets smaller than 170 µm are investigated with single droplet drying, which greatly differs from the particle size obtained with spray drying (i.e., approximately 10 µm and 40 µm for a laboratory spray dryer and industrial spray dryer, respectively) [3]. The droplets can be even up to ten times larger compared to droplets created in an industrial spray dryer, which of course influences dynamic processes like mass and heat transfer as discussed in Section 2.2.1, and prevents an accurate simulation of the drying kinetics [1,67,152,156]. The fact that only larger droplets are investigated with SDD techniques can be explained by the difficult manipulation required to put the droplet within the system and by the short drying time of smaller droplets [157]. This size difference undoubtedly has an impact on the predictive power of single droplet drying, since larger droplets also imply larger resulting particles, following Equation (1) [158,159]. The droplet size also influences the morphology development during drying and with respect to the different drying stages and influencing parameters mentioned in Section 2.2, it takes longer for larger droplets to reach the critical moisture content, hence possible crust formation will occur later [160,161]. In order to increase its predictive value, there is thus a major need for novel (single) droplet generators that can create droplet sizes similar to those generated in an industrial spray dryer. The time-scale of the drying process is the second important difference between single droplet drying and spray drying, with a longer drying process for the former (i.e., minutes compared to (milli)seconds) [67]. This drying time difference can have implications for the particle morphology, as stated by Both et al., more specifically whether the time allows an inhomogeneous component distribution or not [153]. Thirdly, the benefit of single droplet drying as a simplified, idealized version of spray drying inherently entails that this method lacks information on droplet–droplet interactions, droplet-wet particle interactions and droplet-drying chamber wall collisions [1]. Additionally, the spraying process itself will have an influence on the pattern of the air flow inside the drying chamber, which in turn has an impact on the trajectories of the droplets and resulting particles. In the following section, the interactions within a spray will be discussed in more detail.

### 3.2. Interactions within a Spray

Interactions between droplets in a spray will affect the droplet drying process, and have been reported to reduce the transfer coefficients compared to single droplet drying [162]. A significant amount of droplet collisions can be expected in the region near the atomization device and in recirculation zones, because of the large relative velocity and high droplet concentration [163]. To take into account these transfer coefficient differences coming from droplet–droplet interactions, studies on chains of monodisperse free-falling droplets have been conducted [3].

**Figure 7:**
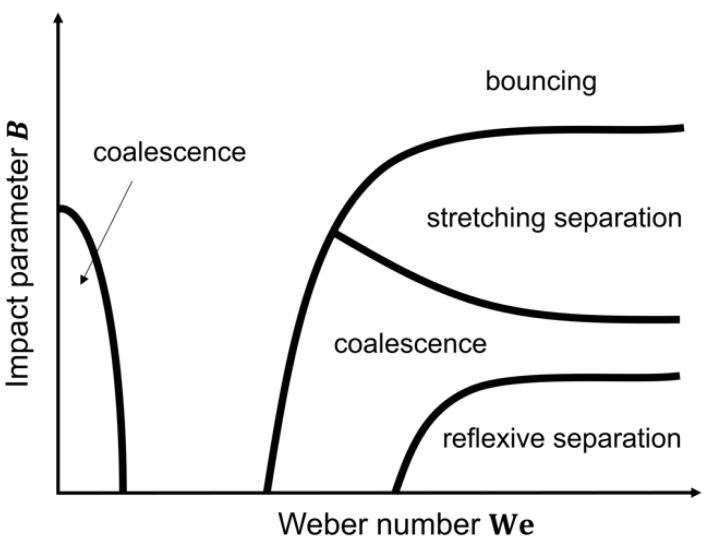
Collision map (modified from Li et al. [163]).

**Figure 8:**
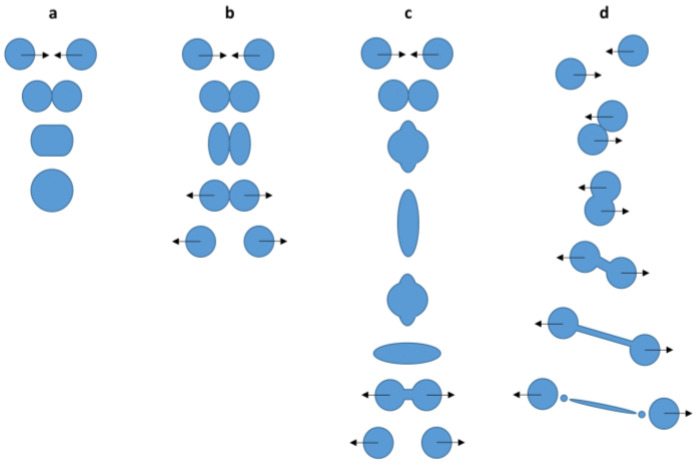
Schematic illustration of the four collision regimes: (**a**) coalescence, (**b**) bouncing, (**c**) reflexive separation, and (**d**) stretching separation (modified from Charalampous et al. [167]).

In general, there are four collision outcomes, which can be illustrated in the impact parameter (*B*)—Weber number (We) collision map as depicted in Figure 7 [163]. The impact parameter *B* is a measure of the geometry of drop collisions. More specifically, *B* equals zero for head-on collisions (i.e., the position vector connecting the middle points of the two droplets coincides with the relative velocity vector), while *B* larger than zero corresponds to off-center collisions (with *B* approaching unity for only tangentially touching collisions). The We number measures the ratio of the inertial force to the surface tension force and can be calculated as follows:

(22) $$\mathrm{We}=\frac{\rho \;u^{2}d}{\sigma }$$

where ρ is the density, u is the collision velocity [m s^−1^], d the diameter of the droplet and σ the surface tension. When head-on collisions occur, four different collision regimes can be identified with increasing We number. It is worth mentioning that the boundaries between these regimes are influenced by the properties of the feed solution (e.g., concentration and hence viscosity) and the drying gas environment. Studies that investigate the effect of feed viscosity on collision outcomes are nicely reviewed by Finotello et al. [164]. At low We number, coalescence of two individual droplets into one larger droplet takes place. For higher We numbers, bouncing of two distinct droplets occurs, subsequently followed by coalescence again. Note that bouncing relates to the lubrication flow and discharge of the gas layer between two approaching droplets and occurs for the whole We number range for *B* values close to unity. The phenomenon of reflexive separation only takes place beyond a particular critical We number and low impact parameter. In this case, the droplets initially merge, resulting in a disc-shaped droplet which is subsequently contracted by the effects of surface tension. Moreover, a reflexive internal flow is induced that forms a cylindrical droplet (of which the axis is perpendicular to the one of the disc) that eventually breaks up. In contrast, for a high impact parameter and thus lower degree of droplet–droplet interaction, stretching separation occurs (Figure 8). Due to the momentum of the non-interacting regions, stretching of the merged area occurs, thereby forming a ligament between the colliding droplets and eventually break up takes place [165]. Both separation mechanisms can result in the formation of little satellite droplets [166]. All of the above described outcomes of droplet collisions will broaden the droplet size distribution of the spray drying process [163].

Despite this well-established knowledge about droplet collisions in a spray, challenges remain concerning their implementation into spray drying models. According to Nijdam et al., it is difficult to accurately measure coalescence rates in a spray and therefore to properly model gas-droplet and droplet–droplet turbulence interactions [6]. In addition to the droplet interactions, a number of important phenomena occur within the spray dryer that strongly govern the extent of dry particle agglomeration. Firstly, glass-transition temperature and moisture evaporation effects are considered important since they both influence the stickiness of the particles. Next to that, turbulent dispersion effects occur, which produce relative velocities between particles due to the turbulent motion of the gas flow, so that these particles can collide [6].

Several studies modeling droplet–droplet collisions during spray drying are available in literature. Mezhericher et al. developed a theoretical model, based on an Eulerian approach for the continuous phase (drying gas) and a Lagrangian approach for the spray of droplets (the discrete phase) for CFD simulation of the spray drying process [168]. Droplet–droplet interactions were treated by a probabilistic approach. Despite the fact that droplet–droplet interactions were implemented in this model, a few assumptions were made in order to simplify the calculations. Breakage of droplets after collision is not modeled and rotational movements of the droplets are neglected. Moreover, the droplets were regarded as pure water droplets, without dissolved solids. The researchers found that droplet–droplet interactions (i.e., coalescence and bouncing) influence temperature and humidity profiles and hence that they displace the region of heat and mass transfer from the central core towards the drying chamber periphery. Coalescence will lead to the formation of larger droplets, implying that a longer time for evaporation is needed. More droplets with a substantial amount of moisture will head towards the periphery of the drying chamber, where they are evaporated and thus result in an increased vapor mass fraction in the drying air and a reduced drying air temperature [168]. Finotello et al. investigated droplet–droplet interactions with a Eulerian-Lagrangian model, comprising turbulence dispersion to account for fluid velocity fluctuations [169]. However, this study was conducted in an isothermal spray system in the absence of a drying procedure. This study concluded that with increased viscosity of the feed solution, more droplet coalescence occurs because the viscous dissipated energy reduces the kinetic energy that is available to separate merged droplets. Due to coalescence, the number of droplets available for collision, and therefore the frequency of collision, decreases [169].

### 3.3. Parameters that Influence the Drying Process

In any application where particles are produced, control of the particle size and its distribution, and morphology, is pivotal. Size and morphology both strongly influence the bulk characteristics of the resulting powder and determine inter alia bulk density, flowability, compressibility, reproducibility of dosing, dissolution, and aerodynamic properties [159,170]. In the following section, the impact of process parameters, formulation parameters and component characteristics on both particle size and morphology is elaborated for spray drying as well as for electrospraying. These two technologies can be considered as most general applications of this review article and are highly important within the field of amorphous solid dispersions (ASDs) [171].

#### 3.3.1. Parameters that Influence the Spray Drying Process

**Figure 9:**
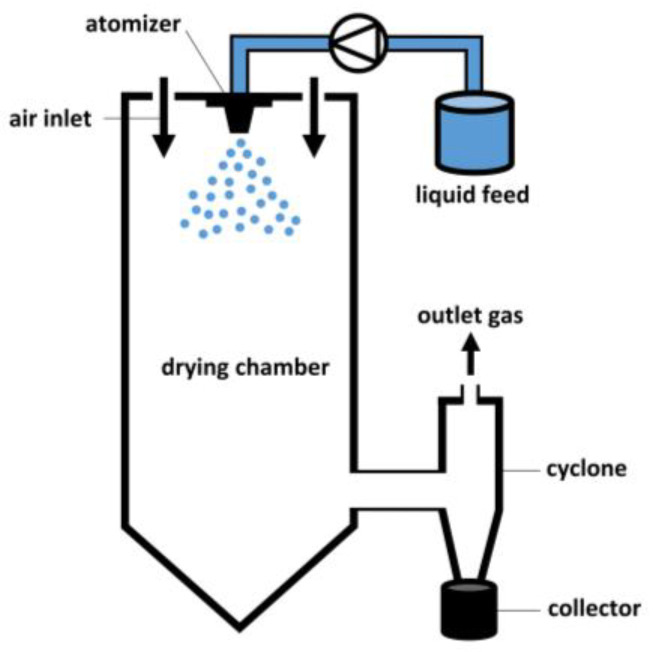
Schematic diagram of a spray drying setup.

Spray drying is a well-established and widely applicable process that has been implemented successfully in the pharmaceutical, chemical and food industry, and even finds its use within cosmetics, fabrics, and electronics [5]. A schematic diagram of the spray drying process can be seen in Figure 9. In spray drying, a solution is atomized through a nozzle, thereby generating small droplets which are subsequently dried due to very fast solvent evaporation in the drying chamber. The atomization step is highly important in generating a sufficiently large surface area over which heat and mass transfer can take place. Heat is transferred from the hot drying gas in the drying chamber towards the droplet surface while vapor transfer occurs from the droplet surface towards the carrier gas. Finally, the resulting dried particles are separated from the drying gas via a cyclone and collected in a collection vessel [5]. 

##### Process Parameters

*The Nozzle*

The production of an ensemble of multiple droplets with a specific size distribution is achieved through atomization. For the atomization of the feed solution, the most widely used nozzles in the food and pharmaceutical industry are pressure nozzles, two-fluid nozzles, and rotary atomizers. Recently, also ultrasonic nozzles are applied, although their use is currently limited to a lab-scale setting [30]. The working principle of an ultrasonic nozzle is based on high frequency sound waves that are converted into vibratory motion at the same frequency by piezoelectric transducers. The motion is then magnified via titanium cylinders until the waves collapse and droplets are formed. The diameter of the generated droplets is tunable by varying the feed rate and/or the vibration frequency of the nozzle [33]. Pressure nozzles contain swirl inserts or spiral grooved inserts and rely on the application of pressure on the liquid (mostly between 30 and 200 bar) to generate a cone-shaped spray pattern [157]. The nozzle diameter is mostly larger than 1 mm and they are the primarily used atomizer nozzles in industrial spray dryers. Droplet size ranges from 20 to 200 µm and is determined by the ratio of the applied pressure to the solution feed rate, resulting in smaller droplets for higher pressures. However, it is noteworthy that larger pressures (i.e., of more than 70 bar) also lead to wider droplet size distributions [157]. Smaller droplets can be obtained by decreasing the diameter of the nozzle orifice (which is independent of the type of nozzle used), but then one must be aware of an increased risk of clogging [30]. The atomization step also results in smaller droplets when a feed solution with a lower surface tension or viscosity is used, again independent of the nozzle type [31]. Two-fluid nozzles, in contrast, are mostly used in lab-scale spray dryers and create smaller droplets (and hence smaller particles) than pressure nozzles. Droplet sizes of approximately 5 to 75 µm are obtained. This kind of atomization system applies pressurized air to break up the liquid feed into individual droplets, whose size depends on the ratio of the pressurized air flow rate to the liquid feed rate. The atomizing gas is applied via a ring-shaped opening around the feed orifice, of which the diameter is mostly between 0.5 and 2 mm. Compared to pressure nozzles, the energy that is needed for the droplet generation in two-fluid nozzles is approximately 10 to 30 times higher, however, smaller spray angles are obtained [157]. Because the velocity of the atomizing air is significantly larger than the concurrently flowing drying air, the Venturi effect occurs, resulting in a risk of backflow and clogging when two-fluid nozzles are used [159]. Rotary atomizers operate through centrifugal forces, where a rotating wheel (up to 50,000 rpm) distributes the feed and determines the spray pattern. Here, the droplet size depends on the ratio of the wheel speed to the liquid feed rate and is typically between 20 and 200 µm. If a rotary atomizer is applied, dimensions of the drying chamber need to be adapted (i.e., enlarged) in order to avoid material adhesion to the drying chamber walls [30].

Each spray, created in the atomization step, contains a range of different droplet sizes. This droplet size distribution is critical for an effective spray drying process since it has an impact on the evaporation rate of the solvent and ultimately on the performance of the resulting particles. Moreover, the polydispersity of the spray inside the drying chamber encompasses complex droplet trajectories and droplet–droplet and droplet-drying chamber wall collisions [30,31,157]. Every droplet in the spray will have a different route inside the drying chamber and experience diverse humidity-time and temperature-time profiles, which causes additional variability in the resulting dried product [30]. The variations in particle size, particle morphology and microstructure affect final product properties and downstream processing, as stated by Liu et al. [31]. Lee et al. for example showed that polymorphic forms can change as a function of particle size [172]. For the smaller particle fraction, metastable polymorphic forms of mannitol were obtained, and this can be explained by the fast drying conditions smaller droplets experience. Larger droplets will dry more slowly, resulting in crystallization into the thermodynamically most stable polymorphic form of mannitol [172].

In order to obtain uniform, high-quality products, a lot of research is conducted within the scope of attaining monodisperse droplet size distributions, as already indicated in the section where free-fall methods are discussed to create single droplets. Only in this case, equal or at least comparable drying histories can be foreseen for all generated droplets [157]. For the production of monodisperse droplets, formulation parameters (such as density, surface tension, and viscosity) and process parameters (liquid feed rate) need to be controlled within the Rayleigh jet breakup zone [30,173]. This zone distinguishes itself from the atomization zone on the basis of the relative importance of forces in the system. According to Wu et al., uniform droplet sizes will be obtained when the dimensionless Reynolds (Re) and Ohnesorge (Oh) number are relatively low, whilst high numbers result in producing a spray [30]. These non-dimensional numbers are defined as follows:

(23) Re=ρgrvμ

(24) $$\mathrm{Oh}=\frac{\mu }{{\left(\rho r\sigma \right)}^{1/2}}$$

where ρ is the density of the liquid feed, μ is the viscosity, r is the characteristic length (i.e., nozzle diameter), v is the characteristic velocity, g is the gravitational acceleration, and σ is the surface tension of the feed solution [30].

Generating arrays of single droplets can be achieved by using MDGs, firstly developed within the framework of ink-jet printing technology [173]. Later on, Patel and Chen attempted to implement these innovative and printing industry-based atomizers in a lab-scale spray dryer, with the aim of producing uniform-sized and spherical particles [174]. In their setup, a MHDG equipped with a piezoelectric transducer is applied for breaking up the liquid stream into individual droplets. With the so-called Ink-Jet Spray Dryer (IJSD), good control over the droplet size distribution, the trajectories of the droplets and collisions was achieved [174]. Therefore, the use of MDG in a spray drying process can lead to a more analyzable and controllable droplet drying process while implying less wall depositions, less product loss, and hence less waste of energy [30]. As can be deducted from the formula of the Reynolds number, higher velocities (i.e., higher feed rates) will result in a higher Reynolds number and hence in the production of a spray (i.e., atomization zone). Generation of monodisperse droplets, so within the Rayleigh jet breakup zone at low Re, inherently indicate a low feed-handling capacity. Thus, with a view to the high production capacity of an industrial spray dryer, this feed-handling capacity must be markedly increased. This issue is mostly overcome by creating an array of multiple nozzles since MDGs can easily be grouped in one mechanical assembly [30]. Patel and Chen for example equipped their IJSD with multiple MHDGs, in order to enhance the production capacity [174]. It is noteworthy that the IJSD operated in continuous mode, so continuous series of periodic pulses were applied to the transducer. In contrast, Vehring et al. developed a droplet chain technique in droplet-on-demand mode, where periodic pulses are only applied to the transducer when a droplet is desired [3]. In this way, an increased distance between the individual drops can be attained, therefore, droplet–droplet interactions are further reduced. Since the throughput is even lower when operating in droplet-on-demand mode, Vehring et al. also developed a monodisperse spray dryer, where a vibrating orifice monodisperse aerosol generator, originating from the printing industry and further elaborated by Berglund et al., was utilized as atomizer [3,30,175].

In addition to the MDG, literature also describes less frequently used methods to narrow the droplet size distribution regarding spray drying. One of them is the Rayleigh breakup of threads, created at a multitude of capillaries under laminar flow conditions, which can be applied on industrial scale. Another technique based on the concept of Rayleigh breakup, is the laminar-operated rotary atomizer, where the liquid feed solution is distributed on the inside wall of a rotating cylinder. A third way to diminish the droplet size distribution is via a jet cutting system. This system is made up of a wheel, containing thin wires that serve as spokes. According to P. Walzel, this method can be applied on pilot-scale [157]. Even combination systems are applied for precise control of the size distribution. Berkland et al. combined a piezoelectric MHDG with a flow of non-solvent carrier around the monodisperse droplet jet for a more extensive control of the droplet size [176].

Atomization Gas

Various gases such as air, N_2_, and CO_2_ can be applied for atomization purposes in spray drying. The different physical properties of the gases (e.g., density) can affect the atomization and drying process. Gases with lower densities should preferably be chosen when one wants to obtain smaller droplet sizes (thus particle sizes) and higher droplet velocities [5,177].

Pressurized Air Flow Rate

Increasing the pressurized air flow in a spray dryer with a two-fluid nozzle results in smaller droplets, because of the higher relative velocity and larger shear forces [159,161]. However, Kemp et al. found that droplet size tends to an asymptote with increasing pressurized air flow rate [161]. Above 0.4 g/s, the pressurized gas approaches sonic velocity, although pressure and momentum can still increase [161]. Smaller droplets have a smaller time window for evaporation, that results in spherical, smooth but hollow particles because of rapid crust formation, according to the study of Vicente et al. [159]. Larger droplets will dry slower and thus allow for solids diffusion to the center of the drying droplet, which results in more shriveled particles or dense particles [159]. Note that these morphologies are not directly expected from the relationship between droplet diameter and Pe number (see Equation (2)). However, this equation also entails the evaporation flux, that is probably the decisive factor in this case. Different particle morphologies are hence obtained when varying the pressurized air flow rate and/or liquid feed rate.

Liquid Feed Rate

Droplet sizes generally increase with increasing liquid feed rate. However, Kemp et al. found that droplet sizes appear to increase with decreasing liquid feed rates for rates below 5 mL/min, which was attributed to a pulsing flow, provided by a peristaltic pump, resulting in non-effective atomization [159,161]. When increasing the feed rate, more energy is required for solvent evaporation and hence the outlet temperature in the spray drying process will decrease [5]. According to Finotello et al., the liquid flow rate can be considered as the most important parameter for the number of droplet collisions during spray drying [164].

Environmental Conditions

Drying Temperature and Relative Humidity—The drying temperature also has a great impact on the particle morphology, as already discussed for single droplet drying. In case that the outlet temperature of the spray drying process is above the boiling point of one of the solvents, the resulting particles will inflate after shell formation due to an increased vapor pressure created inside the particle, especially if low feed concentrations are used, and thus a flexible shell is formed [159]. This formation of inflated particles when operating above the boiling point, was already discussed for SDD experiments. When a high feed concentration is combined with a high drying temperature (spray drying at high evaporative conditions), the onset of shell formation is so fast that atomization could become inadequate resulting in the generation of string-like particles [159]. Bigger particles are obtained for higher drying air temperatures for spray drying due to the Pe increase as discussed for SDD, due to an earlier crust formation. These particles are mostly characterized by lower densities because of the greater tendency to be hollow [153]. Furthermore, with increased drying air temperature, the thickness of the crust is reduced, which can also be related to an increased Pe [50]. The outlet temperature of the spray drying process also strongly affects the roughness of the resulting particles, as studied by Littringer et al. [178]. High outlet temperatures during pilot-scale spray drying of mannitol led to the formation of smoother particles, due to the presence of smaller crystals on the particle surface. In contrast, when processed at low outlet temperatures, rough surfaces are generated. This difference in particle roughness is, in this specific case, presumably caused by different rates of mannitol crystallization. It is noteworthy that the trend was the other way around for lab-scale spray drying, with rougher particles when spray drying at high outlet temperatures. This surprising conclusion can be attributed to different crystallization mechanisms as droplet sizes, and thus evaporation rates, vary between lab-scale and pilot-scale spray dryers [178]. In another article, Littringer et al. describe the influence of higher outlet temperatures on the mannitol particle shape [179]. For higher temperatures, hence smaller crystals on the surface, the pores become smaller and hamper the evaporation of water that is still trapped inside the drying droplet. For this reason, a pressure might form inside the particle that lead to particle shell collapse and thus explaining the multiple surface indentations [179]. Kim et al. investigated the effect of drying temperature for the spray drying process, which was equipped with a centrifugal atomizer, and observed a decrease in the content of surface fat by increasing the drying temperature [180]. They observed the same trend as for concentration, namely the content of surface fat of spray dried milk powders decreased with increasing feed concentration, as will be discussed in the formulation parameter section. Rapid crust formation preventing fat migration towards the surface serves as a plausible explanation [180]. It is very interesting to note that this drying temperature effect is more pronounced at low feed concentrations, emphasizing their interdependency. In the case of amorphous solid dispersions, additional matters need to be taken into account. For example, the outlet temperature of the spray drying process should not exceed the glass transition of the drug–polymer system, otherwise stickiness occurs, resulting in low process yields [5]. Next to this, the drying temperature needs to be installed in such a way that kinetic trapping of the drug in the polymer matrix, i.e., very rapid solvent evaporation that prevents phase separation and thereby crystallization, becomes possible. Kinetic stabilization enables the formulation of higher drug loadings than the thermodynamic solubility of the drug in the polymer matrix [181]. However, it should be mentioned that the ability to kinetically trap the drug does not only depend on the drying temperature, but also on other features such as the droplet size and the drug–polymer miscibility.

By changing the RH of the drying environment, the point of solid layer formation and the resulting particle size can be manipulated [25]. Because of the challenges concerning in situ monitoring during spray drying, the effect of RH is mostly studied in single droplet drying experiments and therefore explained in more detail in Section 2.2.1. From those studies, it can be anticipated that smaller, more homogeneous, and less porous particles are formed with increasing RH and thus lower Pe. Both et al. studied the effect of the outlet air humidity on particle morphology after pilot-scale spray drying with a pressure nozzle [153]. Variations in outlet air humidity were achieved by changing the nozzle pressure, which influenced the feed rate. The authors observed no pronounced effects of the outlet air humidity on particle morphology, since the onset of morphology formation happens in the very initial period of the spray drying process [153].

Drying Gas—As for atomization gases, also various drying gases can be applied for spray drying, with a possible impact on heat and mass transfer. Most often, air is used as drying gas, however also inert atmospheres can be created with N_2_ in order to prevent oxidation of the products. Higher heat and mass transfer are obtained with CO_2_ as drying gas [5]. Different drying gases also result in differences in process yield. For example, the best process yields were generated for spray drying lactose in air compared to N_2_ and CO_2_ as studied by Islam and Langrish [177]. Additionally, different lactose particle morphologies were obtained after spray drying in different media. Spherical and smooth particles were obtained for air and CO_2_, but sharp-edged particles (attributed to crystallization) were created when N_2_ was used [177].

##### Formulation Parameters

*Solute Concentration*

Within the field of ink-jet printing, the effect of solids concentration on particle size was investigated by Udey et al. [33]. A THDG was applied and the resulting 30 µm glucono-delta-lactone (GDL)/water droplets were subsequently directed to desiccant aerosol dryers, where the particle formation process took place. They observed that, for constant droplet sizes, higher GDL concentrations (ranging from 1 % to 20 % *w/v*) lead to larger microparticle diameters, as measured with an aerodynamic particle sizer (APS). This is in accordance to Equation (1) and the discussion in the SDD part of this review. In this context it should be mentioned that, with increasing solids concentration, density and viscosity of the feed solution increased. According to Udey et al., solutions with higher viscosities have longer break-off times, implying that larger droplets are formed compared to low-viscosity solutions, which in turn leads to larger particles [33]. No differences in particle morphology were identified for the different concentrations as all resulting particles were spherical. Despite the fact that this research is situated in the ink-jet printing industry and thus that the setup is slightly different from a conventional spray dryer, the outcome may also be valid in the context of spray drying. However, Vicente et al. state that solids concentration only affects particle size in a substantial way when dilute solutions are used, being from 1 wt% to 5 wt% [159]. For higher concentrations, particle size is mostly influenced by the atomization process and hardly by concentration [159]. This positive relationship up to 5 wt%, followed by leveling off was also observed by Elversson and Millqvist-Fureby [158]. However, this effect is only valid in case two-fluid nozzles are used. When for instance a pressure nozzle is applied, and feed viscosity becomes a critical parameter, feed concentration has a major impact on droplet size and hence on particle size [159]. Liu et al. spray dried chitosan solutions of different concentrations but no effect on particle morphology was seen [36]. In contrast to Udey et al. and Liu et al., Vicente et al. presented different particle morphologies after spray drying different concentrations of HPMC-phtalate (HPMCP) in methanol/water [159]. With increasing concentration, and thus increased viscosity, the mobility of the dissolved solids is restricted. At high temperature (i.e., high evaporation rates and thus Pe), perfectly spherical particles were obtained due to early stable crust formation. On the other hand, when a lower temperature was installed, shriveled particles were created since the time window for solute diffusion and shell movement was prolonged [159]. In the case of low feed concentrations, where mobility is not restricted, phenomena such as shriveling, inflation and particle breakage were observed, and the same conclusions can be drawn from the single droplet drying part of this review. In this regard, Vicente et al. set forth that a minimum concentration is needed to mechanically stabilize particles. Below that concentration, the particle size will be dependent on the mobility and flexibility of the shell and does not follow the trend of the droplet size [159]. The same explanations were used in the research of Gouaou et al., although it was mentioned in the context of relating the particle size of spray dried HPS to its feed concentration, namely an early crust formation (due to increased feed concentration) will result in larger particles [50]. Next to that, they could link a higher feed concentration to higher powder recovery during the spray drying process (i.e., equipped with a two-fluid nozzle). An increased viscosity generally produced a narrow spray pattern and angle. However, at too high feed concentrations, 30 wt% in their case, the spray collapsed and stuck on the nozzle [50]. It was also found that, with increasing feed concentration, bulk and particle density increased [159]. Moreover, different feed concentrations can lead to dissimilar compositions of the particle’s surface. According to Kim et al., the content of surface fat of spray dried milk powders decreased with increasing feed concentration, as determined with electron spectroscopy for chemical analysis (ESCA) [180]. Fat is preferably present at the surface of atomized droplets but with higher feed concentrations, their mobility is restricted due to a higher viscosity [180]. Pajander et al. spray dried mannitol/lysozyme formulations and concluded that spherical and smooth particles were generated if the lysozyme concentration is low, whilst raisin-like particles were created for high lysozyme concentrations (50 wt%) [155]. This was attributed to lysozyme adsorption to the liquid–gas interface of the droplet that hinders the solvent evaporation and hence prolongs the drying time [155]. In addition, the effect of feed concentration on particle surface roughness was studied. Littringer et al. found that higher feed concentrations imply rougher mannitol particle surfaces, due to the presence of larger mannitol crystals [179]. Higher concentrations, and thus, higher viscosities alter the drying kinetics and consequently the crystallization behavior of mannitol. However, the effect was only detectable at low drying temperatures [178].

Surface-Active Components

As already indicated above, the inclusion of surface-active compounds like fatty components or proteins in the feed solution can result in different particle morphologies. Indeed, most surface-active compounds are adsorbed at the surface of the atomized droplets and hence dominate the surface of the resulting particles [9]. Maa et al. observed a smoother particle surface when surfactant (i.e., polysorbate) was added to the solution and explained that the presence of surfactants reduces internal motions and surface turbulence of the droplets during spray drying [42]. However, the effect of surface-active components on the particle morphology is not unambiguously defined in literature. As already indicated for single droplet drying, the outcome of adding surfactants to the solution strongly depends on the type of surfactant used, in particular its surface activity and molecular weight. Lechuga-Ballesteros et al. reported morphology changes from more spherical towards more corrugated particles by increasing the amount of trileucine, a surface active tripeptide, in the solution and attributed this to the fact that trileucine was the least soluble compound [182]. The reader should also keep in mind that surface-active compounds might change solubility values of the components to be spray dried and in that way might influence particle size and morphology. Next to surface activity, component distribution in particles is related to the diffusional flux due to evaporation, and thus the Pe number, but also the time for a particular component to reach saturation at the surface [67]. Kemp et al. studied the effect of surfactant on the droplet size and noticed that the droplet size, and hence the particle size, decreases by increasing the surfactant concentration in the feed solution [161]. It is important to mention that this formulation parameter effect is dependent upon the process parameters used. The surfactant effect was most pronounced at low atomization air flow rates and negligible if high atomization air flow rates and low feed rates were applied [161].

Solvents

The solvent plays a key role in spray drying, and the particle formation process can be influenced by physicochemical properties of the solvent, such as its volatility, polarity, and the solvent quality for given solutes. As discussed for the SDD above, Rizi et al. pointed out that the dissolution capacities and diffusional fluxes of polymeric feed solutions vary for different solvent systems [183]. Eudragit L100 particles prepared from a pure ethanol solution exhibit a shriveled morphology, while more spherical particles are obtained when water is added to the solution, which can be explained by differences in polymer solubility. Furthermore, Eudragit L100 will have a larger hydrodynamic diameter due to polymer coil unfolding and larger intrinsic viscosities by adding water to the ethanol solution, resulting in lower diffusional fluxes [183]. Another study reveals that particle size and surface chemistry are highly dependent on the solvent power of the feed solution. With methanol, considered as a poor solvent for poly(lactic-co-glycolic acid) (PLGA), smaller particles were generated. According to Wan et al., this was attributed to the lower volatility of methanol compared to acetone and thus to a slower drying rate, but also to the lower viscosity of the feed solution [184]. Additionally, the drug concentration at the surface of the resulting particles was higher when methanol was used as a solvent, relying upon the distinctness between the solubility of the drug and that of the polymer [184]. A more detailed elaboration on the influence of solubility of the dissolved substances on particle size and morphology is provided elsewhere in this review (see section Component Characteristics).

##### Component Characteristics

*Molecular Weight*

Next to SDD, the molecular weight of the dissolved substances also has a strong influence on the particle morphology for spray drying. Large molecules, like proteins and polymers, are typically characterized by a high Pe number because of their limited diffusivity. Spherical hollow particles are the result if the crust is rigid, otherwise wrinkled particles are created [9]. As mentioned in the previous section, the polymer expansion and thus its effect on the viscosity is strongly dependent on the solvent. According to Liu et al., lower molecular weights of chitosan, obtained after controlled degradation reactions, imply that the formed crust could easily collapse, hence resulting in inflated particles [36]. However, the authors acknowledge the impact of adding lactose to the chitosan solution on particle morphology. Lactose has a much lower molecular weight than chitosan and is therefore more mobile. In this way, lactose can reduce the shrinkage and more spherical particles can be obtained [36]. Clarke et al. found that low molecular weight polymers led to spherical particles, whilst for high molecular weight polymers, more concave particles are produced [185]. When these researchers spray dried solutions of higher molecular weight polymers (i.e., approximately 100,000 g/mol), microparticles can only be produced at low feed concentrations, namely below 2% *w/v*. If higher feed concentrations are applied, fibers are formed, and this is probably due to strong intermolecular bonds and adhering stiffness of the polymer chains [185].

The influence of the diffusion coefficient of the solutes on particle morphology is the same as discussed for single droplet drying. Components with low diffusion coefficients (i.e., high Pe number) will lead to crust formation and hollow particles, whether substances with high diffusion coefficients (i.e., low Pe number) will form solid and dense particles. However, a constant evaporation rate and an invariable diffusion coefficient are assumed, irrespective of the time and location in the drying droplet. In reality, the diffusion coefficient of the solute varies during drying with composition of the solvent and with concentration. The Pe number can furthermore change if a phase transition, for instance crystallization or liquid crystal formation, takes place during the drying process and is reported in literature for low solubility amino acids and small peptides. If these low solubility molecules have a large diffusion coefficient, their concentration will increase during the drying process without much surface enrichment, until they reach supersaturation and precipitation occurs. At this point, the component’s mobility is now determined by the lower mobility of the precipitated phase, rather than by the diffusion coefficient of the dissolved phase, resulting in hollow particles. These observations underpin the importance of the mutual influence between solubility and diffusion coefficient and thus between different properties of the starting materials in general [9].

Solubility

Larger particles can be produced when decreasing the solubility of the solute. As seen for the SDD, a low solubility results in an earlier precipitation and crust formation (i.e., the saturation limit is reached earlier). For example, Elversson and Millqvist-Fureby obtained smaller particles for carbohydrates with the highest solubility in water and Bain et al. obtained larger particles when poly(lactic acid) (PLA) was dissolved in a solvent in which it was least soluble [158,186]. Moreover, in the latter case, a more open and porous structure was created by spray drying. In contrast, if PLA could be well dissolved in a particular solvent, more coherent particles of greater density were obtained [186]. Elversson and Millqvist-Fureby also reported the influence of the crystallization propensity of the carbohydrate used. Hollow particles are formed by amorphous carbohydrates such as lactose and sucrose, whereas porous particles are more likely to be generated from crystalline carbohydrates like mannitol [158]. When the dissolved substances are highly soluble in the particular solvent, it takes longer to reach the saturation limit at the surface and to precipitate at the droplet’s interface, therefore resulting in solid and dense particles if a moderate drying temperature is installed [9]. In case of low solubility, particle morphology will depend on the existence of phase transitions, as set out in detail in the molecular weight section above [9]. Nevertheless, contradictory particle morphologies that were dependent on polymer solubility are reported. Wang et al. investigated the particle morphology after spray drying of PLGA dissolved in different organic solvents and encountered spherical and porous particles for PLGA dissolved in a solvent wherein it was highly soluble (i.e., DCM) [187]. For another solvent (i.e., ethyl acetate), in which PLGA was less soluble, non-porous and doughnut-like particles were obtained, which complies with the findings for single droplet drying. As discussed before, in single droplet drying experiments, a higher solubility results in smooth and spherical particles, while wrinkled and collapsed particles are produced for low solubility values. In the case of ethyl acetate, larger particles were obtained, which is thus consistent with the research of Elversson and Millqvist-Fureby and Bain et al. [158,186,187].

Solid-State Characteristics

When manufacturing ASDs, the glass forming ability (GFA) or crystallization tendency of the drug needs to be taken into account [188,189]. The GFA in turn depends on the applied manufacturing method, the solvent used (in case a solvent based ASD manufacturing technique is applied) and on the storage conditions. It will partially determine the physical structure of the powder, which comprises its solid state, namely crystalline or amorphous, and the miscibility of the components with one another. As a single-phase amorphous system is less prone to crystallization than a phase separated one, the miscibility of the components should be considered as well to formulate an ASD with an acceptable physical stability. Next to their impact on the stability of the system, these solid-state properties can also influence the drying process and thereby the particle morphology that is obtained. This was, for example, demonstrated by Wulsten et al., where the solid state of itraconazole determined whether the surface was wrinkled (crystalline) or not (amorphous) [29].

#### 3.3.2. Parameters that Influence the Electrospraying Process

**Figure 10:**
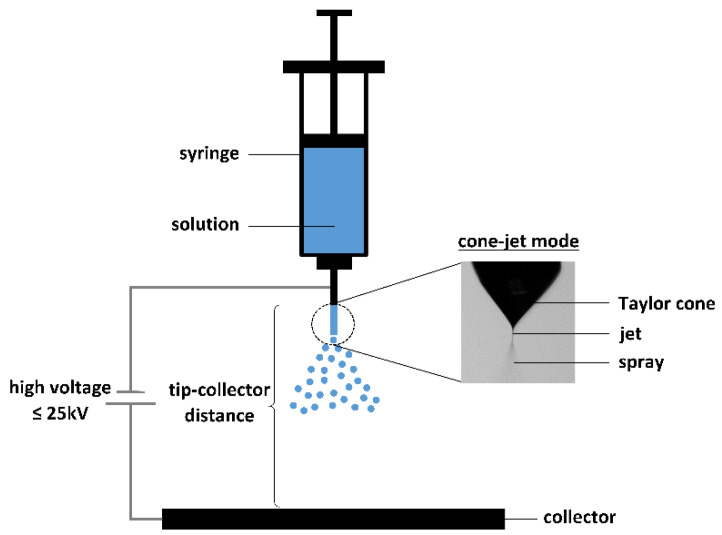
Schematic diagram of an electrospraying setup.

Over the last couple of decades, electrohydrodynamic atomization or electrospraying has gained extensive interest for the production of micro- and/or nanoparticulate materials in a wide variety of different fields and industries [190,191,192,193,194]. The relatively simple setup induces electric stress to a solution pumped through a nozzle by applying a high electric potential difference between the nozzle and collector, as visualized in Figure 10. Different functional modes arise depending on the process parameters and fluid characteristics, as described by Cloupeau et al. and classified by Jaworek et al. [195,196,197]. The dripping mode at relatively low voltages can be applied to produce droplets of similar size in regular time intervals [195]. This mode is most often denoted in the context of MDGs, and therefore already discussed (see Figure 3: EHDG in (micro)dripping mode). The size of the droplets and the frequency at which they fall are greatly dependent on the diameter of the capillary and the applied voltage [195,198]. Increasing the flow rate, the process transitions to the stable cone-jet mode that is most often applied for the generation of aerosols of small droplets, producing solid particles of the solute after rapid solvent evaporation. Hence, the main focus of this section will be on electrospraying in the cone-jet mode. In this mode, smaller and more monodisperse particles are obtained, compared to spray drying.

Electrospraying is a very attractive technology for solid particle generation, since it provides good control over the particle size and its distribution by the possibility to precisely manipulate the process parameters or solution properties [32]. Compared to spray drying, coalescence of droplets during their trajectory towards the collector is extremely limited due to the repelling surface charge of the droplets, adding to the quasi monodisperse final size distributions [171]. The absence of any additional energy source (e.g., gas streams) and the fact that electrospraying can be applied in ambient conditions for temperature and humidity make electrospraying a low specific power consuming process compared to similar techniques [199]. Moreover, the possibility to produce core-shell particles in a single step by utilizing a coaxial nozzle is a straightforward extension of the process. High encapsulation efficiencies with specifically engineered release characteristics are achievable, which is especially interesting for the pharmaceutical industry [32,200]. Despite the fact that quasi monodisperse low particle size products can be created, there still remain some challenges that need to be overcome before this manufacturing technique is applicable on an industrial production scale. In order to attain the most preferred cone-jet mode, low feed rates are applied, thereby severely limiting the throughput. However, the total liquid flow rate can be drastically increased via the application of multiple nozzles or multiplexed configurations [201,202,203,204]. Another disadvantage is that the constraints imposed on the physical properties of the liquid such as surface tension, conductivity, and volatility reduce the favorable design space compared to spray drying for example [5]. In the following section, the main process and formulation parameters that are specific to electrospraying (or different compared to the ones reviewed in the previous section of spray drying) will be discussed in detail.

##### Process Parameters

*Electrical Field Strength*

Identifying the stable cone-jet operating regime of electrospraying for a certain system is in most cases the first step in the generation of the desired particles. The two processing parameters that are most important in this regard are the electrical field strength, determined by the applied voltage and the tip to collector distance, and the flow rate of the liquid solution(s) [205]. Afterwards, further fine-tuning of these parameters allows for both morphology and size control of the final particles. Particle size and particle size distribution are important characteristics for the final performance of pharmaceutical formulations, since dissolution rate and thus release rates are strongly dependent on the surface to volume ratio [206]. Hence, the ability to control and adjust these properties of the final product make electrospraying a very attractive technique.

In general, a higher applied voltage in the stable cone-jet regime results in smaller final particles due to increased charge density on the jet and thus breakup into smaller emitted droplets [207,208,209,210]. Additionally, Coulomb fission (i.e., disintegration of shrinking droplets with too high surface charge (Rayleigh limit) into smaller drops with higher surface to volume ratios) occurs more rapidly since the Raleigh limit is reached earlier in the droplet lifetime. When electrospraying heptane, Tang et al. concluded that the effect of applied voltage on droplet size is more pronounced at higher flow rates [211]. The size reduction effect of higher applied voltages becomes minimal at low flow rates [212]. If the voltage is approaching the upper limit of the stable cone-jet regime, the emission of these fine droplets cause the polydispersity of the size distribution to increase [207,209]. Xu et al. observed the presence of tailed particles by electrospraying PLA in 1,2-dichloroethane at voltages close to the lower limit of cone-jet operation [213]. Songsurang et al. also observed irregular morphologies at both the lower and upper limit of stable cone-jet electrospraying, indicating that most likely some process instabilities lead to these altered morphologies [210].

The tip to collector distance determines the time of flight of the droplets, hence the available time for evaporation of the liquid. If adequately dried particles are required, sufficient distance between the nozzle tip and collector needs to be applied. For a constant voltage, shorter distances allow for higher electrical field intensities, thus smaller final particle sizes. Nevertheless, too short nozzle tip to collector distances can cause electric discharge at high voltages or insufficient solvent evaporation [32]. On the other hand, larger distances need larger applied voltages to generate the necessary electrical field strength, generally producing larger particles and a wider zone of particle deposition [194].

Flow Rate

The flow rate of the liquid solution is an important process parameter, influencing the stability of the process itself and the final particle size and morphology. The stable cone-jet mode is more easily obtained at lower flow rates [205]. Still, the flow rate cannot be too low, otherwise the cone-jet could solidify in case highly volatile solvents are applied, leading to nozzle blocking and/or discontinuous operation. The flow rate should be sufficiently low for complete solvent evaporation to occur. Otherwise, incomplete solvent evaporation could result in coalescence of particles at the collector or even film formation, similar to the effect of too low tip to collector distances.

Various authors have theoretically modeled cone-jet electrospraying and proposed scaling laws, which reveals that the emitted droplet diameter, which is directly related to the final particle size, depends on the flow rate Q [214,215,216]. According to Hartman et al., the type of breakup of the jet determines the main droplet diameter: $d_{drop}\sim \;Q^{0.48}$ for the varicose jet-breakup and $d_{drop}\sim \;Q^{0.33}$ in the whipping regime [214]. Hence, larger flow rates will result in larger final particle sizes, which has been experimentally found by many authors for various systems [208,209,217,218,219]. Furthermore, Jafari-Nadoushan et al. obtained more irregularly shaped PLGA particles at very low and high flow rates, compared to the spherical particles at intermediate flow rates [218]. Similarly, Enayati et al. observed increased polydispersity in particle size distribution at the upper and lower flow rate limit in the cone-jet operating mode [220]. The authors noticed an asymmetrical cone-jet formation at low flow rates and an increase in satellite droplets at high flow rates due to jet oscillation. Almeria et al. extended a simple model to accurately predict and control the morphology of polymer particles by calculating at which point in the droplet trajectory Coulomb fission will occur [221]. In their method, solution flow rate is the most important process parameter to control the morphology. They concluded that spherical particles are only produced if Coulomb fission can be suppressed, while more elongated and tailed particles will be generated otherwise [221].

Method of Collecting

Usually, the setup for collecting the particles produced by electrospraying is a hard surface located on the counter electrode. Nevertheless, different authors have published an alternative “soft” collecting approach where the droplets or particles are caught in liquid medium [222,223,224,225]. One of the benefits of this collecting method is the prevention of possible particle aggregation or film formation on the hard collector [223]. Gao et al. demonstrated that, by varying the collection medium, the particle morphology can be altered to obtain spherical, hemispherical and oblate shaped final particles [225]. Due to the difference in surface tension of the various collection liquids, the impaction force and thus degree of deformation changes, resulting in a variety of different particle shapes [225]. Another application of a liquid collector is described by Barron et al., who reviewed multiple applications of alginate-based microencapsulation by the coaxial electrospray method [224]. A coaxial nozzle allows for two different fluids to be electrosprayed, where the core fluid is encapsulated by the outer shell fluid. In this case, an alginate solution is found in the shell and the core is composed of an aqueous fluid containing living cells. When the alginate solution reaches the liquid collector containing a salt solution (e.g., CaCl_2_), immediate gelling of the alginate will take place, producing core-shell microcapsules with an alginate hydrogel shell [224]. Nikoo et al. utilized a similar alginate system, but operated in the dripping mode with a single nozzle for the generation of calcium alginate microhydrogels and investigated various process and formulation parameters on the final characteristics of the particles [226].

Environmental Conditions

The temperature at which electrospraying takes places determines the solvent evaporation rate of the droplets. The working temperature is generally in the range of 20–45 °C. The fact that this technique operates at such low temperatures, compared to spray drying, implies that heat-sensitive formulations can be processed. Nevertheless, the applied temperature should be sufficient to remove the solvent in the droplets during the time of flight and therefore depends on the volatility of the used solvent. In line with the Pe number discussion for SDD, fast solvent evaporation due to high temperatures or low volatile solvents generally cause a more wrinkled or collapsed morphology and an increase in porosity [227]. The solute diffusion is slow compared to the droplet drying rate, resulting in non-uniform solute distribution and localized phase separation, as reported by multiple authors [206,227,228,229]. In contrast, more dense spherical particles are produced if the diffusivity of the solute is high enough and sufficient time is given to minimize strong concentration gradients during solvent evaporation.

In addition to the general discussion of RH effects on droplet drying, for the electrospraying process the RH can strongly influence the surface porosity of the dry particles. This effect is shown by Ikeuchi et al., where the amount of water that is absorbed on the droplet containing PLA and ethanol greatly influences the surface porosity [230]. At lower RH, insufficient water gets absorbed, eliminating the phase separation and producing non-porous flattened or wrinkled particles. The authors indicate that the porosity of the particle allows for equilibration of the inner pressure in the droplet and the outer pressure, causing the spherical structure to stay intact instead of buckling or collapsing [230]. Similar results are found by multiple authors, including Bodnar et al. for ethylcellulose in methylethylketone and Zheng et al. for polystyrene/dimethylformamide solutions, indicating the important and often overlooked influence of humidity on the morphology and surface properties of the produced particles [199,231,232]. Consequently, since the environmental conditions during the process are of great importance to the final product properties, electrospraying is commonly performed inside a well-controlled environmental chamber.

##### Formulation Parameters

*Solute Concentration*

Following Equation (1), the initial concentration of solute determines to a large extent the size of the particles, but also the final morphology [9,233]. In general, larger and more spherical particles are more easily produced at higher solute concentrations. The concentration of polymers can however not be increased indefinitely, since the rapidly increasing viscosity can cause nozzle blocking or difficulties in operating in the stable cone-jet mode [228]. Furthermore, a transition to beads on fiber or even electrospinning of fibers can occur due to the elastic properties of highly concentrated polymer solutions preventing the complete breakup of the jet into small droplets [234,235]. Palangetic et al. have shown that the polymer molecular weight distribution greatly influences the minimal spinning concentration $c_{spin}$, especially the presence of high molecular weight fractions lower $c_{spin}$ drastically [236]. Srinivasan et al. have also experimentally shown the tunable morphology by adjusting the polymer concentration and the high molecular weight fraction [237]. An estimate of the highest polymer concentration for a certain polymer/solvent system that is electrosprayable without the formation of any fibers, can be found by performing viscosity measurements and determining the different concentration regimes, where the overlap concentration $c^{*}\;\sim \;1/\left[\eta \right]$ (where [η] is the intrinsic viscosity) and the entanglement concentration $c_{ent}$ form important limits [233,235,238,239]. Gupta et al. have shown that fiber formation can already occur at concentrations $1<c/c^{*}<3$ and only in the dilute regime $c<c^{*}$ solely particles are formed [238]. Not only the presence of entanglements but any form of network formation drastically increasing the viscoelastic properties of the solution results in the formation of fibers instead of only particles [234,240]. Shenoy et al. developed an alternative method, applicable for lower molecular weight polymers, for the prediction of the transition from electrospraying to spinning by evaluating the entanglement number in solution $n_{e,sol}$ [235]:

(25) $$n_{e,sol}=\frac{M_{w}}{M_{e,sol}}=\frac{\phi M_{w}}{M_{e}}$$

where $M_{w}$ is the weight average molecular weight, $M_{e}$ and $M_{e,sol}$ are the entanglement molecular weight in melt and in solution, respectively, and ϕ is the polymer volume fraction. The onset of chain overlap occurs when $n_{e,sol\;}=2$, which can be seen as an overlap concentration $c^{*}$ which equates to one entanglement per chain, while complete fiber formation occurs at $n_{e,sol}>3.5$ when a sufficiently strong elastic network is present.

As mentioned before, Coulomb fission at the Rayleigh limit due to increasing surface charge during droplet shrinkage can greatly influence the morphology and particle size distribution of the product. If the concentration of polymer in the falling droplet is high enough when this charge limit is reached, the viscoelastic properties of the droplet effectively suppress Coulomb fission, as reported by Festag et al. [241]. In the approach for morphology control by Almeria et al., the initial concentration or polymer volume fraction is therefore one of the most important formulation parameters [221]. A sufficiently high initial concentration will lead to a droplet that can resist Coulomb fission during its trajectory towards the collector, eliminating subdivision into smaller droplets and/or more irregular shaped morphologies.

The viscosity of the solution is strongly dependent on the solute concentration, especially for polymers. For a stable cone-jet operation, a sufficiently low viscosity is required to avoid blocking due to localized drying at the nozzle. Furthermore, higher voltages are necessary to induce more surface charge for the generation of a stable cone-jet, since they mainly counteract the surface tension but also the viscosity to obtain a Taylor cone. Jayasinghe et al. investigated the influence of solution viscosity on the final particle size while keeping the electrical conductivity constant by adding nitric acid [242]. They reported an emitted droplet and particle size increase with an increase in viscosity and a more polydisperse size distribution. This observation agrees with the earlier study of Rosell-Llompart et al., who experimentally studied key parameters and correlated them with the theoretical dimensionless study by De La Mora et al. [215,243].

Solvents

The choice of solvent is a crucial part of the feed formulation on which many process parameters and final particle properties depend. To deform a droplet at a nozzle tip into a Taylor cone, the surface charge needs to be sufficiently high to overcome the surface tension. Therefore, the surface tension will be one of the solution properties determining the strength of the applied electrical field to obtain stable cone-jet processing. Yao et al. added different concentrations of non-ionic surfactants to their hydrolyzed polyvinylalcohol/water solution which is by itself unable to be electrosprayed or spun [244]. Not only did the solution become electrosprayable by lowering the surface tension, electrospraying was prevalent at low surfactant concentrations while at higher concentrations electrospinning of fibers was observed. The authors do mention that other factors like localized gel formation most likely have an important influence on the cause of this transition [244]. Zhang et al. observed no considerable differences in final particle size, size distribution or morphology for the limited surface tension range they investigated [212]. The authors added different amounts of hexanol to chitosan/acetic acid solutions to only influence the surface tension without significantly altering conductivity or viscosity [212].

The volatility or boiling point of the solvent determines the environmental conditions as well as the required distance of the nozzle tip to collector to ensure complete solvent evaporation. Naturally, higher temperatures and a longer time of flight should be present for low volatile solvents and vice versa. As mentioned before, the solvent evaporation rate influences the particle morphology to a great extent. Xie et al. investigated different experimental setups for electrospraying of PLGA in acetonitrile and observed distinct morphologies depending on the setup [206]. Again in line with the Pe number discussion for SDD, the authors explain that the more irregular and collapsed particles are generated in the setup where the evaporation rate is inherently higher, while more spherical particles are produced if the evaporation rate is lower [206]. Less volatile solvents are often applied in a single or dual-solvent system for the production of more smooth and spherical particles [229]. Polymer chains have more time to diffuse and rearrange in the droplet, generating a more homogeneous surface and dense particle. In addition to volatility, the multiple other properties discussed for SDD as the total molecular diffusivity related to the molecular weight, solvent quality, polymer concentration, etc., determine the final morphology [245].

The electrical conductivity of the solution largely depends on the solvent if no ionic solutes are added. According to the scaling law of De La Mora et al., the emitted droplet diameter $d_{drop}$ is dependent on the conductivity K as $d_{drop}\;\sim \;{\left(\frac{1}{K}\right)}^{1/3}$ [215]. Smaller particles are observed at higher conductivities by many different authors [213,227,246]. Utilizing more conductive solvents or the addition of electrolytes are therefore effective ways to obtain smaller particles when necessary. However, solutions with lower electrical conductivity are generally more desirable because of more stable cone-jet operation and less rapid Coulomb fission. Meng et al. observed extremely unstable processing upon the addition of the highly conductive dimethylformamide to the PLGA/chloroform solution, creating polydisperse particles [247]. The addition of certain polymers or drug compounds can also drastically influence the electrical conductivity of the solution. Enayati et al. observed a fourfold decrease in the solution’s electrical conductivity with an increase from 2 wt% to 10 wt% of polycaprolactone in dimethylacetamide and a decrease in conductivity with the addition of a model drug β-oestradiol [220]. Smeets et al. noticed that most of the polymers relevant to the production of amorphous solid dispersions show a general trend of increasing solution conductivity with increasing concentration [205].

### 3.4. Experimental Characterization of the Spray Drying/Electrospraying Process

#### 3.4.1. Spray Characterization

In this section, studies performed on spray systems by means of the techniques reported in Section 2.3 are presented. Zhou and Cook used 2cLIF to determine solvent fractionation in an electrospray plume [248]. They utilized a solvatochromic dye (i.e., a dye that is sensitive to the polarity of the solvent) to distinguish between diverse solvents. In this way, they demonstrated the increase in concentration of the less volatile component during the drying process. The influence of different process parameters, such as capillary voltage and flow rate, on the evaporation rate was demonstrated as well [248]. Deprédurand et al. combined 2cLIF with PDA to perform spatially averaged temperature measurements and correlated these with the size of the droplets [249]. More specifically, they conducted droplet temperature measurements per droplet size class on n-decane droplets injected in an overheated air flow. From their study, they concluded that the smallest droplets had higher temperatures than the bigger ones. 2cLIF is also successfully employed to measure 2D temperature maps in sprays [249]. For example, Vetrano et al. were able to map the temperature of a liquid jet undergoing flash atomization [250]. Desantes et al. used LIEF to characterize the fuel concentration in both liquid and vapor phase of a Diesel spray in different engine conditions [251]. Using global rainbow refractometry, Wu et al. measured the concentration of binary droplets made of water and ethanol [252]. More specifically, the relative contributions of two different sprays (i.e., one existing out of water and one out of ethanol) to the final solution were determined. Likewise, Raman spectroscopy enables determining the chemical composition after mixing two aerosols with one another [253]. Although LIF is widely applied for temperature measurements in sprays, there is also some literature available on the use of Raman spectroscopy for temperature determination in sprays. This was, for example, performed by Vehring and Schweiger, who used Raman thermometry on water droplets [254]. As the OH-stretching band had a strong temperature dependence, it could be used for temperature evaluations. Additionally, Müller et al. exploited the shape and position of the OH-stretching band using Raman spectroscopy on ethanol and methanol sprays to determine their temperature distributions [255].

Next to temperature and composition, the velocity and size of droplets also need to be characterized to gain more insight in the drying process. PIV can be employed to characterize the velocity of drying droplets [256]. For instance, Poozesh et al. explored the atomization mechanism of an externally-mixed two-fluid nozzle spray dryer using PIV [257]. More specifically, they monitored the velocity fields in the near-field zone of the nozzle and the extent to which these were influenced by operational and formulation parameters [257]. Generally, PDA is more frequently applied to simultaneously measure the droplet velocity and size in spray systems. Liu et al. employed PDA to test the droplet velocities in nasal sprays [258]. By conducting a Box-Behnken design of experiments, they found that stroke length, actuation velocity, and concentration of both the surfactant and gelling agent used were determining factors for the velocities of the droplets [258]. Husted et al. compared PIV and PDA for their capabilities to explore droplet velocities in water mists [259]. They concluded that both methods are complementary, with PIV giving the instantaneous velocity field of the droplets and PDA showing the droplet velocity and size at a specific point [259]. Hence, it can be stated that PDA gives rise to 1D velocity measurements, while PIV allows the acquisition of 2D velocity measurements. Another emerging technique to simultaneously study droplet velocity and size is the time-shift method, also referred to as the pulse displacement technique [260,261].

As mentioned before, particle size distributions are important characteristics of the spray dried/electrosprayed powders. One aspect in gaining a deeper understanding of the final particle size distribution, is the evolution of droplet sizes during the drying process. Yang et al. performed in-line holographic measurements to evaluate the droplet size distributions during spray drying [262]. They explored the accuracy of the technique by adapting the region of the laser and the optimal location of the object relative to the laser beam [262]. Likewise, Na et al. investigated the performance of an aeroengine in which fuel atomization took place using a holographic measuring system [263]. This allowed them to capture an image of a hollow-cone shaped spray with most droplets concentrated at the surface of the cone [263]. As for dried particles, laser diffraction (LD) enables determination of the droplet size distribution. Mosén et al. performed LD to investigate both the droplet size distribution and the corresponding particle size distribution of spray dried mannitol particles [264]. In this way, they explored the effect of diverse process parameters, such as feed concentration and flow rate, on the sizes of the inhalable products [264]. Similarly, Littringer et al. showed the potential of LD to determine size distributions both after as during spray drying [170].

#### 3.4.2. Powder Characterization

The methods employed for solid-state characterization are not dependent on the preparation method, hence the same methods as for single particles are of interest. This section discusses examples of the aforementioned techniques on powders obtained via spray drying or electrospraying.

As for single dried particles, the morphology, size, and physical structure of dried powders impact their physicochemical properties. Regarding morphology, SEM, (cryo)-TEM, and CLSM can be applied. For instance, Patel et al. applied SEM analysis to prove that the particles they produced using their new IJSD were spherical and uniform [174]. Likewise, spray dried particles of HPMC and HPMCP were characterized using SEM by Vicente et al. [159]. Ku et al. determined the particle morphology and size distribution simultaneously by using a freezing method combined with TEM [265]. Freezing of the samples allowed them to analyze the samples in their original state [265]. CLSM showed to be effective for distinguishing the inner structure of particles prepared by spray drying from different carbohydrate solutions [158]. In addition to this, Wan et al. utilized X-ray photoelectron spectroscopy (XPS), earlier referred to as ESCA, to calculate the drug content at the surface of PLGA particles [184].

Another solid-state characteristic that can be explored is the particle size distribution. As already mentioned, this can be investigated using microscopic techniques and quantitative image analysis. Alternatively, LD can generate the particle size distribution. As an example, Clarke et al. used LD to explore the influence of process parameters on the characteristics of biodegradable polylactide and PLGA particles [185]. Among others, the particle size distribution was influenced by polymer molecular weight and polymer concentration. As mentioned before, Littringer et al. also relied on LD when establishing a correlation between droplet size and morphology of spray dried mannitol particles [170]. Even when investigating non-spherical particles, LD can be applied to determine their size as shown by Suh et al. [266].

A more in-depth physical characterization of the powder can also be conducted. Crystallinity is most commonly explored using XRD and (modulated) differential scanning calorimetry ((m)DSC). The latter also enables exploring the miscibility of the different components. For instance, Boel et al. employed the combination of XRD and mDSC to explore the potential of poly(2-ethyl-2-oxazoline) as a new carrier for ASDs, where mDSC enabled them to compare the miscibility of itraconazole with diverse polymers [267]. Additionally, in case of electrospraying, both analytical techniques are widely used, as can be seen in the earlier mentioned work from Smeets et al. [171]. It should be mentioned that there are much more characterization techniques available, for which the interested reader is referred to excellent reviews describing other methods for the evaluation of the crystallinity and miscibility of the system [268,269].

### 3.5. Modeling of the Drying Process during Spray Drying

#### 3.5.1. Atomization and Size Distribution Models

Several atomization models have been developed and their main aim is to predict droplet diameters as a function of controllable parameters. Numerous correlations are available in literature, where, most often, mean droplet diameters are predicted as a function of the atomizing gas velocity [270]. Additionally, more advanced models exist that link the droplet diameter to the atomizing gas velocity and the liquid surface tension, considering liquid instabilities inside the nozzle that lead to the formation of a multitude of smaller droplets [270]. Despite their high predictive value, the aforementioned models are constructed for two-fluid nozzles with a particular geometry, so one must be cautious when extrapolating to other geometries and/or different nozzle types [159]. Because of the strong relation between droplet size and particle size, atomization models can be used for controlling particle size during the spray drying process. In addition to droplet diameter predictive models, other models are available to obtain the whole droplet size distribution via CFD simulations. Most of them rely on the maximum entropy principle (MEP) or on extended versions of it, which allow the interactions between the sprayed liquid and the drying gas [271].

#### 3.5.2. Spray Drying Modeling

Kemp and Oakley proposed five distinct complexity levels to classify drying models [161,272]. The first level is limited to heat and mass balance and does not consider the impact of mass flow rates, moisture content and gas humidity. This level is not sufficient to predict the spray dryer performance and size, nor to predict the behavior of the individual particles.

The second level, characterized by the estimation of dryer size and drying time (so mass flow rates), consists in the coupling of the constant-rate evaporation model and the first-order falling rate model (i.e., the so-called scoping calculations), which can be linked to the different drying stages depicted in Figure 1. Heat transfer coefficients and falling rate drying kinetics cannot be predicted in this case. The constant-rate evaporation model can be described by the following equations.

(26) $$d^{2}\left(t\right)=d_{0}^{2}-\kappa \;t$$

(27) $$\tau_{D}=\frac{d_{0}^{2}}{\kappa }$$

where d(t) is the droplet diameter at timepoint t, $d_{0}$ is the initial droplet diameter, t is the time, κ the evaporation rate constant, and $\tau_{D}$ is the characteristic droplet drying time. This model also comprises the well-known and commonly used Pe number by making use of the evaporation rate constant and the diffusion coefficient.

As mentioned before, hollow particles are anticipated if Pe > 1 whilst dense solid particles are obtained for Pe ≤ 1. Vehring et al. discussed that the constant-rate model is assuming constant gas and droplet temperatures, indicating its inappropriateness for the non-stationary evaporation both at an early stage and during solidification [3]. Moreover, mass diffusivity should also be dependent upon the concentration of the solutes [3,118]. The constant evaporation model only applies to relatively simple systems, covering single solvent/solute systems, thereby hampering its predictive capacity for spray drying processes. If multiple solvents/solutes are used, the solutes can have different solubilities and diffusion coefficients in the different solvents, which of course makes the drying process more complex [184]. Nonetheless, the models belonging to the second level are often used for an initial, yet general, characterization: for example, relating particle size to droplet size and concentration, predicting droplet size distributions within a spray or predicting minimum droplet evaporation times [158,161].

The third level includes the use of scaling calculations which provide, as its name suggests, performance outcomes and dimensions by scaling up measured drying curves from small-scale or pilot-plant experiments. Local variations inside the dryer are out of scope, which means that up to this third level, the spray dryer is still considered as a whole. More detailed methods therefore aim at simulating local conditions inside the spray dryer.

One-dimensional level four models track local conditions of both particles and drying gas during the spray drying process, whilst level five models (better known as CFD) predict complex three-dimensional flow patterns and heat and mass transfer, evaporation and solidification processes within an individual droplet, thereby making CFD a powerful tool for designing spray dryers and optimizing process conditions [161]. The fourth level can be exemplified by the gSOLIDS program that calculates drying rates and moisture contents of all droplets via the discretization of the droplet size distribution, provided that this distribution is known beforehand [273]. However, a number of assumptions need to be made here. The drying process after crust formation follows first-order kinetics, that, according to Kemp et al., does not fully predict the behavior in a spray dryer where particle residence times are in the millisecond range [161]. If crust formation occurs, drying is much slower than first-order kinetics since the liquid inside has to diffuse through the crust to escape (i.e., either as liquid or vapor). Recently, a modified Oakley model was implemented in the gSOLIDS program to take this effect into account. Likewise, Handscomb et al. developed a new model allowing continuous shrinkage of the droplet, even after crust formation occurred [161].

A discretization principle is also used in CFD simulations, for example by Ullum et al. [152]. Those researchers performed CFD modeling based on the Eulerian-Lagrangian approach to determine the flow and deposits on the drying chamber wall in an industrial spray dryer, equipped with one pressure nozzle. The 2D Eulerian approach treats both droplet phase and gas flow as interpenetrating continua. The 3D Lagrangian calculation models the spray drying process as continue gas flow containing discrete droplet parcels, where each parcel encompasses droplets of similar size [272]. Although a good prediction of the particle temperature history was achieved, phenomena like coalescence and agglomeration were not considered [152]. Kemp et al. also presented CFD simulations of the air-flow patterns based on the Lagrangian approach without including agglomeration and coalescence, but for a laboratory spray dryer with a two-fluid nozzle [161]. Nijdam et al. developed both a Lagrangian-based and a Eulerian-based CFD model to predict the turbulent dispersion and coalescence within a spray [6]. It is important to underline that the concept of coalescence must be differentiated from the concept of agglomeration. The former occurs for liquid droplets whilst agglomeration takes place for moist particles, when sufficient free moisture has already been evaporated. When modeling agglomeration of moist particles, temperature and evaporation effects are crucial to consider. This is not the case when modeling coalescence, since in that case, evaporation is negligible [6]. Noteworthy, models that account for many of the flow phenomena during spray drying, especially those related to turbulence, are continuously subject to further developments.

## 4. Conclusions

**Figure 11:**
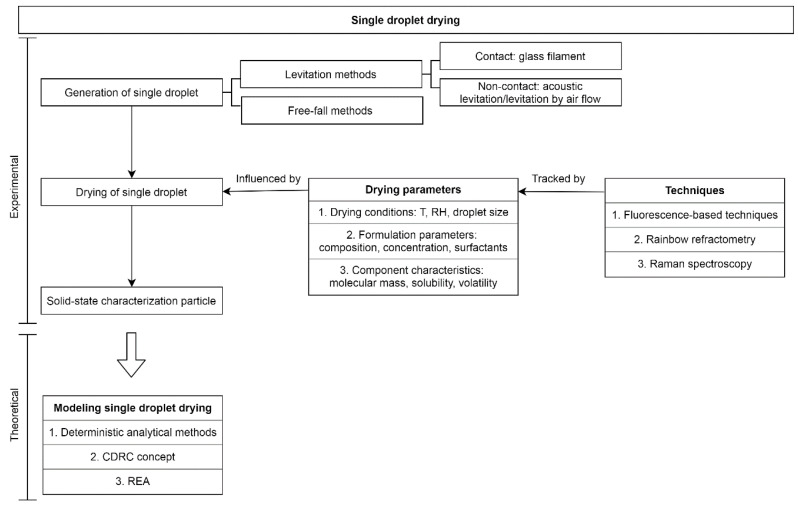
Flow chart single droplet drying process.

**Figure 12:**
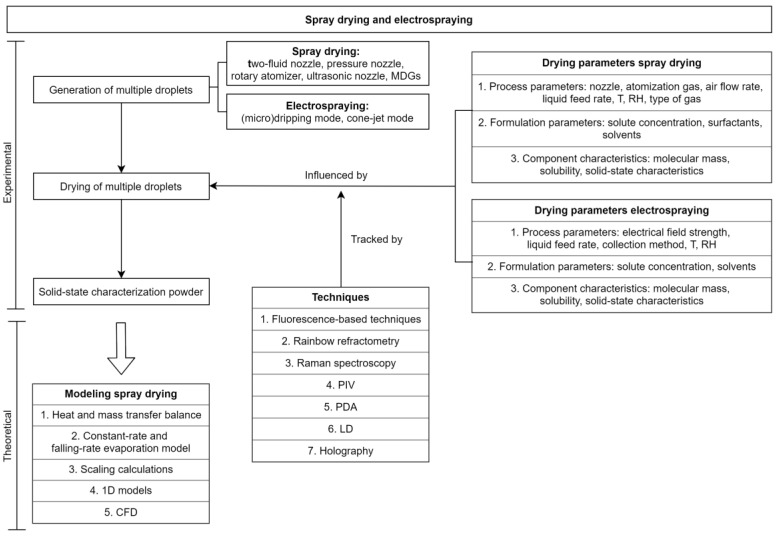
Flow chart spray drying and electrospraying.

The focus of this review article lies on a deeper understanding of the droplet drying process to facilitate particle engineering. The review is therefore divided into a single droplet drying part and a pharmaceutical application part, both of which are illustrated in a flow chart in Figure 11 and Figure 12, respectively. Overall, the findings presented in this article have strengthened the predictive value of single droplet drying for pharmaceutical drying applications like spray drying and electrospraying. After a profound literature review, the same trends are observed regarding the impact of drying parameters on particle size and morphology, both key influencing factors for the bulk characteristics of the resulting powder. Continuous follow-up of the particle formation process in single droplet drying experiments hence allows optimization of manufacturing processes and particle engineering approaches such as spray drying and electrospraying and acceleration of process development. Single droplet drying is therefore considered indispensable to fill the gap of knowledge that still exists regarding the particle formation process in pharmaceutical drying applications. Nevertheless, correct interpretations can only be made if the differences in drying conditions between single droplet drying and spray drying/electrospraying are recognized.

## Figures and Tables

**Table 1:** Comparison of single droplet drying methods: Principles, information, (dis)advantage(s), and similarity to pharmaceutical applications [1,15,17].

Single Droplet Drying Method	Glass Filament	Acoustic Levitation	Levitation by Air Flow	Free-Fall
**Principle**	Suspension of droplet on tip of glass filament/capillary	Levitation of droplet in an acoustic field	Levitation of droplet by air flow	Droplets falling through a drying chamber
**Information**	- Mass - Temperature - Diameter - Radial composition	- Temperature - Diameter - Radial composition	- Temperature - Diameter - Radial composition	Only indirect measurements
**Advantage(s)**	- All parameters can be measured simultaneously - Relatively simple measurement of parameters possible - Can be extended for in situ dissolution testing	Relatively simple measurement of parameters possible	Relatively simple measurement of parameters possible	Resembles drying process in a spray dryer (and in electrospraying)
**Disadvantage(s)**	- Intrusive technique: small effect of filament on heat transfer and morphology - Positioning of the droplet	- Acoustic waves can influence heat transfer and shape of the droplet - No direct mass measurement - Droplet deformation possible - Positioning of the droplet	- No direct mass measurement - Positioning of the droplet	Complex measurement of parameters: impossible to continuously track the drying process
**Similarity to pharmaceutical applications**	- Interference of drying by intrusive glass filament - Bigger droplet size (500 µm–9 mm)	- Interference of drying by acoustic waves - Bigger droplet size (300 µm–1 mm)	Not often applied in pharmaceutical industry	Very similar to spray drying (and electrospraying)

**Table 2:** Different drying parameters and droplet characterization techniques.

**During Drying**	
**Parameter**	**Technique**
Temperature	LIF Rainbow refractometry Raman spectroscopy Thermographic phosphors Thermochromic liquid crystal infrared thermometry Infrared thermometry
Composition	LIF Rainbow refractometry Raman spectroscopy NMR
Droplet size	PDA Holography LD
Velocity	PIV PDA
Vapor concentration	LIF
**After drying**	
**Parameter**	**Technique**
Morphology	SEM (cryo)-TEM CLSM
Particle size	SEM (cryo)-TEM LD
Physical structure	XRD

## References

[1] De Souza Lima R., Ré M.I., Arlabosse P. (2020). Drying droplet as a template for solid formation: A review. Powder Technol..

[2] Nandiyanto A.B.D., Okuyama K. (2011). Progress in developing spray-drying methods for the production of controlled morphology particles: From the nanometer to submicrometer size ranges. Adv. Powder Technol..

[3] Vehring R., Foss W.R., Lechuga-Ballesteros D. (2007). Particle formation in spray drying. J. Aerosol Sci..

[4] Mujumdar A.S., Huang L.X. (2007). Global R&D needs in drying. Dry. Technol..

[5] Singh A., van den Mooter G. (2016). Spray drying formulation of amorphous solid dispersions. Adv. Drug Deliv. Rev..

[6] Nijdam J.J., Guo B., Fletcher D.F., Langrish T.A.G. (2004). Challenges of simulating droplet coalescence within a spray. Dry. Technol..

[7] Farid M. (2003). A new approach to modelling of single droplet drying. Chem. Eng. Sci..

[8] Nešić S., Vodnik J. (1991). Kinetics of droplet evaporation. Chem. Eng. Sci..

[9] Vehring R. (2008). Pharmaceutical particle engineering via spray drying. Pharm. Res..

[10] Mezhericher M., Levy A., Borde I. (2011). Modelling the morphological evolution of nanosuspension droplet in constant-rate drying stage. Chem. Eng. Sci..

[11] Mezhericher M., Levy A., Borde I. (2008). Modelling of particle breakage during drying. Chem. Eng. Process..

[12] Fox B., Bellini G., Pellegrini L., Vogel H.C., Todaro C.M. (2014). Drying. Fermentation and Biochemical Engineering Handbook.

[13] Mezhericher M., Levy A., Borde I. (2010). Spray drying modelling based on advanced droplet drying kinetics. Chem. Eng. Process. Process. Intensif..

[14] Sadek C., Schuck P., Fallourd Y., Pradeau N., Le Floch-Fouéré C., Jeantet R. (2015). Drying of a single droplet to investigate process–structure–function relationships: A review. Dairy Sci. Technol..

[15] Fu N., Woo M.W., Chen X.D. (2012). Single droplet drying technique to study drying kinetics measurement and particle functionality: A review. Dry. Technol..

[16] Adhikari B., Howes T., Bhandari B.R., Truong V. (2000). Experimental studies and kinetics of single drop drying and their relevance in drying of sugar-rich foods: A review. Int. J. Food Prop..

[17] Schutyser M.A.I., Both E.M., Siemons I., Vaessen E.M.J., Zhang L. (2019). Gaining insight on spray drying behavior of foods via single droplet drying analyses. Dry. Technol..

[18] Han K., Song G., Ma X., Yang B. (2016). An experimental and theoretical study of the effect of suspended thermocouple on the single droplet evaporation. Appl. Therm. Eng..

[19] Lin S.X.Q., Chen X.D. (2002). Improving the glass-filament method for accurate measurement of drying kinetics of liquid droplets. Chem. Eng. Res. Des..

[20] Lin J.C., Gentry J.W. (2003). Spray drying drop morphology: Experimental study. Aerosol Sci. Technol..

[21] Fu N., Wu W.D., Yu M., Moo F.T., Woo M.W., Selomulya C., Chen X.D. (2016). In situ observation on particle formation process via single droplet drying apparatus: Effects of precursor composition on particle morphology. Dry. Technol..

[22] Fu N., Woo M.W., Moo F.T., Chen X.D. (2012). Microcrystallization of lactose during droplet drying and its effect on the property of the dried particle. Chem. Eng. Res. Des..

[23] Ali Al Zaitone B., Tropea C. (2011). Evaporation of pure liquid droplets: Comparison of droplet evaporation in an acoustic field versus glass-filament. Chem. Eng. Sci..

[24] Groenewold C., Möser C., Groenewold H., Tsotsas E. (2002). Determination of single-particle drying kinetics in an acoustic levitator. Chem. Eng. J..

[25] Griesing M., Grosshans H., Hellwig T., Sedelmayer R., Gopireddy S.R., Pauer W., Gutheil E., Moritz H.U. (2016). Influence of air humidity on the particle formation of single mannitol-water droplets during drying. Chem. Ing. Technik.

[26] Andrade M.A.B., Pérez N., Adamowski J.C. (2018). Review of progress in acoustic levitation. Braz. J. Phys..

[27] Mondragon R., Hernandez L., Enrique Julia J., Carlos Jarque J., Chiva S., Zaitone B., Tropea C. (2011). Study of the drying behavior of high load multiphase droplets in an acoustic levitator at high temperature conditions. Chem. Eng. Sci..

[28] Saha A., Basu S., Kumar R. (2012). Velocity and rotation measurements in acoustically levitated droplets. Phys. Lett. Sect. A Gen. At. Solid State Phys..

[29] Wulsten E., Kiekens F., van Dycke F., Voorspoels J., Lee G. (2009). Levitated single-droplet drying: Case study with itraconazole dried in binary organic solvent mixtures. Int. J. Pharm..

[30] Duo Wu W., Patel K.C., Rogers S., Chen X.D. (2007). Monodisperse droplet generators as potential atomizers for spray drying technology. Dry. Technol..

[31] Liu W., Chen X.D., Selomulya C. (2015). On the spray drying of uniform functional microparticles. Particuology.

[32] Nguyen D.N., Clasen C., Van den Mooter G. (2016). Pharmaceutical applications of electrospraying. J. Pharm. Sci..

[33] Udey R.N., Jones A.D., Farquar G.R. (2013). Aerosol and microparticle generation using a commercial inkjet printer. Aerosol Sci. Technol..

[34] Duo Wu W., Amelia R., Hao N., Selomulya C., Zhao D., Chiu Y.-L., Chen X.D. (2011). Assembly of Uniform Photoluminescent Microcomposites Using a Novel Micro-Fluidic-Jet-Spray-Dryer. Am. Inst. Chem. Eng. AIChE J..

[35] Duo Wu W., Lin S.X., Chen X.D. (2011). Monodisperse Droplet Formation Through a Continuous Jet Break-Up Using Glass Nozzles Operated with Piezoelectric Pulsation. AIChE J..

[36] Liu W., Duo Wu W., Selomulya C., Chen X.D. (2011). Uniform Chitosan Microparticles Prepared by a Novel Spray-Drying Technique. Int. J. Chem. Eng..

[37] Baldelli A., Boraey M.A., Nobes D.S., Vehring R. (2015). Analysis of the Particle Formation Process of Structured Microparticles. Mol. Pharm..

[38] Rogers S., Fang Y., Qi Lin S.X., Selomulya C., Dong Chen X. (2012). A monodisperse spray dryer for milk powder: Modelling the formation of insoluble material. Chem. Eng. Sci..

[39] Fang Y., Rogers S., Selomulya C., Chen X.D. (2012). Functionality of milk protein concentrate: Effect of spray drying temperature. Biochem. Eng. J..

[40] Amelia R., Wu W.D., Cashion J., Bao P., Zheng R., Chen X.D., Selomulya C. (2011). Microfluidic spray drying as a versatile assembly route of functional particles. Chem. Eng. Sci..

[41] Liu W., Wu W., Selomulya C., Chena X.D. (2011). A single step assembly of uniform microparticles for controlled release applications. Soft Matter.

[42] Maa Y.F., Nguyen P.A.T., Hsu S.W. (1998). Spray-drying of air-liquid interface sensitive recombinant human growth hormone. J. Pharm. Sci..

[43] Wang S., Langrish T. (2009). A review of process simulations and the use of additives in spray drying. Food Res. Int..

[44] Charlesworth D.H., Marshall W.R. (1960). Evaporation from drops containing dissolved solids. AIChE J..

[45] Grosshans H., Griesing M., Mönckedieck M., Hellwig T., Walther B., Gopireddy S.R., Sedelmayer R., Pauer W., Moritz H.U., Urbanetz N.A. (2016). Numerical and experimental study of the drying of bi-component droplets under various drying conditions. Int. J. Heat Mass Transf..

[46] Grosshans H., Griesing M., Hellwig T., Pauer W., Moritz H.U., Gutheil E. (2016). A new model for the drying of mannitol-water droplets in hot air above the boiling temperature. Powder Technol..

[47] Tran T.T.H., Avila-Acevedo J.G., Tsotsas E. (2016). Enhanced methods for experimental investigation of single droplet drying kinetics and application to lactose/water. Dry. Technol..

[48] Tran T.T.H., Jaskulski M., Avila-Acevedo J.G., Tsotsas E. (2017). Model parameters for single-droplet drying of skim milk and its constituents at moderate and elevated temperatures. Dry. Technol..

[49] Fu N., Woo M.W., Selomulya C., Chen X.D. (2013). Shrinkage behaviour of skim milk droplets during air drying. J. Food Eng..

[50] Gouaou I., Shamaei S., Koutchoukali M.S., Bouhelassa M., Tsotsas E., Kharaghani A. (2019). Impact of operating conditions on a single droplet and spray drying of hydroxypropylated pea starch: Process performance and final powder properties. Asia Pac. J. Chem. Eng..

[51] Al Zaitone B., Lamprecht A. (2013). Single droplet drying step characterization in microsphere preparation. Colloids Surf. B Biointerfaces.

[52] Al Zaitone B., Al-Zahrani A., Al-Shahrani S., Lamprecht A. (2020). Drying of a single droplet of dextrin: Drying kinetics modeling and particle formation. Int. J. Pharm..

[53] Har C.L., Fu N., Chan E.S., Tey B.T., Chen X.D. (2018). In situ crystallization kinetics and behavior of mannitol during droplet drying. Chem. Eng. J..

[54] Fu N., Yu M., Chen X.D. (2019). A differential shrinkage approach for evaluating particle formation behavior during drying of sucrose, lactose, mannitol, skim milk, and other solid-containing droplets. Dry. Technol..

[55] Schiffter H., Lee G. (2012). Single-Droplet Evaporation Kinetics and Particle Formation in an Acoustic Levitator. Part 2: Drying Kinetics and Particle Formation from Microdroplets of Aqueous Mannitol, Trehalose, or Catalase. J. Pharm. Sci..

[56] Walton D.E., Mumford C.J. (1999). The morphology of spray-dried particles. The effect of process variables upon the morphology of spray-dried particles. Chem. Eng. Res. Des..

[57] Gregson F.K.A., Robinson J.F., Miles R.E.H., Royall C.P., Reid J.P. (2019). Drying Kinetics of Salt Solution Droplets: Water Evaporation Rates and Crystallization. J. Phys. Chem. B.

[58] Sedelmayer R., Griesing M., Halfar A.H., Pauer W., Moritz H.U. (2013). Experimental investigation of the morphology formation of polymer particles in an acoustic levitator. Macromol. Symp..

[59] Fritsching U. (2016). Process.-Spray: Functional Particles Produced in Spray Processes.

[60] Rapp B.E. (2017). Microfluidics: Modeling, Mechanics and Mathematics.

[61] Zang D., Tarafdar S., Tarasevich Y.Y., Dutta Choudhury M., Dutta T. (2019). Evaporation of a droplet: From physics to applications. Phys. Rep..

[62] Bahadur J., Sen D., Mazumder S., Bhattacharya S., Frielinghaus H., Goerigk G. (2011). Origin of buckling phenomenon during drying of micrometer-sized colloidal droplets. Langmuir.

[63] Walton D.E. (2000). The morphology of spray-dried particles: A qualitative view. Dry. Technol..

[64] Okuzono T., Ozawa K., Doi M. (2006). Simple model of skin formation caused by solvent evaporation in polymer solutions. Phys. Rev. Lett..

[65] Osman A., Shahidzadeh N., Stitt H., Shokri N. (2018). Morphological transformations during drying of surfactant-nanofluid droplets. J. Ind. Eng. Chem..

[66] Adler M., Unger M., Lee G. (2000). Surface composition of spray-dried particles of bovine serum albumin/trehalose/surfactant. Pharm. Res..

[67] Nuzzo M., Millqvist-Fureby A., Sloth J., Bergenstahl B. (2015). Surface composition and morphology of particles dried individually and by spray drying. Dry. Technol..

[68] Fäldt P., Bergenståhl B. (1994). The surface composition of spray-dried protein-lactose powders. Colloids Surf. A Physicochem. Eng. Asp..

[69] Meerdink G., van’t Riet K. (1995). Modeling segregation of solute material during drying of liquid foods. AIChE J..

[70] Tsotsas E., Mujumdar A.S., Tsotsas E., Mujumdar A.S. (2014). Front Matter, Volume 3: Product Quality and Formulation. Modern Drying Technology.

[71] Poozesh S., Bilgili E. (2019). Scale-up of pharmaceutical spray drying using scale-up rules: A review. Int. J. Pharm..

[72] Raula J., Eerikäinen H., Kauppinen E.I. (2004). Influence of the solvent composition on the aerosol synthesis of pharmaceutical polymer nanoparticles. Int. J. Pharm..

[73] Gregson F.K.A., Ordoubadi M., Miles R.E.H., Haddrell A.E., Barona D., Lewis D., Church T., Vehring R., Reid J.P. (2019). Studies of competing evaporation rates of multiple volatile components from a single binary-component aerosol droplet. Phys. Chem. Chem. Phys..

[74] Lemoine F., Castanet G. (2013). Temperature and chemical composition of droplets by optical measurement techniques: A state-of-the-art review. Exp. Fluids.

[75] Gouesbet G., Gréhan G. (2015). Laser-based optical measurement techniques of discrete particles: A review [invited keynote]. Int. J. Multiph. Flow.

[76] Schulz C., Sick V. (2005). Tracer-LIF diagnostics: Quantitative measurement of fuel concentration, temperature and fuel/air ratio in practical combustion systems. Prog. Energy Combust. Sci..

[77] Maqua C., Depredurand V., Castanet G., Wolff M., Lemoine F. (2007). Composition measurement of bicomponent droplets using laser-induced fluorescence of acetone. Exp. Fluids.

[78] Shringi D.S., Shaw B.D., Dwyer H.A. (2009). Laser-induced fluorescence imaging of acetone inside evaporating and burning fuel droplets. Opt. Lasers Eng..

[79] Mercier X., Orain M., Grisch F. (2007). Investigation of droplet combustion in strained counterflow diffusion flames using planar laser-induced fluorescence. Appl. Phys. B Lasers Opt..

[80] Lavieille P., Lemoine F., Lavergne G., Lebouché M. (2001). Evaporating and combusting droplet temperature measurements using two-color laser-induced fluorescence. Exp. Fluids.

[81] Lavieille P., Lemoine F., Lavergne G., Virepinte J.F., Lebouché M. (2000). Temperature measurements on droplets in monodisperse stream using laser-induced fluorescence. Exp. Fluids.

[82] Castanet G., Labergue A., Lemoine F. (2011). Internal temperature distributions of interacting and vaporizing droplets. Int. J. Therm. Sci..

[83] Maqua C., Castanet G., Lemoine F., Doué N., Lavergne G. (2006). Temperature measurements of binary droplets using three-color laser-induced fluorescence. Exp. Fluids.

[84] Fansler T.D., Parrish S.E. (2015). Spray measurement technology: A review. Meas. Sci. Technol..

[85] Zhang Y., Zhang G., Xu M., Wang J. (2013). Droplet temperature measurement based on 2-color laser-induced exciplex fluorescence. Exp. Fluids.

[86] Zeng Y., Jiang L., Zheng W., Li D., Yao S., Qu J.Y. (2011). Quantitative imaging of mixing dynamics in microfluidic droplets using two-photon fluorescence lifetime imaging. Opt. Lett..

[87] Omrane A., Santesson S., Aldén M., Nilsson S. (2004). Laser techniques in acoustically levitated micro droplets. Lab. Chip.

[88] Omrane A., Särner G., Aldén M. (2004). 2D-temperature imaging of single droplets and sprays using thermographic phosphors. Appl. Phys. B Lasers Opt..

[89] Promvongsa J., Fungtammasan B., Gerard G., Saengkaew S., Vallikul P. (2017). A Study on the Evaporation of Water – Ethanol Mixture Using Rainbow Refractometry. J. Energy Resour. Technol..

[90] Li H., Rosebrock C.D., Wriedt T., Mädler L. (2017). Journal of Quantitative Spectroscopy & Radiative Transfer The effect of initial diameter on rainbow positions and temperature distributions of burning single-component n-Alkane droplets. J. Quant. Spectrosc. Radiat. Transf..

[91] Vetrano M.R., Petrus J., Johannes A., Riethmuller M.L. (2005). Nonuniform spheres. Opt. Lett..

[92] Wu Y., Crua C., Li H., Saengkaew S., Mädler L., Wu X., Gréhan G. (2018). Simultaneous measurement of monocomponent droplet temperature/refractive index, size and evaporation rate with phase rainbow refractometry. J. Quant. Spectrosc. Radiat. Transf..

[93] Ingchun Y.W.U., Romvongsa J.A.P., Aengkaew S.A.S., Uecheng X.W.U., Hen J.I.A.C., Réhan G.É.G. (2016). Phase rainbow refractometry for accurate droplet variation characterization. Opt. Lett..

[94] Albrecht H.E., Borys M., Damasche N., Tropea C. (2003). Laser Doppler and Phase Doppler Measurement Techniques.

[95] Zhao Y., Qiu H.H. (2006). Measurements of multicomponent microdroplet evaporation by using rainbow refractometer and PDA. Exp. Fluids.

[96] Van Beeck J.P.A.J., Giannoulis D., Zimmer L., Riethmuller M.L. (1999). Global rainbow thermometry for droplet-temperature measurement. Opt. Lett..

[97] Van Beeck J.P.A.J., Zimmer L., Riethmuller M.L. (2001). Global rainbow thermometry for mean temperature and size measurement of spray droplets. Part. Part. Syst. Charact..

[98] Schweiger G. (1990). Raman scattering on single aerosol particles and on flowing aerosols: A review. J. Aerosol Sci..

[99] Vehring R., Aardahl C.L., Schweiger G., Davis E.J. (1998). The characterization of fine particles originating from an uncharged aerosol: Size dependence and detection limits for Raman analysis. J. Aerosol Sci..

[100] Moritz H., Lange S., Schweiger G. (1997). The radial weighting of concentration profiles inside of microparticles by Raman spectroscopy. J. Aerosol Sci..

[101] Suzuki H., Matsuzaki Y., Muraoka A., Tachikawa M. (2012). Raman spectroscopy of optically levitated supercooled water droplet. J. Chem. Phys..

[102] Heinisch C., Wills J.B., Reid J.P., Tschudi T., Tropea C. (2009). Temperature measurement of single evaporating water droplets in a nitrogen flow using spontaneous raman scattering. Phys. Chem. Chem. Phys..

[103] Reid J.P., Meresman H., Mitchem L., Symes R. (2007). Spectroscopic studies of the size and composition of single aerosol droplets. Int. Rev. Phys. Chem..

[104] Hopkins R.J., Reid J.P. (2005). Evaporation of ethanol/water droplets: Examining the temporal evolution of droplet size, composition and temperature. J. Phys. Chem. A.

[105] Kihara Y., Asami H., Kohno J.Y. (2017). Evaporation and Subsequent Adsorption of Alcohol Molecules at Aqueous Droplet Surface Observed by Cavity-Enhanced Raman Spectroscopy. J. Phys. Chem. B.

[106] Richards C.D., Richards R.F. (1998). Transient temperature measurements in a convectively cooled droplet. Exp. Fluids.

[107] Griffith J.D., Bayly A.E., Johns M.L. (2008). Magnetic resonance studies of detergent drop drying. Chem. Eng. Sci..

[108] Foerster M., Gengenbach T., Wai M., Selomulya C. (2016). The influence of the chemical surface composition on the drying process of milk droplets. Adv. Powder Technol..

[109] Chen X.D. (2008). The basics of a reaction engineering approach to modeling air-drying of small droplets or thin-layer materials. Dry. Technol..

[110] Mezhericher M., Levy A., Borde I. (2010). Theoretical models of single droplet drying kinetics: A review. Dry. Technol..

[111] Ranz W.E., Marshall W. (1952). Evaporation from drops. Chem. Eng. Prog..

[112] Peishi C., Pei D.C.T. (1989). A mathematical model of drying processes. Int. J. Heat Mass Transf..

[113] Cheong H.W., Jeffreys G.V., Mumford C.J. (1986). A receding interface model for the drying of slurry droplets. AIChE J..

[114] Mezhericher M., Levy A., Borde I. (2007). Theoretical drying model of single droplets containing insoluble or dissolved solids. Dry. Technol..

[115] Mezhericher M., Levy A., Borde I. (2008). Heat and mass transfer of single droplet/wet particle drying. Chem. Eng. Sci..

[116] Gac J.M., Gradoń L. (2013). A distributed parameter model for the spray drying of multicomponent droplets with a crust formation. Adv. Powder Technol..

[117] Wang S., Langrish T.A.G. (2009). A distributed parameter model for particles in the spray drying process. Adv. Powder Technol..

[118] Chen X.D., Sidhu H., Nelson M. (2011). Theoretical probing of the phenomenon of the formation of the outermost surface layer of a multi-component particle, and the surface chemical composition after the rapid removal of water in spray drying. Chem. Eng. Sci..

[119] Xiao J., Chen X.D. (2014). Multiscale modeling for surface composition of spray-dried two-component powders. AIChE J..

[120] Xiao J., Chen L., Wu W.D., Chen X.D. (2016). Multiscale modeling for nanoscale surface composition of spray-dried powders: The effect of initial droplet size. Dry. Technol..

[121] Parienta D., Morawska L., Johnson G.R., Ristovski Z.D., Hargreaves M., Mengersen K., Corbett S., Chao C.Y.H., Li Y., Katoshevski D. (2011). Theoretical analysis of the motion and evaporation of exhaled respiratory droplets of mixed composition. J. Aerosol Sci..

[122] Seydel P., Blömer J., Bertling J. (2006). Modeling particle formation at spray drying using population balances. Dry. Technol..

[123] Grasmeijer N., Frijlink H.W., Hinrichs W.L.J. (2016). Model to predict inhomogeneous protein–sugar distribution in powders prepared by spray drying. J. Aerosol Sci..

[124] Werner S.R.L., Edmonds R.L., Jones J.R., Bronlund J.E., Paterson A.H.J. (2008). Single droplet drying: Transition from the effective diffusion model to a modified receding interface model. Powder Technol..

[125] Gopireddy S.R., Gutheil E. (2013). Numerical simulation of evaporation and drying of a bi-component droplet. Int. J. Heat Mass Transf..

[126] Sazhin S.S., Rybdylova O., Pannala A.S., Somavarapu S., Zaripov S.K. (2018). A new model for a drying droplet. Int. J. Heat Mass Transf..

[127] Sadafi M.H., Jahn I., Stilgoe A.B., Hooman K. (2014). Theoretical and experimental studies on a solid containing water droplet. Int. J. Heat Mass Transf..

[128] Sadafi M.H., Jahn I., Stilgoe A.B., Hooman K. (2015). A theoretical model with experimental verification for heat and mass transfer of saline water droplets. Int. J. Heat Mass Transf..

[129] Langrish T.A.G., Kockel T.K. (2006). The assessment of a characteristic drying curve for milk powder for use in computational fluid dynamics modelling. Chem. Eng. J..

[130] Van Meel D.A. (1958). Adiabatic convection batch drying with recirculation of air. Chem. Eng. Sci..

[131] Keey R.B., Suzuki M. (1974). On the characteristic drying curve. Int. J. Heat Mass Transf..

[132] Keey R.B. (1991). Drying of Loose and Particulate Materials.

[133] Tran T.T.H., Jaskulski M., Tsotsas E. (2017). Reduction of a model for single droplet drying and application to CFD of skim milk spray drying. Dry. Technol..

[134] Wawrzyniak P., Jaskulski M., Zbiciński I., Podyma M. (2017). CFD modelling of moisture evaporation in an industrial dispersed system. Adv. Powder Technol..

[135] Woo M.W., Daud W.R.W., Mujumdar A.S., Talib M.Z.M., Hua W.Z., Tasirin S.M. (2008). Comparative study of droplet drying models for CFD modelling. Chem. Eng. Res. Des..

[136] Woo M.W., Daud W.R.W., Mujumdar A.S., Wu Z.H., Talib M.Z.M., Tasirin S.M. (2008). CFD evaluation of droplet drying models in a spray dryer fitted with a rotary atomizer. Dry. Technol..

[137] Patel K.C., Chen X.D. (2005). Prediction of spray-dried product quality using two simple drying kinetics models. J. Food Process. Eng..

[138] Huang L., Kumar K., Mujumdar A.S. (2004). Simulation of a spray dryer fitted with a rotary disk atomizer using a three-dimensional computional fluid dynamic model. Dry. Technol..

[139] Chen X.D., Xie G.Z. (1997). Fingerprints of the drying behaviour of particulate or thin layer food materials established using a reaction engineering model. Food Bioprod. Process. Trans. Inst. Chem. Eng. Part. C.

[140] Har C.L., Fu N., Chan E.S., Tey B.T., Chen X.D. (2017). Unraveling the droplet drying characteristics of crystallization-prone mannitol—Experiments and modeling. AIChE J..

[141] Putranto A., Chen X.D., Devahastin S., Xiao Z., Webley P.A. (2011). Application of the reaction engineering approach (REA) for modeling intermittent drying under time-varying humidity and temperature. Chem. Eng. Sci..

[142] Putranto A., Xiao Z., Chen X.D., Webley P.A. (2011). Intermittent drying of mango tissues: Implementation of the reaction engineering approach. Ind. Eng. Chem. Res..

[143] Putranto A., Chen X.D., Webley P.A. (2010). Infrared and convective drying of thin layer of polyvinyl alcohol (PVA)/glycerol/water mixture-The reaction engineering approach (REA). Chem. Eng. Process. Process. Intensif..

[144] Lin S.X.Q., Chen X.D. (2007). The reaction engineering approach to modelling the cream and whey protein concentrate droplet drying. Chem. Eng. Process. Process. Intensif..

[145] Rogers S., Wu W.D., Lin S.X.Q., Chen X.D. (2012). Particle shrinkage and morphology of milk powder made with a monodisperse spray dryer. Biochem. Eng. J..

[146] Fu N., Wai Woo M., Qi Lin S.X., Zhou Z., Dong Chen X. (2011). Reaction Engineering Approach (REA) to model the drying kinetics of droplets with different initial sizes-experiments and analyses. Chem. Eng. Sci..

[147] Patel K.C., Chen X.D., Lin S.X.Q., Adhikari B. (2009). A composite reaction engineering approach to drying of aqueous droplets containing sucrose, maltodextrin (DE6) and their mixtures. AIChE J..

[148] Jin Y., Chen X.D. (2009). Numerical study of the drying process of different sized particles in an industrial-scale spray dryer. Dry. Technol..

[149] Gianfrancesco A., Turchiuli C., Dumoulin E. (2008). Powder agglomeration during the spray-drying process: Measurements of air properties. Dairy Sci. Technol.

[150] Pearce D.L. (2006). A Novel Way to Measure the Concentration of a Spray in a Spray Dryer. Dry. Technol..

[151] Schutyser M.A.I., Perdana J., Boom R.M. (2012). Single droplet drying for optimal spray drying of enzymes and probiotics. Trends Food Sci. Technol..

[152] Ullum T., Sloth J., Brask A., Wahlberg M. (2010). Predicting spray dryer deposits by CFD and an empirical drying model. Dry. Technol..

[153] Both E.M., Boom R.M., Schutyser M.A.I. (2020). Particle morphology and powder properties during spray drying of maltodextrin and whey protein mixtures. Powder Technol..

[154] Krieger U.K., Marcolli C., Reid J.P. (2012). Exploring the complexity of aerosol particle properties and processes using single particle techniques. Chem. Soc. Rev..

[155] Pajander J.P., Matero S., Sloth J., Wan F., Rantanen J., Yang M. (2015). Raman mapping of mannitol/lysozyme particles produced via spray drying and single droplet drying. Pharm. Res..

[156] Both E.M., Karlina A.M., Boom R.M., Schutyser M.A.I. (2018). Morphology development during sessile single droplet drying of mixed maltodextrin and whey protein solutions. Food Hydrocoll..

[157] Walzel P. (2011). Influence of the spray method on product quality and morphology in spray drying. Chem. Eng. Technol..

[158] Elversson J., Millqvist-Fureby A. (2005). Particle size and density in spray drying—Effects of carbohydrate properties. J. Pharm. Sci..

[159] Vicente J., Pinto J., Menezes J., Gaspar F. (2013). Fundamental analysis of particle formation in spray drying. Powder Technol..

[160] Both E.M., Nuzzo M., Millqvist-Fureby A., Boom R.M., Schutyser M.A.I. (2018). Morphology development during single droplet drying of mixed component formulations and milk. Food Res. Int..

[161] Kemp I.C., Hartwig T., Herdman R., Hamilton P., Bisten A., Bermingham S. (2016). Spray drying with a two-fluid nozzle to produce fine particles: Atomization, scale-up, and modeling. Dry. Technol..

[162] Frackowiak B., Lavergne G., Tropea C., Strzelecki A. (2010). Numerical analysis of the interactions between evaporating droplets in a monodisperse stream. Int. J. Heat Mass Transf..

[163] Li H., Kuschel M., Sommerfeld M. (2016). Experimental Investigation and Modeling of Coalescence and Agglomeration for Spray Drying of Solutions. Process Spray - Functional Particles Produced in Spray Processes.

[164] Finotello G., Padding J.T., Buist K.A., Schijve A., Jongsma A., Innings F., Kuipers J.A.M. (2019). Numerical investigation of droplet-droplet collisions in a water and milk spray with coupled heat and mass transfer. Dry. Technol..

[165] Al-Dirawi K.H., Bayly A.E. (2020). An experimental study of binary collisions of miscible droplets with non-identical viscosities. Exp. Fluids.

[166] Ko G.H., Ryou H.S., Hur N.K., Ko S.W., Youn M.O. (2007). Numerical study on bouncing and separation collision between two droplets considering the collision-induced breakup. J. Mech. Sci. Technol..

[167] Charalampous G., Hardalupas Y. (2017). Collisions of droplets on spherical particles. Phys. Fluids.

[168] Mezhericher M., Levy A., Borde I. (2008). Droplet-droplet interactions in spray drying by using 2D computational fluid dynamic. Dry. Technol..

[169] Finotello G., Padding J.T., Buist K.A., Jongsma A., Innings F., Kuipers J.A.M. (2019). Droplet collisions of water and milk in a spray with Langevin turbulence dispersion. Int. J. Multiph. Flow.

[170] Littringer E.M., Paus R., Mescher A., Schroettner H., Walzel P., Urbanetz N.A. (2013). The morphology of spray dried mannitol particles-The vital importance of droplet size. Powder Technol..

[171] Smeets A., Koekoekx R., Clasen C., Van den Mooter G. (2018). Amorphous solid dispersions of darunavir: Comparison between spray drying and electrospraying. Eur. J. Pharm. Biopharm..

[172] Lee Y.-Y., Wu J.X., Yang M., Young P.M., Van Den Berg F., Rantanen J. (2011). Particle size dependence of polymorphism in spray-dried mannitol. Eur. J. Pharm. Sci..

[173] Le H.P. (1998). Progress and Trends in Ink-jet Printing Technology. J. Imaging Sci. Technol..

[174] Patel K.C., Chen X.D. (2007). Production of spherical and uniform-sized particles using a laboratory ink-jet spray dryer. Asia Pac. J. Chem. Eng..

[175] Berglund N., Liu B.Y.H. (1973). Generation of Monodisperse Aerosol Standards. Environ. Sci. Technol..

[176] Berkland C., Kim K., Pack D.W. (2001). Fabrication of PLG microspheres with precisely controlled and monodisperse size distributions. J. Control. Release.

[177] Islam M.I.U., Langrish T.A.G. (2010). The effect of different atomizing gases and drying media on the crystallization behavior of spray-dried powders. Dry. Technol..

[178] Littringer E.M., Mescher A., Eckhard S., Schröttner H., Langes C., Fries M., Griesser U., Walzel P., Urbanetz N.A. (2012). Spray drying of mannitol as a drug carrier-the impact of process parameters on product properties. Dry. Technol..

[179] Littringer E.M., Noisternig M.F., Mescher A., Schroettner H., Walzel P., Griesser U.J., Urbanetz N.A. (2013). The morphology and various densities of spray dried mannitol. Powder Technol..

[180] Kim E.H.-J., Dong Chen X., Pearce D. (2008). Surface composition of industrial spray-dried milk powders. 2. Effects of spray drying conditions on the surface composition. J. Food Eng..

[181] Dedroog S., Huygens C., Van den Mooter G. (2019). Chemically identical but physically different: A comparison of spray drying, hot melt extrusion and cryo-milling for the formulation of high drug loaded amorphous solid dispersions of naproxen. Eur. J. Pharm. Biopharm..

[182] Lechuga-Ballesteros D., Charan C., Stults C.L.M., Stevenson C.L., Miller D.P., Vehring R., Tep V., Kuo M.-C. (2008). Trileucine improves aerosol performance and stability of spray-dried powders for inhalation. J. Pharm. Sci..

[183] Rizi K., Green R., Donaldson M., Williams A. (2011). Production of pH-responsive microparticles by spray drying: Investigation of experimental parameter effects on morphological and release properties. J. Pharm. Sci..

[184] Wan F., Bohr A., Jonas Maltesen M., Bjerregaard S., Foged C., Rantanen J., Yang M. (2013). Critical solvent properties affecting the particle formation process and characteristics of Celecoxib-loaded PLGA microparticles via spray-drying. Pharm. Res..

[185] Clarke N., O’connor K., Ramtoola Z. (1998). Influence of formulation variables on the morphology of biodegradable microparticles prepared by spray drying. Drug Dev. Ind. Pharm..

[186] Bain D.F., Munday D.L., Smith A. (1999). Solvent influence on spray-dried biodegradable microspheres. J. Microencapsul..

[187] Wang F.J., Wang C.H. (2002). Sustained release of etanidazole from spray dried microspheres prepared by non-halogenated solvents. J. Control. Release.

[188] Baird J.A., Eerdenbrugh B.V.A.N., Taylor L.S. (2010). A Classification system to assess the crystallization tendency of organic molecules from undercooled melts. J. Pharm. Sci..

[189] Van Eerdenbrugh B., Baird J.A., Taylor L.S. (2010). Crystallization tendency of active pharmaceutical ingredients following rapid solvent evaporation—Classification and comparison with crystallization tendency from undercooled melts. J. Pharm. Sci..

[190] Xie J., Jiang J., Davoodi P., Srinivasan M.P., Wang C.H. (2015). Electrohydrodynamic atomization: A two-decade effort to produce and process micro-/nanoparticulate materials. Chem. Eng. Sci..

[191] Salata O. (2005). Tools of nanotechnology: Electrospray. Curr. Nanosci..

[192] Bohr A., Boetker J., Rades T., Rantanen J., Yang M. (2014). Application of spray-drying and electrospraying/electospinning for poorly watersoluble drugs: A particle engineering approach. Curr. Pharm. Des..

[193] Enayati M., Chang M.W., Bragman F., Edirisinghe M., Stride E. (2011). Electrohydrodynamic preparation of particles, capsules and bubbles for biomedical engineering applications. Colloids Surf. Physicochem. Eng. Asp..

[194] Boda S.K., Li X., Xie J. (2018). Electrospraying an enabling technology for pharmaceutical and biomedical applications: A review. J. Aerosol Sci..

[195] Cloupeau M., Prunet-Foch B. (1994). Electrohydrodynamic spraying functioning modes: A critical review. J. Aerosol Sci..

[196] Cloupeau M., Prunet-Foch B. (1990). Electrostatic spraying of liquids: Main functioning modes. J. Electrostat..

[197] Jaworek A., Krupa A. (1999). Classification of the modes of EHD spraying. J. Aerosol Sci..

[198] Sato M. (1984). The production of essentially uniform-sized liquid droplets in gaseous or immiscible liquid media under applied a.c. potential. J. Electrostat..

[199] Bodnár E., Grifoll J., Rosell-Llompart J. (2018). Polymer solution electrospraying: A tool for engineering particles and films with controlled morphology. J. Aerosol Sci..

[200] Zhang L., Huang J., Si T., Xu R.X. (2012). Coaxial electrospray of microparticles and nanoparticles for biomedical applications. Expert Rev. Med. Devices.

[201] Bocanegra R., Galán D., Márquez M., Loscertales I.G., Barrero A. (2005). Multiple electrosprays emitted from an array of holes. J. Aerosol Sci..

[202] Deng W., Waits C.M., Morgan B., Gomez A. (2009). Compact multiplexing of monodisperse electrosprays. J. Aerosol Sci..

[203] Deng W., Klemic J.F., Li X., Reed M.A., Gomez A. (2006). Increase of electrospray throughput using multiplexed microfabricated sources for the scalable generation of monodisperse droplets. Aerosol Sci..

[204] Deng W., Gomez A. (2007). Influence of space charge on the scale-up of multiplexed electrosprays. J. Aerosol Sci..

[205] Smeets A., Clasen C., Van den Mooter G. (2017). Electrospraying of polymer solutions: Study of formulation and process parameters. Eur. J. Pharm. Biopharm..

[206] Xie J., Lim L.K., Phua Y., Hua J., Wang C.H. (2006). Electrohydrodynamic atomization for biodegradable polymeric particle production. J. Colloid Interface Sci..

[207] Bock N., Dargaville T.R., Woodruff M.A. (2012). Electrospraying of polymers with therapeutic molecules: State of the art. Prog. Polym. Sci..

[208] Ghaeb M., Tavanai H., Kadivar M. (2015). Electrosprayed maize starch and its constituents (amylose and amylopectin) nanoparticles. Polym. Adv. Technol..

[209] Faramarzi A.R., Barzin J., Mobedi H. (2016). Effect of solution and apparatus parameters on the morphology and size of electrosprayed PLGA microparticles. Fibers Polym..

[210] Songsurang K., Praphairaksit N., Siraleartmukul K., Muangsin N. (2011). Electrospray fabrication of doxorubicin-chitosan-tripolyphosphate nanoparticles for delivery of doxorubicin. Arch. Pharm. Res..

[211] Tang K., Gomez A. (1996). Monodisperse electrosprays of low electric conductivity liquids in the cone-jet mode. J. Colloid Interface Sci..

[212] Zhang S., Kawakami K. (2010). One-step preparation of chitosan solid nanoparticles by electrospray deposition. Int. J. Pharm..

[213] Xu Y., Skotak M., Hanna M. (2006). Electrospray encapsulation of water-soluble protein with polylactide. I. Effects of formulations and process on morphology and particle size. J. Microencapsul..

[214] Hartman R.P.A., Brunner D.J., Camelot D.M.A., Marijnissen J.C.M., Scarlett B. (2000). Jet break-up in electrohydrodynamic atomization in the cone-jet mode. J. Aerosol Sci..

[215] de la Mora J.F., Loscertales I.G. (1994). The current emitted by highly conducting Taylor cones. J. Fluid Mech..

[216] Gañán-Calvo A.M., Dávila J., Barrero A. (1997). Current and droplet size in the electrospraying of liquids. Scaling laws. J. Aerosol Sci..

[217] Gomez-Estaca J., Balaguer M.P., Gavara R., Hernandez-Munoz P. (2012). Formation of zein nanoparticles by electrohydrodynamic atomization: Effect of the main processing variables and suitability for encapsulating the food coloring and active ingredient curcumin. Food Hydrocoll..

[218] Jafari-Nodoushan M., Barzin J., Mobedi H. (2015). Size and morphology controlling of PLGA microparticles produced by electro hydrodynamic atomization. Polym. Adv. Technol..

[219] Xie J., Marijnissen J.C.M., Wang C.H. (2006). Microparticles developed by electrohydrodynamic atomization for the local delivery of anticancer drug to treat C6 glioma in vitro. Biomaterials.

[220] Enayati M., Ahmad Z., Stride E., Edirisinghe M. (2010). Size mapping of electric field-assisted production of polycaprolactone particles. J. R. Soc. Interface.

[221] Almería B., Deng W., Fahmy T.M., Gomez A. (2010). Controlling the morphology of electrospray-generated PLGA microparticles for drug delivery. J. Colloid Interface Sci..

[222] Park C.H., Kim M.Y., Yoo J.Y., Kim K.H., Lee J., Lee J.C. (2007). Preparation of polymer/drug nano- and micro-particles by electrospraying. Macromol. Symp..

[223] Nguyen D.N., Palangetic L., Clasen C., Van den Mooter G. (2016). One-step production of darunavir solid dispersion nanoparticles coated with enteric polymers using electrospraying. J. Pharm. Pharmacol..

[224] Barron C., He J.-Q. (2017). Alginate-based microcapsules generated with the coaxial electrospray method for clinical application. J. Biomater. Sci. Polym. Ed..

[225] Gao Y., Zhao D., Chang M.W., Ahmad Z., Li X., Suo H., Li J.S. (2015). Morphology control of electrosprayed core-shell particles via collection media variation. Mater. Lett..

[226] Mehregan Nikoo A., Kadkhodaee R., Ghorani B., Razzaq H., Tucker N. (2016). Controlling the morphology and material characteristics of electrospray generated calcium alginate microhydrogels. J. Microencapsul..

[227] Bohr A., Wan F., Kristensen J., Dyas M., Stride E., Baldursdottír S., Edirisinghe M., Yang M. (2015). Pharmaceutical microparticle engineering with electrospraying: The role of mixed solvent systems in particle formation and characteristics. J. Mater. Sci. Mater. Med..

[228] Smeets A., Koekoekx R., Ruelens W., Smet M., Clasen C., Van den Mooter G. (2020). Gastro-resistant encapsulation of amorphous solid dispersions containing darunavir by coaxial electrospraying. Int. J. Pharm..

[229] Park C.H., Lee J. (2009). Electrosprayed polymer particles: Effect of the solvent properties. J. Appl. Polym. Sci..

[230] Ikeuchi M., Tane R., Ikuta K. (2012). Electrospray deposition and direct patterning of polylactic acid nanofibrous microcapsules for tissue engineering. Biomed. Microdevices.

[231] Zheng J., Zhang H., Zhao Z., Han C.C. (2012). Construction of hierarchical structures by electrospinning or electrospraying. Polymer.

[232] Bodnár E., Rosell-Llompart J. (2013). Growth dynamics of granular films produced by electrospray. J. Colloid Interface Sci..

[233] Bock N., Woodruff M.A., Hutmacher D.W., Dargaville T.R., Bock N., Woodruff M.A., Hutmacher D.W., Dargaville T.R. (2011). Electrospraying, a reproducible method for production of polymeric microspheres for biomedical applications. Polymers.

[234] Yu J.H., Fridrikh S.V., Rutledge G.C. (2006). The role of elasticity in the formation of electrospun fibers. Polymer.

[235] Shenoy S.L., Bates W.D., Frisch H.L., Wnek G.E. (2005). Role of chain entanglements on fiber formation during electrospinning of polymer solutions: Good solvent, non-specific polymer–polymer interaction limit. Polymer.

[236] Palangetic L., Reddy N.K., Srinivasan S., Cohen R.E., McKinley G.H., Clasen C. (2014). Dispersity and spinnability: Why highly polydisperse polymer solutions are desirable for electrospinning. Polymer.

[237] Srinivasan S., Chhatre S.S., Mabry J.M., Cohen R.E., McKinley G.H. (2011). Solution spraying of poly(methyl methacrylate) blends to fabricate microtextured, superoleophobic surfaces. Polymer.

[238] Gupta P., Elkins C., Long T.E., Wilkes G.L. (2005). Electrospinning of linear homopolymers of poly(methyl methacrylate): Exploring relationships between fiber formation, viscosity, molecular weight and concentration in a good solvent. Polymer.

[239] Koekoekx R., Zawacka N.C., Van den Mooter G., Hens Z., Clasen C. (2020). Electrospraying the Triblock Copolymer SEBS: The Effect of Solvent System and the Embedding of Quantum Dots. Macromol. Mater. Eng..

[240] Shenoy S.L., Bates W.D., Wnek G. (2005). Correlations between electrospinnability and physical gelation. Polymer.

[241] Festag R., Alexandratos S.D., Joy D.C., Wunderlich B., Annis B., Cook K.D. (1998). Effects of molecular entanglements during electrospray of high molecular weight polymers. J. Am. Soc. Mass Spectrom..

[242] Jayasinghe S.N., Edirisinghe M.J. (2002). Effect of viscosity on the size of relics produced by electrostatic atomization. J. Aerosol Sci..

[243] Rosell-Llompart J., de la Mora J.F. (1994). Generation of monodisperse droplets 0.3 to 4 μm in diameter from electrified cone-jets of highly conducting and viscous liquids. J. Aerosol Sci..

[244] Yao L., Haas T.W., Guiseppi-Elie A., Bowlin G.L., Simpson D.G., Wnek G.E. (2003). Electrospinning and stabilization of fully hydrolyzed poly(vinyl alcohol) fibers. Chem. Mater..

[245] Xue L., Mao L., Cai Q., Yang X., Jin R. (2011). Preparation of amino acid ester substituted polyphosphazene microparticles via electrohydrodynamic atomization. Polym. Adv. Technol..

[246] Yao J., Kuang Lim L., Xie J., Hua J., Wang C.H. (2008). Characterization of electrospraying process for polymeric particle fabrication. J. Aerosol Sci..

[247] Meng F., Jiang Y., Sun Z., Yin Y., Li Y. (2009). Electrohydrodynamic liquid atomization of biodegradable polymer microparticles: Effect of electrohydrodynamic liquid atomization variables on microparticles. J. Appl. Polym. Sci..

[248] Zhou S., Cook K.D. (2000). Probing solvent fractionation in electrospray droplets with laser- induced fluorescence of a solvatochromic dye. Anal. Chem..

[249] Deprédurand V., Delconte A., Lemoine F. (2011). Combined PDA and LIF applied to size-temperature correlations measurements in a heated spray. Exp. Fluids.

[250] Vetrano M.R., Simonini A., Steelant J., Rambaud P. (2013). Thermal characterization of a flashing jet by planar laser-induced fluorescence this article is part of the topical collection on application of laser techniques to fluid mechanics. Exp. Fluids.

[251] Desantes J.M., Pastor J.V., Pastor J.M., Juliá J.E. (2005). Limitations on the use of the planar laser induced exciplex fluorescence technique in diesel sprays. Fuel.

[252] Wu X., Wu Y., Saengkaew S. (2012). Concentration and composition measurement of sprays with a global rainbow technique. Meas. Sci. Technol..

[253] Stowers M.A., Friedlander S.K., Stowers M.A., Friedlander S.K. (2010). Chemical characterization of flowing polydisperse aerosols by raman spectroscopy chemical characterization of flowing polydisperse aerosols by raman spectroscopy. Aerosol Sci. Technol..

[254] Vehring R., Schweiger G. (1996). Raman thermometry of aqueous multicomponent aerosol particles. J. Aerosol Sci..

[255] Müller T., Grünefeld G., Beushausen V. (2000). High-precision measurement of the temperature of methanol and ethanol droplets using spontaneous Raman scattering. Appl. Phys. B Lasers Opt..

[256] Raffel M., Willert C.E., Wareley S.T., Kompenhans J. (2007). Particle Image Velocimetry: A Practical Guide.

[257] Poozesh S., Grib S.W., Renfro M.W., Marsac P.J. (2018). Near-field dynamics of high-speed spray dryer coannular two fluid nozzle: Effects of operational conditions and formulations. Powder Technol..

[258] Liu X., Doub W.H., Guo C. (2011). Assessment of the influence factors on nasal spray droplet velocity using Phase-Doppler Anemometry (PDA). AAPS PharmSciTech.

[259] Husted B.P., Petersson P., Lund I., Holmstedt G. (2009). Comparison of PIV and PDA droplet velocity measurement techniques on two high-pressure water mist nozzles. Fire Saf. J..

[260] Tropea C. (2011). Optical Particle Characterization in Flows. Annu. Rev. Fluid Mech..

[261] Lin S.M., Waterman D.R., Lettington A.H. (2000). Measurement of droplet velocity, and refractive index using the pulse displacement technique. Meas. Sci. Technol..

[262] Yang Y., Kang B. (2009). Measurements of the characteristics of spray droplets using in-line digital particle holography. J. Mech. Sci. Technol..

[263] Na C.A.O., Liang C.A.O., Changcai H.A.N., Qing X.U., Lan L.E.I., Jiye D. (2012). A study of the spray characterization from a centrifugal nozzle by pulsed laser holography. Appl. Mech. Mater..

[264] Mosén K., Bäckström K., Thalberg K., Schaefer T., Kristensen H.G., Axelsson A. (2004). Particle formation and capture during spray drying of inhalable particles. Pharm. Dev. Technol..

[265] Ku B.K., Kim S.S., Kim Y.D., Lee S.Y. (2001). Direct measurement of electrospray droplets in submicron diameter using a freezing method and a TEM image processing technique. J. Aerosol Sci..

[266] Suh Y.J., Lee J.W., Chang H., Jang H.D., Cho K. (2013). Non-spherical particle formation induced by repulsive hydration forces during spray drying. J. Nanoparticle Res..

[267] Boel E., Smeets A., Vergaelen M., De La Rosa V.R., Hoogenboom R., Van den Mooter G. (2019). Comparative study of the potential of poly(2-ethyl-2-oxazoline) as carrier in the formulation of amorphous solid dispersions of poorly soluble drugs. Eur. J. Pharm. Biopharm..

[268] Liu X., Feng X., Williams R.O., Zhang F. (2018). Characterization of amorphous solid dispersions. J. Pharm. Investig..

[269] Guo Y., Shalaev E., Smith S. (2013). Physical stability of pharmaceutical formulations: Solid-state characterization of amorphous dispersions. TrAC Trends Anal. Chem..

[270] Varga C., Lasheras J., Hopfinger E. (2003). Initial breakup of a small-diameter liquid jet by a high-speed gas stream. J. Fluid Mech..

[271] Li X., Li M. (2003). Droplet size distribution in sprays based on maximization of entropy generation. Entropy.

[272] Kemp I.C., Oakley D.E. (2002). Modelling of particulate drying in theory and practice. Dry. Technol..

[273] Pinto M., Kemp I.C., Bermingham S., Hartwig T., Bisten A. (2014). Development of an axisymmetric population balance model for spray drying and validation against experimental data and CFD simulations. Chem. Eng. Res. Des..

